# IGF/mTORC1/S6 Signaling Is Potentiated and Prolonged by Acute Loading of Subtoxicological Manganese Ion

**DOI:** 10.3390/biom13081229

**Published:** 2023-08-08

**Authors:** Xueqi Tang, Rekha C. Balachandran, Michael Aschner, Aaron B. Bowman

**Affiliations:** 1 School of Health Sciences, Purdue University, West Lafayette, IN 47907, USA; tang484@purdue.edu (X.T.);; 2 Exponent Inc., Alexandria, VA 22314, USA; 3 Department of Molecular Pharmacology, Albert Einstein College of Medicine, Bronx, NY 10461, USA

**Keywords:** manganese, insulin-like growth factor/insulin signaling, mammalian target of rapamycin complex 1, striatal neuronal cells

## Abstract

The insulin-like growth factor (IGF)/insulin signaling (IIS) pathway is involved in cellular responses against intracellular divalent manganese ion (Mn^2+^) accumulation. As a pathway where multiple nodes utilize Mn^2+^ as a metallic co-factor, how the IIS signaling patterns are affected by Mn^2+^ overload is unresolved. In our prior studies, acute Mn^2+^ exposure potentiated IIS kinase activity upon physiological-level stimulation, indicated by elevated phosphorylation of protein kinase B (PKB, also known as AKT). AKT phosphorylation is associated with IIS activity; and provides direct signaling transduction input for the mammalian target of rapamycin complex 1 (mTORC1) and its downstream target ribosomal protein S6 (S6). Here, to better define the impact of Mn^2+^ exposure on IIS function, Mn^2+^-induced IIS activation was evaluated with serial concentrations and temporal endpoints. In the wild-type murine striatal neuronal line ST*Hdh*, the acute treatment of Mn^2+^ with IGF induced a Mn^2+^ concentration-sensitive phosphorylation of S6 at Ser235/236 to as low as 5 μM extracellular Mn^2+^. This effect required both the essential amino acids and insulin receptor (IR)/IGF receptor (IGFR) signaling input. Similar to simultaneous stimulation of Mn^2+^ and IGF, when a steady-state elevation of Mn^2+^ was established via a 24-h pre-exposure, phosphorylation of S6 also displayed higher sensitivity to sub-cytotoxic Mn^2+^ when compared to AKT phosphorylation at Ser473. This indicates a synergistic effect of sub-cytotoxic Mn^2+^ on IIS and mTORC1 signaling. Furthermore, elevated intracellular Mn^2+^, with both durations, led to a prolonged activation in AKT and S6 upon stimulation. Our data demonstrate that the downstream regulator S6 is a highly sensitive target of elevated Mn^2+^ and is well below the established acute cytotoxicity thresholds (<50 μM). These findings indicate that the IIS/mTORC1 pathways, in which Mn^2+^ normally serves as an essential co-factor, are dually responsible for the cellular changes in exposures to real-world Mn^2+^ concentrations.

## 1. Introduction

The essential metal manganese (Mn^2+^) serves as a dependent or high-affinity co-factor for multiple kinases. Under normal homeostatic conditions, Mn^2+^ binds to protein kinase active sites and functions as a bridge between the substrate and the kinase, as well as increases the affinity of the kinase to ATP [1]. Compared to the most utilized metallic enzyme co-factor, magnesium ion (Mg^2+^), Mn^2+^ can facilitate protein conformational changes as a slightly larger ion and a softer Lewis acid [2,3] and therefore is preferred by specific kinases in their catalytic processes. These kinases, including manganese-superoxide dismutase (MnSOD), ataxia-telangiectasia mutated (ATM), target of rapamycin complex 1 (TORC1, mTORC1 in mammals), leucine-rich repeat kinase 2 (LRRK2, also known as PARK8), and phosphatidylinositol 3-kinases (PI3K), are positioned at key nodes of neuronal functional and metabolic regulations [4,5,6,7]. Adverse outcomes associated with dysregulations of these kinases, such as oxidative stress, glucose uptake deficiency, glutamate transportation dysfunction, and microtubule development interruption, contribute to the linkage between susceptibility to neurodegenerative diseases and excessive brain Mn^2+^ accumulation. Thus, Mn^2+^ content is strictly regulated, with cerebrospinal fluid concentrations estimated at 40–50 nM [8,9]. While exposed human brain Mn^2+^ distribution has not been fully characterized, studies in monkeys suggested that a ~3-fold increase of brain Mn^2+^ led to severe neurological symptoms within weeks [10,11]. However, this rapid development of neurological dysfunction does not reflect the risk of elevated Mn^2+^ neurotoxicity in humans, which is a consequence of accumulation across much longer time frames, yet much lower exposure levels. This lack of representation of real-world scenarios led us to evaluate the cellular response to threshold levels of Mn^2+^ exposure elevated to just above the nominal essential levels and well below those associated with acute cytotoxicity and cell death.

The threshold Mn^2+^ exposure levels in this study were defined based on the available reports in cultured cell models. Our prior study with a murine striatal neuronal line (ST*Hdh*) suggested that 24 h (h) of exposure to 50 μM Mn^2+^ and below in a culture medium did not induce a detectable increase of apoptotic markers or a loss of cell viability [12,13]. Similarly, 24-h exposure to 100 μM Mn^2+^ did not affect the viability of cortical, striatal, or midbrain human-induced pluripotent stem cell (hiPSCs)-derived neuroprogenitor cells [14]. Another study, which examined the effects of Mn^2+^ exposure on a human neuroblastoma line (SH-SY5Y), reported no cell death following exposures to 10 or 50 μM Mn^2+^ for 5 h, while it detected substantial cell death in the 50 μM Mn^2+^-treated group after 24 h of recovery [15,16]. Therefore, we define 50 μM as a threshold level of in vitro cytotoxicity, and extracellular Mn^2+^ concentrations below 50 μM are considered as low just-above threshold overload levels of extracellular Mn^2+^ in this manuscript.

Cellular responses to Mn^2+^ exposures are not fully understood, though mitochondrial dysfunction is acknowledged to be one of the leading outcomes [17]. However, our study by Warren et al. argued against the mitochondria-driven mechanisms in threshold-level conditions, where neither cellular energy usage nor the oxygen consumption rate (OCR) were affected by 24-h exposure to 50 μM Mn^2+^ [12]. Based upon the nature of the insulin-like growth factor (IGF)/insulin signaling (IIS) pathway, where multiple kinases utilize Mn^2+^ as an essential co-factor, as well as the established linkage between IIS and Mn^2+^ overload, we hypothesized that the IIS pathway may be among the most sensitive respondents to Mn^2+^ neurotoxicity, as intracellular Mn^2+^ begins to rise beyond normal homeostatic levels for normal function.

As mentioned, the IIS response has been confirmed to be involved in Mn^2+^-induced acute neuronal damage [18,19,20]. Our prior study by Bryan et al. (2020) revealed, for the first time, the direct interaction between elevated Mn^2+^ and IGF receptors (IGFR), indicated by a synergistic potentiation on IGFR and protein kinase B (PKB, known as and referred to as AKT) phosphorylation, triggered by the simultaneous treatment of 200 μM Mn^2+^ and a physiological concentration of IGF [21]. However, this synergism was focused on the upstream response under toxic concentrations of Mn^2+^, leaving the downstream effects under lower-concentration-Mn^2+^ treatments unknown. As mentioned, the preference of multiple kinases along the IIS pathway suggests the sensitivity of IIS to minor Mn^2+^ content fluctuation. Under cell-free conditions, Mn^2+^ more efficiently facilitates the direct binding of IGF to integrin compared to Mg^2+^ at the same concentration in the context of integrin/IGFR ternary active signaling complexes [22]. Following the binding of IGF to its receptor, Mn^2+^ further promotes the autophosphorylation of insulin receptor (IR) and IGFR through both enhancing kinase activity and inhibiting receptor dephosphorylation [23]. Activation of IR and IGFR initiates the phosphorylation of insulin receptor substrate (IRS), thus recruiting and activating PI3K, which catalyzes the conversion of second messenger PIP2 to PIP3. This cascade results in AKT phosphorylation and consequently activates TORC1. TORC1 activity, a central hub of IIS, is boosted by the physiological level of Mn^2+^, and consequently, induces phosphorylation of its downstream targets ribosomal protein S6 (S6) and eukaryotic translation initiation factor 4E (eIF4E)-binding protein 1 (4E-BP1) [6,24].

The essentiality of Mn^2+^ in IIS transduction supported our hypothesis in a theoretical way, and several studies were probative with evidence that kinase activities along the IIS pathway can be altered by a moderate elevation of brain or neuronal Mn^2+^ content. Srivastava et al. confirmed AKT/mTORC1 activation in female rat hypothalamus induced by early-life Mn^2+^ dosing (17 days) after a moderate increase of brain Mn^2+^ (~1.3-fold) [25]. Cheng et al. showed a moderate increase in hippocampal PI3K/AKT activity in rats exposed to low-dose chronic Mn^2+^ (5.0 mg/kg, 24 weeks) [26]. Our lab has also demonstrated that, at the threshold Mn^2+^ concentration (50 μM, 24 h), without inducing detectable viability loss or apoptosis, the activity of AKT is still significantly elevated [12,21].

Taken together, the available research provides a compelling basis for the hypothesis that the IIS pathway is perhaps one of the most, if not the most, sensitive primary targets of threshold-level Mn^2+^ overload, e.g., the first to be affected, as the Mn^2+^ levels exceed the levels required for the essential functions of Mn^2+^. The significance of further characterization of the IIS response is that it reflects the capability of the IIS pathway dynamics to growth factor stimulation, which is a critical function of the pathway, and its failure is closely related to the development of neurodegeneration. In this study, we sought to confirm the IIS responsiveness with low-level Mn^2+^ exposure, as well as characterize the sensitivity of various kinases to the Mn^2+^ associated with this pathway.

## 2. Methods

### 2.1. Cell Culture

The immortalized, murine striatal cell line, ST*Hdh*^Q7/Q7^ (Q7), was obtained from the Coriell Cell Repository (Camden, NJ, USA). The Q7 cells were cultured in Dulbecco’s Modified Eagle Medium (DMEM, D6546 Sigma-Aldrich, St. Louis, MO, USA), supplemented with 10% FBS (Atlanta Biologicals, Flowery Branch, GA, USA), 2 mM GlutaMAX, 100 U/mL of Penicillin–Streptomycin, 0.5 mg/mL of G418 Sulfate, 1× MEM non-essential amino acids solution, and 14 mM HEPES (all cell culture supplements were purchased from Life Technologies, Carlsbad, CA, USA). Cells were maintained under 5% CO_2_ at 33 °C—the optimal growing temperature for the Q7s—and sub-cultured before reaching 80% confluency via incubation in a 0.05% Trypsin-EDTA solution (Life Technologies, Carlsbad, CA, USA) for 5 min (min) at 33 °C.

At 16–18 h prior to exposure, the cells were plated in a designated cell culture plate at 8 × 10^4^ cells/mL. For exposures of less than 24 h, the cells were washed 3 times with Earle’s Balanced Salts with Sodium (EBSS, E3024 Sigma-Aldrich, St. Louis, MO, USA) and subject to serum deprivation in the indicated buffer for an hour. Following deprivation, the cells were exposed to 0–200 μM Mn^2+^ (MnCl_2_) with the indicated supplements in fresh buffer.

For the 24-h pre-exposure, Q7 cells were exposed to 0, 0.5, 5, or 50 μM Mn^2+^ in culture media 16–18 h after plating. After 24 h of pre-exposure, the cells went through 1 h of serum deprivation and treatment in the designated buffer for up to 6 h. All Mn^2+^ exposures were applied extracellularly, and the Mn^2+^ concentrations represented the Mn^2+^ content in the medium that the cells were incubated in.

### 2.2. Western Blot

By the end of the treatments, the cells were washed once with 1× phosphate-buffered saline (PBS, Fisher Scientific, Waltham, MA, USA) and scraped from a 6-well culture plate with RIPA buffer, supplemented with protease inhibitor and phosphatase inhibitor cocktails 2 and 3 (Sigma, Sigma-Aldrich, St. Louis, MO, USA). Cell lysates were centrifuged at 15,000× *g* for 15 min at 4 °C, and the supernatant was transferred into a new tube for processing. The total protein concentration was determined by the Pierce BCA protein assay kit (Fisher Scientific, Waltham, MA, USA). Loading samples were prepared by mixing the cell lysates with 4× Laemmli sample buffer (Bio-Rad Laboratories, Hercules, CA, USA) containing 1% 2-mercaptoethanol and boiled for 5 min at 95 °C. A total of 20 μg total protein was loaded for each sample onto a 4–15% pre-cast SDS-PAGE gel (Bio-Rad, Hercules, CA, USA) and run at 100 V for 110 min. The protein bands were then transferred onto nitrocellulose membranes using the iBlot Gel Transfer Device (Life Technologies). The blots were blocked with 5% non-fat dry milk in 1× Tris-buffered saline (TBS) for 1 h at room temperature and incubated with primary antibodies in 1× TBS containing 0.1% Tween-20 (0.1% TBST) and 5% BSA at 4 °C overnight.

Unbound primary antibodies were removed by three 10-min washes with 0.1% TBST. This was followed by secondary antibody incubation in Odyssey TBS blocking buffer with 0.1% Tween-20 for 1 h at room temperature. After three final washes of 10 min each, the blots were scanned with the LI-COR Odyssey 9120 Infrared imaging system and quantified with Image Studio (LI-COR, Lincoln, NE, USA).

For all investigated targets, antibodies against both the total protein and phosphorylation at specific residue(s) were probed simultaneously. The specificity of the antibodies was verified by individual probing, and the molecular weight of each target protein was confirmed based on protein ladders and total protein staining. No significant differences were detected in the total AKT or S6, regardless of the treatments. Therefore, their phosphorylation levels were quantified by the intensity of targeted proteins phosphorylated at key activity sites (referred to as p-, with the phosphorylated site specified) normalized to the total (referred to as PAN) target protein intensity. A list of all antibodies used in this study can be found in Supplementary Table S1.

Note, however, that the quantitative figures were created with the most relevant experimental conditions, while the sample loading was aimed at maximizing the inclusion of the treatments on the gels. Thus, some representative blots do not have the order of samples aligned with the quantifications. However, all westerns were analyzed within the same gel and were only cut/copied for simplicity and space.

### 2.3. Cellular Fura-2 Manganese Uptake Assay (CFMEA)

A CFMEA was performed to determine the post-exposure intracellular Mn^2+^ levels as previously described [27]. In brief, the cells were seeded in a 96-well plate prior to the experiment. Following the exposure, cells were lysed in PBS with 0.1% Triton X-100 containing 0.5 μM Fura-2. The Fura-2 fluorescence was measured at Ex_360_/Em_535,_ and the total extracted intracellular Mn^2+^ was calculated through an established cell-free Mn^2+^-Fura-2 standard curve.

## 3. Results

### 3.1. S6 Phosphorylation Displayed a Higher Sensitivity to Lower Levels of Mn^2+^ Exposure

**Figure 1:**
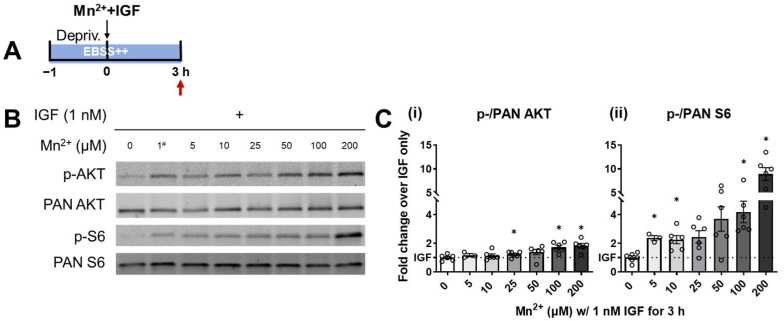
AKT and S6 show a Mn^2+^-concentration-dependent elevation in response to simultaneous treatment of 0–200 μM Mn^2+^ with 1 nM IGF. (**A**) Schematic indication of treatment paradigm. The red arrow indicate the timepoint when samples were collected. ST*Hdh*^Q7/Q7^ cell lysates were collected after 1 h serum deprivation with 3 h treatment of Mn^2+^ and IGF in EBSS++. Depriv. = serum deprivation. EBSS++ = Earle’s Balanced Salt Solution (EBSS) supplied with 1× Glutamax, 1× non-essential amino acids, 14 mM HEPES (+), and 1× essential amino acids (+). (**B**) Representative western blots of AKT phosphorylated at Serine (Ser) 473 residue (labeled as p-AKT), total AKT (regardless of phosphorylation status labeled as PAN AKT), S6 phosphorylated at Serine 235/236 residues (labeled as p-S6), and total S6 (regardless of phosphorylation status labeled as PAN S6). # The group treated with 1 nM IGF and 1 µM MnCl2 was utilized as a positive loading control and therefore not included in the quantifications. (**C**) Quantification of fold-change over IGF-only treatment was calculated by normalizing p-/PAN AKT (**i**) or p-/PAN S6 (**ii**) band intensity ratios to a positive control within each biological replicate. Bars are plotted as mean ± SEM (N = 6). p-/PAN AKT: mixed-effects one-way ANOVA; treatment = *F* (2.213, 11.80) = 5.618; *p* = 0.0174. p-/PAN S6: mixed-effects one-way ANOVA; treatment = *F* (1.855, 8.349) = 18.96; *p* = 0.0009. * *p* < 0.05 in the comparison of indicated concentrations of Mn^2+^ with IGF to no Mn^2+^ with IGF treatment by mixed-effect analysis with the Geisser–Greenhouse correction, followed by Dunnett’s multiple comparisons test.

The Q7 cell line was utilized in this study as an in vitro model for exploring the IIS responses to Mn^2+^ exposures. Q7 is a wild-type murine neuronal line developed from the mouse primary striatum. The striatum has been characterized as a targeted site of Mn^2+^ accumulation in both murine and human brains upon exposure, therefore making the Q7 line suitable for evaluating Mn^2+^ neurotoxicity [28,29]. In our prior study, where the synergistic effect of Mn^2+^ and IGF on IIS was confirmed, we applied cytotoxic concentrations (200 and 500 μM) of Mn^2+^, resulting in an over 100-fold increase in intracellular Mn^2+^ content [21]. Here, to investigate the IIS kinase responses to human toxicological and environmentally relevant levels of Mn^2+^, we evaluated the phosphorylated/total (p-/PAN) AKT and p-/PAN S6 ratio in Q7 cells with serial concentrations of Mn^2+^ (5–200 μM) accompanied by co-treatment with physiological levels of IGF (1 nM). This range of extracellular Mn^2+^ concentrations covered the estimated human cerebrospinal fluid level (~20–55 μM, Bowman and Aschner, 2014) and the in vitro cytotoxicity threshold that we identified (50 μM), as well as a higher toxic level that aligned with Bryan et al. To maximize the sensitivity of IIS kinases, a 1-h serum deprivation was applied prior to any treatment to bring the activity levels of AKT and S6 to baseline, as we have previously reported [21]. Upon 1-h serum deprivation, a 3-h Mn^2+^ treatment was carried out with IGF supplementation. Both AKT and S6 were activated by IGF itself, with a significant increase in the p-/PAN ratios. Exposure to Mn^2+^ did not significantly augment the effect of IGF stimulation alone on AKT phosphorylation until its concentration reached 25 μM and above (Figure 1C(i), N = 6). In contrast, S6 phosphorylation was strongly boosted in a concentration-dependent manner from exposure to Mn^2+^ concentrations as low as 5 μM (Figure 1C(ii), N = 6). These results suggest a higher sensitivity of S6 responding to the threshold levels of Mn^2+^ exposure with IGF present compared to AKT. Given that S6 is a downstream target of PI3K/AKT/mTORC1 signaling and predominantly requires input from AKT, it was counterintuitive that S6 was activated by Mn^2+^ at concentrations below a detectable AKT-response induction. This differential sensitivity led us to test whether the intermediate regulators of S6 could also be involved in the response to low-concentration Mn^2+^ exposure (e.g., a pathway parallel to AKT).

### 3.2. Essential Amino Acids Supplementation and IGF/Insulin Signaling Are Required for AKT and S6 Response to Low-Level Mn^2+^ Exposure

**Figure 2:**
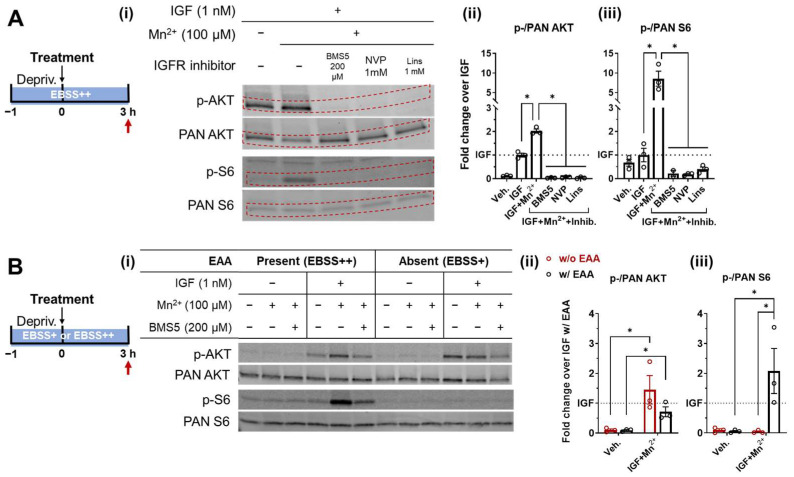
The synergistic effect of Mn^2+^ with IGF on AKT activation requires the involvement of IGF receptors, while S6 activation requires input from both IGF receptors and essential amino acids supplementation. (**A**) Schematic plot shows the treatment paradigm, with the red arrow indicating sample collection timepoint. ST*Hdh*^Q7/Q7^ cells were treated with the same timeline as described in A with Mn^2+^, Mn^2+^ + IGF, or Mn^2+^ + IGF + indicated IGF receptor inhibitor in EBSS++. Representative blots are labeled for each primary antibody on the left (**i**) and quantifications of p-/PAN AKT (**ii**) and p-/PAN S6 (**iii**) expression ratios. For (**A**(**i**)), there was a distortion of the bands due to electrophoresis effects—the anticipated position of the band is indicated between the dashed red lines based on the total protein stain for the blot. Plus and minus signs in the table above indicate whether the cells were treated with (+) or without (−) specific ingredient. Fold change, calculated by normalizing band intensities to positive control band within the same blot of the same biological replicate, followed by average expression ratio with IGF-only treatment set as 1. p-/PAN AKT bars are plotted as mean ± SEM (N = 3); one-way ANOVA; treatment = *F* (5, 12) = 264.2; *p* < 0.0001. p-/PAN S6 bars are plotted as mean ± SEM (N = 3); one-way ANOVA; treatment = *F* (5, 11) = 15.70; *p* = 0.0001. * *p* < 0.05 with ordinary one-way ANOVA followed by Dunnett’s multiple comparisons with a single pooled variance. Depriv. = serum deprivation. EBSS+ = Earle’s Balanced Salt Solution (EBSS) supplied with 1× Glutamax, 1× non-essential amino acids, and 14 mM HEPES. EBSS++ = EBSS+ supplied with 1× EAA. BMS5 = BMS-536924; NVP = NVP-AEW541; Lins = Linsitinib. (**B**) Schematic plot shows the treatment paradigm, with the red arrow indicating sample collection timepoint. ST*Hdh*^Q7/Q7^ cell lysates were collected after a 1 h serum deprivation followed by a 3 h treatment of Mn^2+^ with IGF in EBSS+ supplied with or without essential amino acids (EAA). Representative blots (**i**) and quantifications of the p-/PAN AKT (**ii**) and p-/PAN S6 (**iii**) expression ratios. Fold changes were calculated by normalizing band intensities to a positive control band (w/EAA IGF + Mn^2+^ + BMS5 + band) within the same blot of the same biological replicate, followed by average IGF-only treatment with EAA supplement expression ratio set as 1. p-/PAN AKT bars are plotted as mean ± SEM (N = 3); two-way ANOVA; treatment = *F* (1, 8) =15.10; *p* = 0.0046; EAA = *F* (1, 8) = 2.145; *p* = 0.1812; treatment-EAA interaction = *F* (1, 8) = 2.067; *p* = 0.1884. p-/PAN S6 bars are plotted as mean ± SEM (N = 3); two-way ANOVA; treatment = *F* (1, 8) = 6.751; *p* = 0.0317; EAA = *F* (1, 8) = 6.955; *p* = 0.0298; treatment-EAA interaction = *F* (1, 8) = 7.504; *p* = 0.0255. * *p* < 0.05 with ordinary two-way ANOVA followed by Bonferroni’s multiple comparisons with a single pooled variance.

S6 phosphorylation is directly regulated by mTORC1, which senses environmental cues, with growth factors and amino acids being the key promoters. Therefore, our investigation was divided into two parts, with the role of IGF input first examined. Growth factor-induced mTORC1 phosphorylation is mediated by AKT inactivation of the tuberous sclerosis complex (TSC), thus inhibiting the downstream Ras homolog enriched in brain (Rheb) and consequently releasing and activating mTORC1. With the application of pharmacological inhibitors of the IR/IGFR, the AKT and S6 activity was evaluated in comparison to IGF or Mn^2+^ with IGF stimulation. Synergistic potentiation was confirmed for both AKT and S6 following the 1-h deprivation and 3-h treatment of Mn^2+^ (100 μM) with IGF (1 nM). IGFR inhibitors, including BMS-536924 (BMS5, 200 μM), NVP-AEW541 (NVP, 1 mM), and Linsitinib (Lins, also known as OSI-906, 1 mM), completely suppressed the stimulation in both AKT and S6 (Figure 2A). With the application of multiple IR/IGFR inhibitors, we were able to cover the off-target effects of each individual inhibitor via different aspects, thus increasing our confidence in proving that there was no detectable effect of Mn^2+^ + IGF on either AKT or S6 phosphorylation when IR/IGFR were blocked. This finding is consistent with our prior publications, showing the Mn^2+^ effect on AKT as being IR/IGFR-dependent [21]. Combining these results with our findings that the Mn^2+^-induced increase in p-S6 was blocked by PI3K inhibitors, we excluded the possibility that higher responsiveness of p-S6 to Mn^2+^ + IGF stimulation compared to p-AKT can be explained by the effects of parallel pathways that activate S6 bypassing AKT phosphorylation.

A key metabolic state input for mTORC1/S6 activation, specifically the availability of amino acids, was also examined. mTORC1 activation is initiated by the translocation of mTORC1 to the lysosomal surface, mediated by sensing the supplementation of amino acids. On the lysosomal surface, the signaling is mediated by Ras-related GTP binding (Rag) GTPases [30,31]. The shift of the Rag GTPases to their active nucleotide-bound state is triggered by the addition of amino acids, especially leucine and arginine [32]. Activated Rag GTPases further reside in the direct mTORC1 activator Rheb. Finally, the association of mTORC1 with Rheb leads to mTORC1 activation [33]. In contrast, amino acid deprivation converts the Rag GTPases to their inactive state, which releases mTORC1 from the lysosomal surface, thus shutting down mTORC1 activity. As shown in Figure 2B(iii), when deprived of essential amino acids (EAA), the S6 responses to both IGF and Mn^2+^ with IGF stimulation are completely abrogated, indicating that Mn^2+^ and IGF stimulation on the mTORC1/S6 pathway requires a permissive state of mTORC1 (i.e., sufficient EAA status). In turn, this strongly suggests that there is no effect of Mn^2+^ + IGF downstream of mTORC1 on the phosphorylation of S6 (e.g., S6 kinase). Compared to the deactivation of S6, AKT phosphorylation is induced by IGF and Mn^2+^ with IGF, despite EAA supplementation (Figure 2B(ii)). We also noticed that the magnitude of the AKT response to Mn^2+^ + IGF was trending to a slight increase (but not reaching the statistical significance threshold of 0.05) by mTORC1 deactivation under the lack of EAA, which would be consistent with a loss of negative feedback input by mTORC1 on AKT.

Taken together, these results support a hypothesis that mTORC1 is a highly sensitive respondent in the Q7-cellular signaling response to simultaneous exposures to Mn^2+^ and IGF.

### 3.3. Mn^2+^ Exposure Prolongs AKT and S6 Responses to Physiological IGF Stimulation

**Figure 3:**
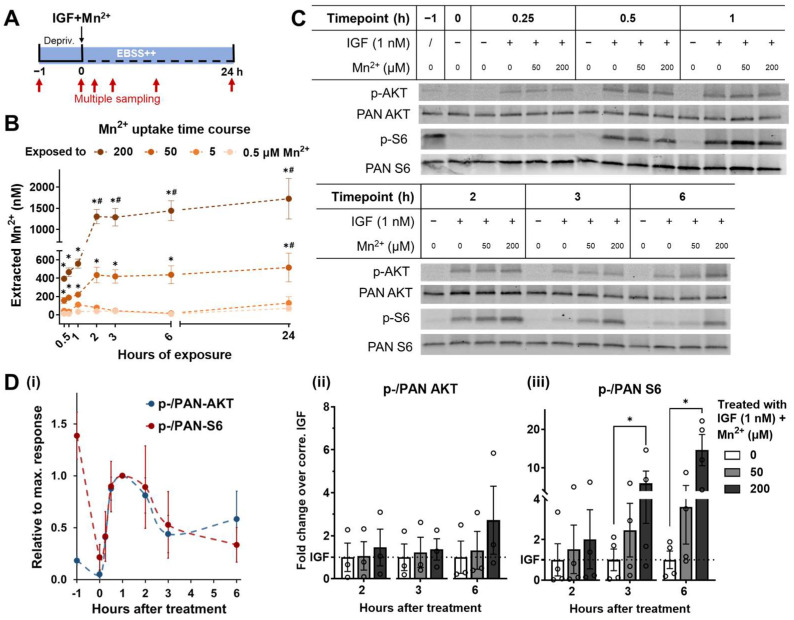
Exposure to Mn^2+^ prolongs AKT and S6 responses to IGF stimulation. (**A**) Schematic plot of experiment design. ST*Hdh*^Q7/Q7^ cells went through a 1 h serum deprivation in EBSS++ before receiving 0–200 μM Mn^2+^ with 1 nM IGF. Cell lysate collections and cellular Fura-2 manganese uptake assay (CFMEA) were performed before the serum deprivation and 0, 0.25, 0.5, 1, 2, 3, and 6 h after the start of the treatments as indicated by the red arrows. Depriv. = serum deprivation. EBSS++ = Earle’s Balanced Salt Solution (EBSS) supplied with 1× Glutamax, 1× non-essential amino acids, 14 mM HEPES, and 1× EAA. (**B**) Time course of intracellular Mn^2+^ concentration measured by CFMEA. Data points are plotted as mean ± SEM. Two-way ANOVA; exposure time–Mn^2+^ concentration interaction = *F* (15, 66) = 6.999; *p* < 0.0001; exposure time = *F* (5, 66) = 12.12; *p* < 0.0001; Mn^2+^ concentration = *F* (3, 66) = 133.1; *p* < 0.01. * *p* < 0.05 compared to cells exposed to 0.5 μM Mn^2+^ for the same time length. # *p* < 0.05 compared to cells exposed to the same concentration of Mn^2+^ for 0.25 h by Dunnett’s multiple comparisons test with individual variances computed for each comparison. (**C**) Representative western blots of p-AKT (Ser473), PAN AKT, p-S6 (Ser235/236), and PAN S6. Plus or minus signs in the table indicate whether the cells were treated with (+) or without (−) specific ingredient. (**D**) Quantification of p-/PAN AKT and p-/PAN S6 ratios. p-/PAN AKT N = 3; p-/PAN S6 N = 4. (**i**) Time course of IGF-only induced p-/PAN AKT and p-/PAN S6 ratio changes over time. Data points are plotted as mean ± SEM relative to the 1 h maximum response. (**ii**,**iii**) p-PAN AKT and p-/PAN S6 ratios, respectively, when simultaneously treated with Mn^2+^ (0, 50, or 200 μM) and 1 nM IGF for 2, 3, and 6 h. Data are displayed in mean ± SEM normalized to non-deprived vehicles and standardized to non-exposed groups at each time point. Ordinary one-way ANOVA was separately performed within each time point, and only the analysis with a significant difference detected was listed. S6 at 3 h: treatment = *F* (2, 9) = 1.614; *p* = 0.2518. S6 at 6 h: treatment = *F* (2, 9) = 7.484; *p* = 0.0106. * *p* < 0.05 compared to no Mn^2+^ control by Dunnett’s multiple comparisons test.

In the experiments described above, AKT and S6 activation were assessed at a single time point, 3 h after treatment of Mn^2+^ combined with IGF. Considering that this western blot analysis creates a snapshot of intracellular kinase activities, temporal/time course factors may provide additional interpretations of the results. Since S6 lies downstream of AKT in the canonical signal transduction pathway, the observed differential magnitude of responses could theoretically reflect different temporal kinetics of peak activity, with, plausibly, S6 being delayed from AKT in the cascade. Therefore, we performed a time course experiment by sampling cell lysates at serial time points, starting from just before the serum deprivation through to 6 h after the insulin/IGF treatment initiation. In agreement with the prior evaluations of the temporal pattern of IIS in cultured cell models, Q7 cells respond to 1 nM IGF stimulation within 30 min, represented by increased phosphorylation in both AKT and S6 [34]. The AKT and S6 activation stimulated by IGF peaked around 1 h, and both signals receded and were flattened after a period of 6 h following treatment initiation (Figure 3D(i)).

By adding 50 and 200 μM Mn^2+^ upon the IGF treatment, we found that the responses of AKT and S6 to the stimulation were prolonged (Figure 3D(ii,iii)). AKT phosphorylation did not show significant deviation from the IGF-only treatment when Mn^2+^ was added for 2 or 3 h; while its activity showed an increasing trend, triggered by 200 μM Mn^2+^ with IGF for 6 h (Figure 3D(ii), N = 3). Similarly, S6 activity under 200 μM Mn^2+^ + 1 nM IGF was notably elevated from single IGF stimuli for 6 h with a higher fold-change compared to the 2 and 3 h increase (Figure 3D(iii), N = 3). Note, however, that the effect size we detected here was smaller than seen in Figure 1, with AKT changes not reaching statistical significance. We suspect this failure in showing statistical significance is due to a lower number of biological replicates and a relatively larger variance across the replicates. Despite the smaller effect size, it is noteworthy that, by 6 h after treatment, the S6 activity displayed significant differences compared with vs. without Mn^2+^, which was not observed for AKT. The S6 phosphorylated/PAN ratio started descending after 2 h of IGF-only treatment (crimson line in Figure 3D(i)), while with Mn^2+^ added, the ratio showed an upward shift (Figure 3D(iii)). Further, the 200 μM Mn^2+^ + IGF significantly boosted S6’s activity 3 and 6 h after the treatment, and 50 μM Mn^2+^ + IGF also moderately elevated S6’s phosphorylation level (Figure 3D(iii)). These results again agree with our identified synergistic effect of Mn^2+^ + IGF co-treatment on IIS [21] and further indicate an alteration of Mn^2+^ on the cellular response pattern to regular IR/IGFR stimulation.

In parallel, we conducted a cellular Mn^2+^ uptake time course via our CFMEA method through 0–24 h of Mn^2+^ with the IGF treatment (Figure 3B). It was demonstrated that Mn^2+^ at 50 and 200 μM rapidly increased intracellular Mn^2+^ content in Q7 cells, with a significant deviation from the control group being detectable as early as 15 min past the metal ion addition. With lower concentrations of 0.5 and 5 μM, intracellular Mn^2+^, measured by CFMEA, peaked at 1 h and accumulated to a higher but not statistically significant total amount of Mn^2+^ compared to the vehicle 24 h after. With the higher concentrations of 50 and 200 μM, the Mn^2+^ uptake continued through 0–6 h and reached a plateau with a significantly higher extracted Mn^2+^ concentration after 24 h.

Combining the time course of both the signaling response and the total intracellular Mn^2+^ uptake, we postulated that the acute co-treatment of Mn^2+^ and IGF for 3 h may not fully represent the cellular responses to Mn^2+^ in the presence of biological fluctuations of IIS signaling that occur with metabolic changes over time, as there was a discrepancy in the timeline of the rapid-increasing phase between the signaling response and Mn^2+^ uptake. Specifically, the peak of AKT and the S6 response to insulin/IGF signaling at 1 h occurs prior to the peak of intracellular Mn^2+^ at around 2 h. Therefore, we sought to evaluate the Mn^2+^-dependent kinase activities following the establishment of a steady state of elevated intracellular Mn^2+^ by using a 24 h pre-treatment with Mn^2+^ to reach a steady state before IIS stimulation.

### 3.4. Higher Responsiveness of S6 Is Recapitulated under a Steady-State Increase of Intracellular Mn^2+^

**Figure 4:**
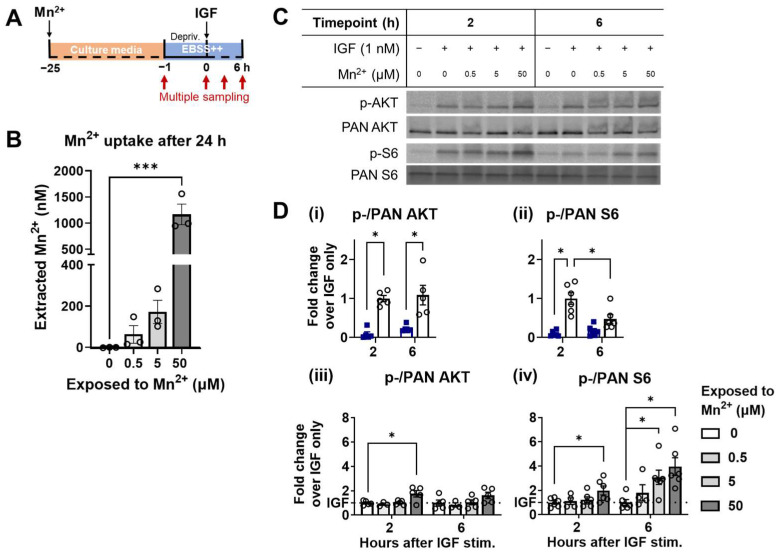
A 24 h pre-exposure to Mn^2+^ significantly elevated intracellular Mn^2+^ and extended S6 response to physiological insulin-like growth factor (IGF) stimulation. (**A**) Schematic plot of experiment design where the red arrows indicate the sample collection timepoints. ST*Hdh*^Q7/Q7^ cells went through a 24 h pre-exposure with relatively low Mn^2+^ concentrations ranging from 0, 0.5, 5, and 50 μM. Following the pre-exposure, cells were subject to a 1 h serum deprivation in EBSS++ before receiving an IGF hit at 1 nM. Mn^2+^ exposures were continued during the serum deprivation and IGF hit. Cell lysate collections and cellular Fura-2 manganese uptake assay (CFMEA) were performed before the serum deprivation and 0, 2, and 6 h after the start of the treatments. Depriv. = serum deprivation. EBSS++ = Earle’s Balanced Salt Solution (EBSS) supplied with 1× Glutamax, 1× non-essential amino acids, 14 mM HEPES, and 1× EAA. (**B**) Intracellular Mn^2+^ measured by CFMEA following 24 h of indicated exposure. Bars plotted in mean ± SEM, N = 3. Ordinary one-way ANOVA; treatment = *F* (3, 8) = 27.28; *p* = 0.0001. *** *p* = 0.0001 compared to the non-exposed group by Dunnett’s multiple comparisons test. (**C**) Representative western blots of p-AKT (Ser473), PAN AKT, p-S6 (Ser235/236), and PAN S6. The table above the blots shows the treatment group, where plus and minus signs indicate whether the cells were treated with (+) or without (−) specific ingredient. (**D**(**i,ii**)) Quantification of p-/PAN AKT (**i**) and p-/PNA S6 (**ii**) ratios comparing vehicle group maintained in EBSS++ vs. IGF-only treatment group. Data were normalized to non-deprived vehicles and displayed as mean ± SEM. (**i**) p-/PAN AKT: N = 5. Two-way ANOVA; IGF = *F* (1, 8) = 36.06; *p* = 0.0003; treatment time = *F* (1, 8) = 0.8710; *p* = 0.3780; IGF-treatment time interaction = *F* (1, 8) = 0.04643; *p* = 0.8348. * *p* < 0.05 in indicated comparison by RM two-way ANOVA with matched values by different time points, followed by Bonferroni’s multiple comparisons test. (**ii**) p-/PAN S6: N = 6. Two-way ANOVA; IGF = *F* (1, 10) = 52.17; *p* < 0.0001; treatment time = *F* (1, 10) = 3.967; *p* = 0.0744; IGF-treatment time interaction = *F* (1, 10) = 7.154; *p* = 0.0233. * *p* < 0.05 in indicated comparison by RM two-way ANOVA with matched values by different time points, followed by Bonferroni’s multiple comparisons test. (**iii**,**iv**) Quantification of p-/PAN AKT (**iii**) and p-/PAN S6 (**iv**) ratios comparing Mn^2+^-exposed group to the non-exposed group after 2 and 6 h after the initiation of the treatment. Data were normalized to non-deprived vehicles and standardized to non-exposed groups at each time point. Ordinary one-way ANOVA was separately performed within each time point. AKT at 2 h: treatment = *F* (3, 14) = 6.180; *p* = 0.0068. AKT at 6 h: treatment = *F* (3, 14) = 2.158; *p* = 0.1386. S6 at 2 h: treatment = *F* (3, 18) = 2.918; *p* = 0.0623. S6 at 6 h: treatment = *F* (3, 18) = 5.433; *p* = 0.0077. * *p* < 0.05 in indicated comparison by Dunnett’s multiple comparisons test.

As shown in Figure 3B, the intracellular Mn^2+^ content reached a plateau around 2 h after the initiation of the exposure and maintained these levels for 24 h. Therefore, to achieve a steady state of intracellular Mn^2+^ in the Q7 cells, a pre-exposure of 24 h Mn^2+^ in a cultured medium was introduced. Following the pre-exposure, the Mn^2+^ uptake was first measured by CFMEA (Figure 4B), indicating a significant increase of intracellular Mn^2+^ in the cells exposed to 50 μM. Despite no statistical significance in the groups exposed to 0.5 and 5 μM Mn^2+^, a trend of deviation from the control was detected (69.84 ± 32.48 nM; 128.23 ± 68.07 nM extracted Mn^2+^, respectively), as the low-level increase in intracellular Mn^2+^ is near the detection threshold for these very low Mn^2+^-exposure concentrations. Following the Mn^2+^ pre-exposure, a 1-h serum deprivation followed by IGF treatment was applied with continued Mn^2+^ dosing. Responsiveness to IIS stimulation was first confirmed, represented by significantly increased AKT and S6 phosphorylation, which was induced by 2 h of IGF alone treatment (Figure 4D(i,ii)). In the meantime, continuous exposure to 50 μM Mn^2+^ induced a significant magnification of IGF/insulin-dependent AKT and S6 phosphorylation, with a ~2-fold increase by the IGF-only group after 2 h of IGF treatment. When the IGF treatment was extended to 6 h, i.e., a Mn^2+^ overload maintained for 30 h, the activity of AKT remained at a similar level as that of 2 h after IGF (Figure 4D(iii)). While AKT’s phosphorylation level was not significantly affected by the duration of either the Mn^2+^ exposure or IGF stimulation, the S6 response to Mn^2+^ + IGF was exaggerated over time. This exaggeration was seen as a notable increase in the p-/PAN S6 ratio in the 5 and 50 μM Mn^2+^-exposed groups compared to the IGF-only treated group (Figure 4D(iv)). This elevation of S6 phosphorylation in the presence of low-level Mn^2+^ by IIS stimulation suggests that the upregulation observed in the synchronous treatment of Mn^2+^ and IGF is not only the result of an acute cellular Mn^2+^ uptake response initiated by extracellular environmental changes but is indeed an alteration induced by Mn^2+^ overload in the kinetics of IIS activation. These results also further emphasize the specificity and sensitivity of S6 phosphorylation in response to threshold-level elevations of intracellular Mn^2+^ concentrations.

## 4. Discussion

Mn^2+^-induced perturbations in the IIS pathway have been reported in cultured neuronal and animal models, with direct interactions revealed by our lab [18,20,21,25,35]. Bryan et al. (2020) first reported that exposure to toxic concentrations of Mn^2+^ potentiates the IIS response to a physiological level of IGF. Here, we sought to further investigate the specificity and sensitivity of the IIS pathway as a highly Mn^2+^-preferred pathway under a low-level Mn^2+^ overload.

First, at the sub-cytotoxic levels, the Mn^2+^ concentration dependently potentiated both the AKT and S6 responses to IIS stimulation, with S6 showing a higher sensitivity to lower Mn^2+^ concentrations (Figure 1). Consistent with prior publications, our results showed that when Mn^2+^ was present at toxic levels of concentrations of 50 μM and above, it induced a significant synergistic effect with IIS stimulation on both AKT and S6 phosphorylation [21]. However, at the Mn^2+^ concentration of 5 μM, where AKT activity was not detectably altered, S6’s phosphorylation was significantly potentiated. This pattern is counterintuitive, given that AKT lies upstream of S6 and serves as a critical input for S6 activation. Furthermore, the magnitude of S6 phosphorylation induced by low-level Mn^2+^ with IGF is greater than that for AKT phosphorylation. The negative feedback of S6 on AKT activity could potentially be contributed to its lack of response [36], as we presume that the intermediate regulator in the IIS pathway and the direct activator of S6, mTORC1, is the key respondent for S6 sensitivity to low Mn^2+^ concentrations. Recent studies by others have demonstrated that mTORC1 activity can be increased by physiological levels of Mn^2+^ and that Mn^2+^, compared to another common metallic enzyme co-factor Mg^2+^, is a preferred co-factor for mTORC1 [6]. This preference for Mn^2+^ suggests that mTORC1 activity may be more sensitive to intracellular Mn^2+^ concentration fluctuations, as it utilizes Mn^2+^ for normal functioning. Furthermore, in prior in vivo low-level Mn^2+^ exposure studies, mTORC1 hyperactivation was observed under Mn^2+^ overload, which led to a moderate elevation of brain Mn^2+^ content with no apoptosis [35,37,38], providing additional evidence for its sensitivity to excess low Mn^2+^ concentrations.

The involvement of mTORC1 is also supported by the results shown in Figure 2, where IGF receptor inhibitors and EAA deprivation both suppressed the S6 response to Mn^2+^ + IGF. The application of IGFR inhibitors completely abrogated the elevation of both AKT and S6 phosphorylation (Figure 2A), indicating that the signaling effects of Mn^2+^ require the direct involvement of IGFR, which is consistent with our prior reported interactions of Mn^2+^ with IGFR at higher concentrations [21]. Moreover, by testing the inhibition of IGFR with multiple pharmacological antagonists, we minimized the potential for the different off-target effects of each individual inhibitor to contribute to this observation, thus validating the essentiality of the IIS pathway, specifically in the induction of S6 phosphorylation by threshold-level increases of Mn^2+^ concentrations. On the other hand, depriving the system with EAA blocked the Mn^2+^-dependent IIS activation of S6 but not AKT (Figure 2B), implicating that mTORC1, the main sensor for cellular amino acid supplementation lying downstream of AKT, is responsible for the sensitivity of S6 to low-level threshold increases in extracellular Mn^2+^ overload.

In terms of possible explanations for the significantly increased sensitivity of S6 phosphorylation in low-level Mn^2+^ exposure, another potential contributor may be the cross-talk between IIS and other S6-signaling input resources. A nutrient-dependent pathway that cooperates with IGF signaling—the cyclic adenosine monophosphate (cAMP)/protein kinase A (PKA) pathway—acts specifically on the S6 Ser235/236 sites and has been readily reported to be activated in acute Mn^2+^-induced neurotoxicity [39,40]. In dopamine D1 receptor (D1R)-expressing striatal neurons, cAMP/PKA-induced phosphorylation of S6 Ser235/236 results from the specific phosphorylation of DARPP32 (dopamine- and cAMP-regulated phosphoprotein of molecular weight 32,000) [41]. DARPP32 is a protein phosphatase inhibitor highly expressed in medium-sized spiny neurons in the neostriatum, which interact with activated mTORC1, as well [42,43,44]. Q7 cells originating from mouse striatum display high expression levels of both D1R and DARPP32 [45,46]. This medium-sized spiny neuronal phenotype has been associated with the hypersensitivity of Q7 cells to endoplasmic reticulum (ER) stress; thus, it may also explain their hyper-vulnerability to Mn^2+^ overload [14,46].

S6 may be specifically sensitive to threshold-level Mn^2+^ overload concentrations compared to other kinases in the mTOR regulatory system. The hypersensitivity of S6 to low-level Mn^2+^ overload was confirmed in the 24-h pre-treatment, as shown in Figure 4, where only the S6 phosphorylation was significantly elevated by IGF stimulation under exposure to 5 μM Mn^2+^. However, despite the notable elevation in S6 phosphorylation, we failed to detect significant phosphorylation at either the mTORC1 Ser2448 site or the other mTORC1 target, 4E-BP1, at the Thr37/46 site with pre-loaded 5 μM Mn^2+^ for 24 h (Supplemental Figure S1). The mTORC1 Ser2448 site is located on the FIT domain (Found in TOR) spanning residues, and its phosphorylation is recognized as an indirect outcome of AKT input on mTORC1. In the meantime, Ser2448 phosphorylation serves as a part of the mTOR/p70S6k feedback loop [47,48]. It is yet unclear whether the activation of p70S6k provides a positive or negative regulation on Ser2448 phosphorylation [49]. The complexity of the mTOR/p70S6k loop could explain the lack of Ser2448 phosphorylation, despite the AKT activation that was observed. In another Mn^2+^ neurotoxicity study by Zhang et al., mTORC1 Ser2448 phosphorylation was also undetectable, and the application of its inhibitor rapamycin restored downstream autophagy flux and rescued toxicity phenotypes in astrocytes [50]. This thus supports an argument that Ser2448 phosphorylation does not fully represent the involvement of mTORC1 in cellular responses to toxicants [50]. The other target downstream of mTORC1, 4E-BP1, also failed to display significant changes in its phosphorylation level at the Thr37/46 site in our 24-h sub-cytotoxic Mn^2+^ exposure. 4E-BP1 is a member of the translation repressor protein family and is a broadly accepted mTORC1 substrate in parallel with S6. Upon activation by mTORC1, hyperphosphorylated 4E-BP1 dissociates from eukaryotic translation initiation factor 4E (eIF4E) and recruits eIF4G to the mRNA 5′-cap, thereby initiating cap-dependent translation and enhancing the downstream cell cycle and proliferation [51]. Although 4E-BP1 is a well-described target of activated mTORC1, multiple studies have reported weaker and preemptive 4E-BP1 phosphorylation at Thr37 compared to S6 with the same level of stimulus [52,53]. With the 50 μM Mn^2+^ exposure, we observed a trend yet no significant decrease in the phosphorylation level of 4E-BP1, which correlates with the decreased cell proliferation rate we reported on 24-h Mn^2+^ exposure with the ST*Hdh* model [12]. Therefore, future studies should more comprehensively characterize the mTOC1/4E-BP1 axis as a potential mechanism that underlies the cytostatic effects of low-level Mn^2+^ overload.

The time courses established in Figure 3 and Figure 4 showed that IIS-induced kinase phosphorylation was prolonged by exposures to Mn^2+^. AKT and S6 phosphorylation were first brought to the baseline level by 1-h serum deprivation, which, on the one hand, enables further manipulation and, on the other, reflects the fluctuations of brain-IGF levels. By adding physiological levels of IGF to the deprived system, the kinetics of AKT and S6 responses aligned with each other, as both were initiated within 30 min, peaked at 1 h, and were dampened by 6 h (Figure 3B,D), which is also corroborative of the previously reported experiments [54,55,56,57]. In contrast with the oscillatory signaling transduction under physiological stimulation, AKT and S6 in cells exposed to Mn^2+^ maintained a higher amplitude until 6 h after treatment (Figure 3D and Figure 4C). Furthermore, the simultaneous treatment of Mn^2+^ with IGF for 2 h only induced synergistic activation in S6, while an established steady state of elevated intracellular Mn^2+^ (Figure 4B, when exposed to 50 μM) triggered an increase in phosphorylation of both AKT and S6. Overactivation of mTORC1/S6 has been reported to be responsible for aberrant protein synthesis, abnormal cell cycle, as well as synaptic transmission failures, which are directly relevant to neurodegenerative disease pathogenesis [58,59,60].

Beyond investigating the time-dependent response of IIS signaling, we also tested the kinetics of intracellular Mn^2+^ accumulation in Q7s (Figure 3B and Figure 4B). As an in vitro model, the Q7 cell line expresses known Mn^2+^ uptake transporters, divalent metal transporter (DMT1) and transferrin, as well as the Mn^2+^ efflux modulator SLC30A10, and therefore, is a sufficient representation of Mn^2+^ maintenance mechanisms at the cellular level [29,61]. In the meantime, it should be taken into consideration that the in vitro models cannot fully imitate systematic regulations in vivo, such as interactions across various divalent metals and between neuronal and glial cells [62]. These crucial factors balance the whole-brain Mn^2+^ level between its essentiality vs. toxicity and yet add to the complexity of understanding the mechanisms of Mn^2+^ neurotoxicity.

The differential AKT and S6 activity changes in response to acute vs. steady-state Mn^2+^ overloads potentially indicate that Mn^2+^ exposure can alter the response pattern of striatal neurons to IGF. Long-term alteration of IIS and mTORC1/S6 activities can lead to limited dynamic ranges of the signaling, resulting in failures of neuronal transduction and cognitive functions in the long run [63,64]. In transgenic mice with persistent mTORC1/S6 activation, striatal dopamine signaling was suppressed and correlated with impairments in cortical and cognitive behavioral domains [65]. Furthermore, balancing mTOR signaling activity is crucial for autophagy sufficiency and cellular responsiveness to IIS stimulation, which then points toward the pathogenesis and risk management of later-life Alzheimer’s and Parkinson’s diseases [66,67,68]. Although it remains controversial, there is an established linkage between chronic exposure to Mn^2+^ and neurodegenerative disease development, therefore indicating a potential mechanistic role of elevated mTORC1/S6 signaling in Mn^2+^ neurotoxicity. On the other hand, suppressing the mTORC1 pathway can generally be protective in chronic neurodegeneration, and specifically in Mn^2+^ toxicity, rapamycin inhibition on mTORC1 rescued the mitochondrial function in exposed primary astrocytes [50,69]. Taken together, this evidence strongly suggested that low-level Mn^2+^ overload not only induced activity potentiation of mTORC1/S6 but also prolonged its response to extracellular IGF fluctuations.

In conclusion, we identified mTORC1/S6 as a highly specific and sensitive downstream respondent to acute subtoxicological-level Mn^2+^ exposure. As a crucial intersection of the IIS pathway that utilizes Mn^2+^ as a co-factor under normal conditions, interruption of mTORC1 signaling may be the leading mechanism of the transition of Mn^2+^ essentiality to neurotoxicity.

## Data Availability

Data is contained within the article and Supplementary Material.

## Supplementary Materials

The following supporting information can be downloaded at: https://www.mdpi.com/article/10.3390/biom13081229/s1, Figure S1: 24-h pre-exposure to low-level Mn exposure followed by IGF stimulation did not significantly affect phosphorylation of mTORC1 at Ser2448 or 4E-BP1 at Thr37/46; Table S1: List of antibodies used in this study; PDF file S1: Original Blots_IGF- mTORC1-S6 Signaling Is Potentiated and Prolonged by Acute Loading of Subtoxicological Manganese Ion.

## Abbreviations

Cellular Fura-2 manganese uptake assay (CFMEA); divalent manganese ion (Mn^2+^); eukaryotic translation initiation factor 4E (eIF4E)-binding protein 1 (4E-BP1); insulin-like growth factor (IGF); IGF/insulin signaling (IIS); mammalian target of rapamycin complex 1 (mTORC1); protein kinase B (AKT); phosphorylated AKT (p-AKT); total AKT (PAN AKT); ribosomal protein S6 (S6); phosphorylated S6 (p-S6); total S6 (PAN S6); ST*Hdh*^Q7/Q7^ (Q7).
