# Supplementary material for: IGF/mTORC1/S6 Signaling Is Potentiated and Prolonged by Acute Loading of Subtoxicological Manganese Ion
Source: Biomolecules. 2023 Aug 8;13(8):1229. doi: 10.3390/biom13081229 (PMC10452562; doi:10.3390/biom13081229)

Original Images for Blots/Gels

IGF/mTORC1/S6 Signaling Is Potentiated and Prolonged by Acute Loading of Subtoxicological Manganese Ion

Xueqi Tang[1], Rekha C Balachandran[1,2], Michael Aschner[3], and Aaron B Bowman[1*]

1School of Health Sciences, Purdue University, West Lafayette IN

2Exponent Inc, Alexandria VA

3Dept of Molecular Pharmacology, Albert Einstein College of Medicine, Bronx NY

* Corresponding author

I. Original blots/gels displayed in main article

Original blots displayed in Figure 1B

Figure 1

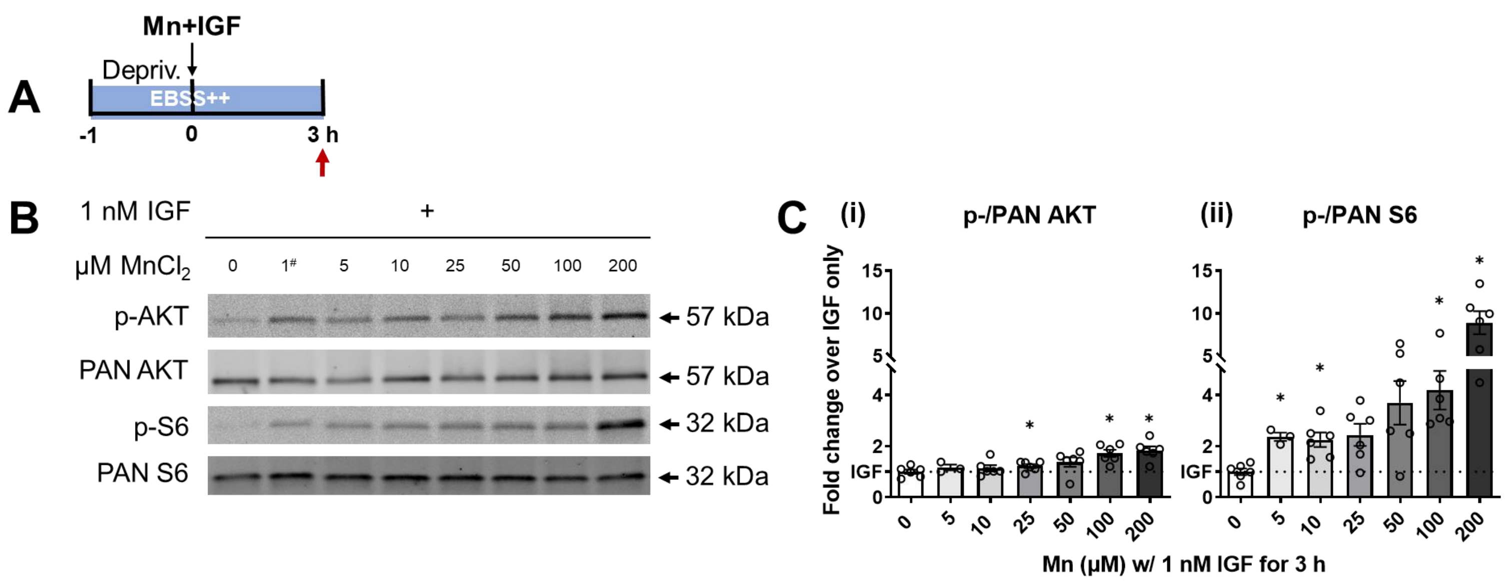

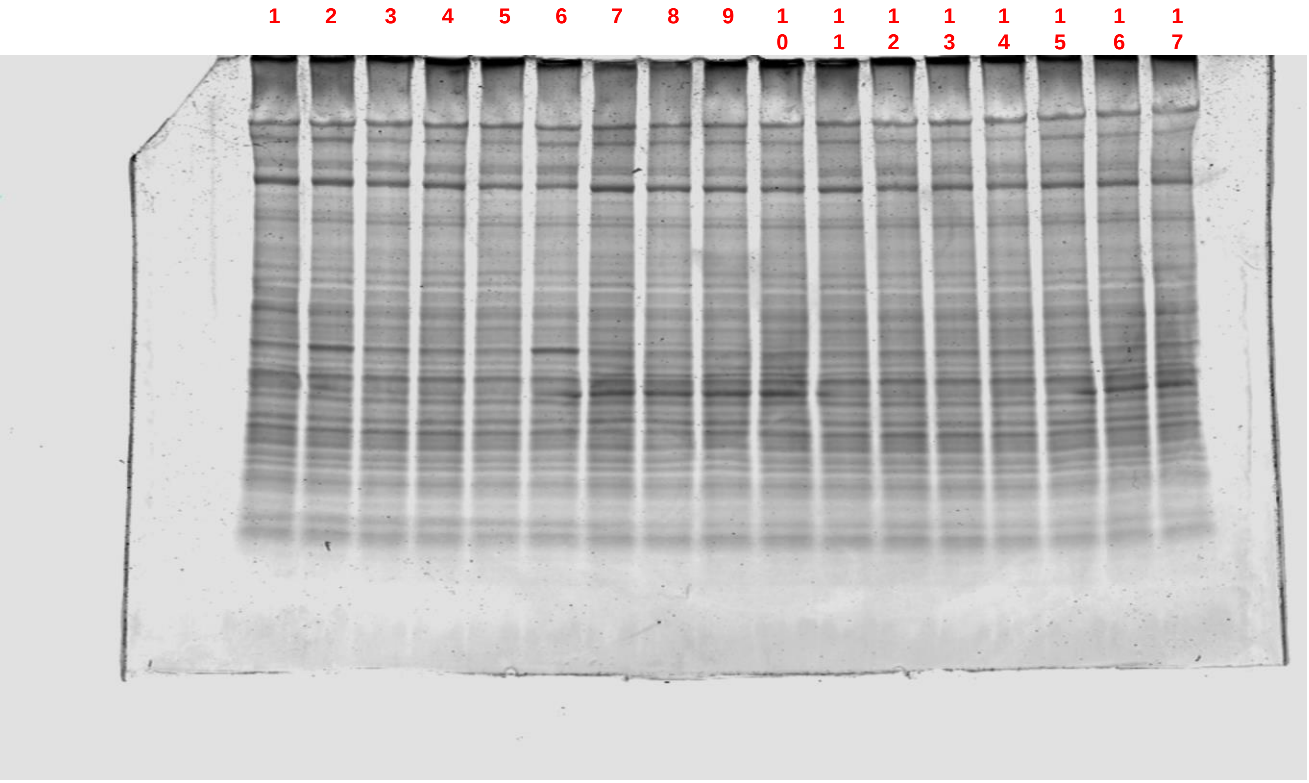

BR#	Lane#	1 nM IGF	Mn (μM)
1	1*	+	1
	2	+	10
	3	+	25
	4	+	50
	5	+	100
	6	+	200
2	7	-	0
	8	-	100
	9	-	200
	10	+	0
	11*	+	1
	12	+	5
	13	+	10
	14	+	25
	15	+	50
	16	+	100
	17	+	200

* used as loading control for normalization during quantification. BR = Biological Replicate

Sections displayed in Figure 1B were highlighted in red boxes.

p-AKT (Ser473)

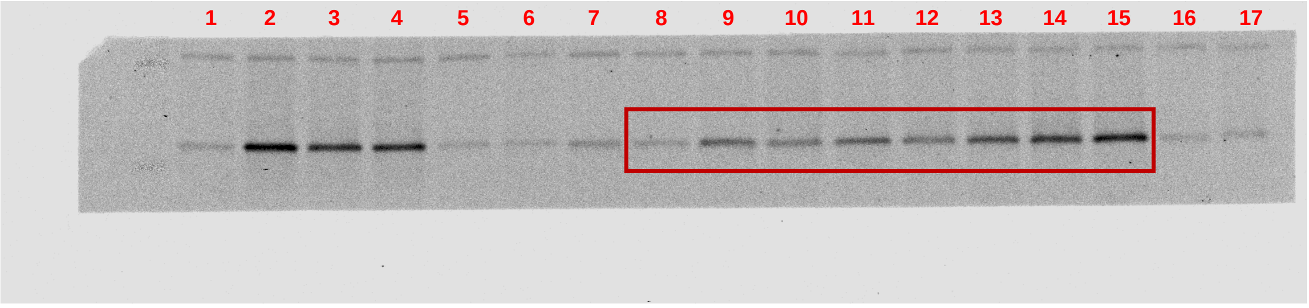

p-S6 (Ser235/236)

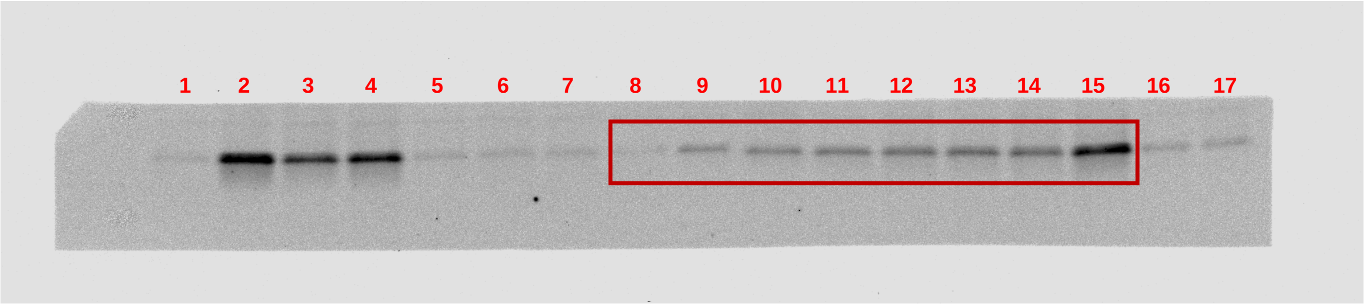

PAN-AKT

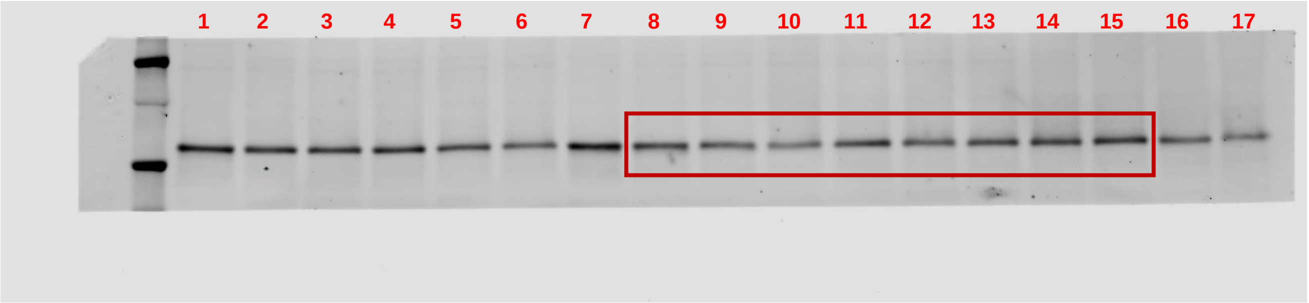

PAN-S6

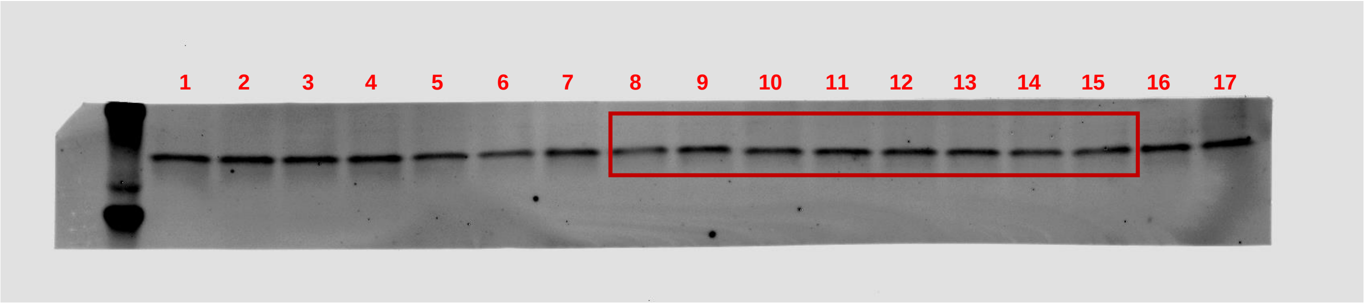

Original blot displayed in Figure 2A(ii)

Figure 2

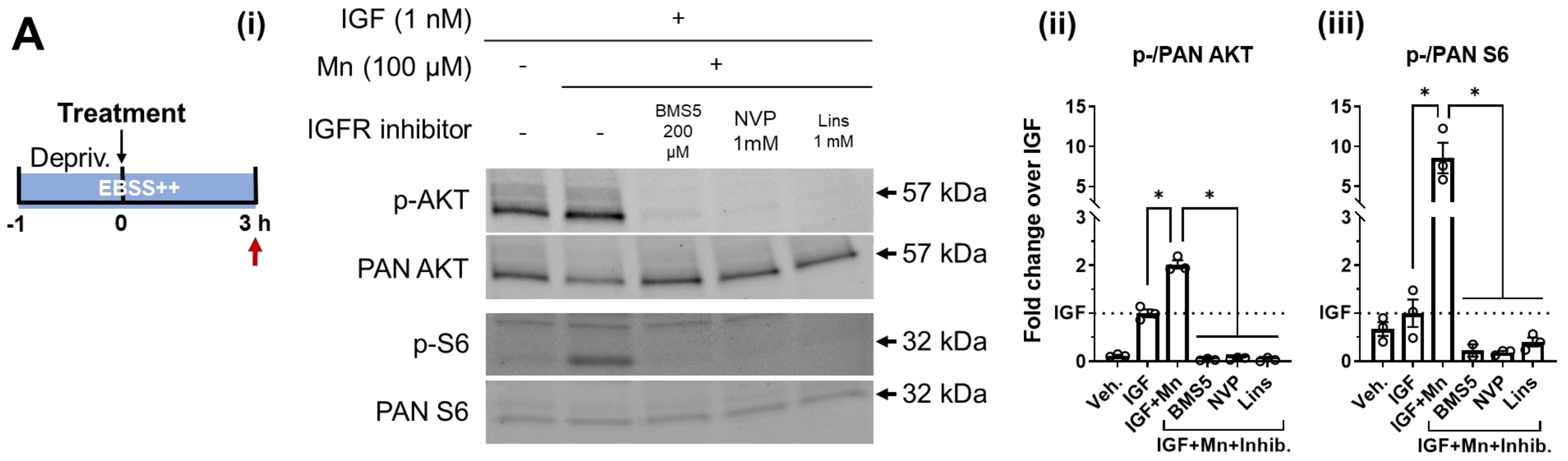

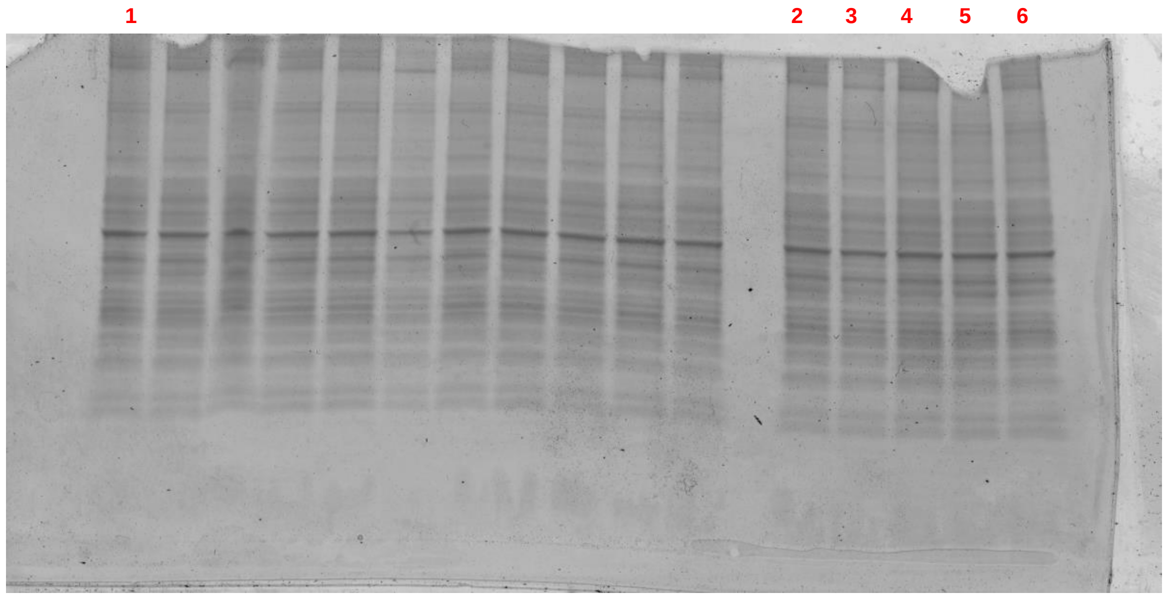

Lane#	1 nM IGF	Mn (µM)	IGFR inhibitor
1*	-	0	-
2	+	0	-
3	+	100	-
4	+	100	BMS5 (200 µM)
5	+	100	NVP (1mM)
6	+	100	Lins (1 mM)

* used as loading control for normalization during quantification
BMS5 = BMS-536924; NVP = NVP-AEW541; Lins = Linsitinib

Sections displayed in Figure 2A(ii) were highlighted in red boxes.
Only bands at molecular weight indicated by arrows represent targeted proteins.

p-AKT (Ser473)

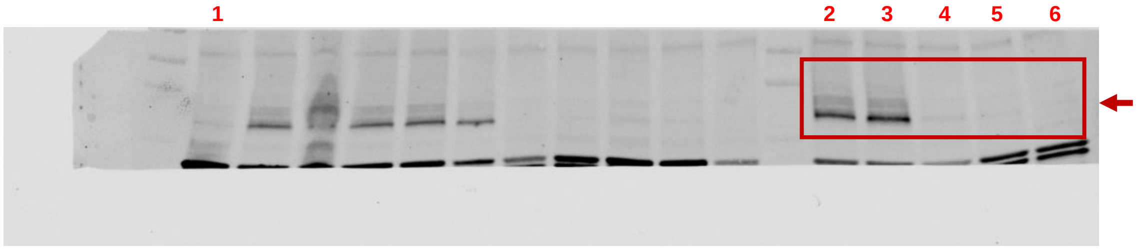

p-S6 (Ser235/236)

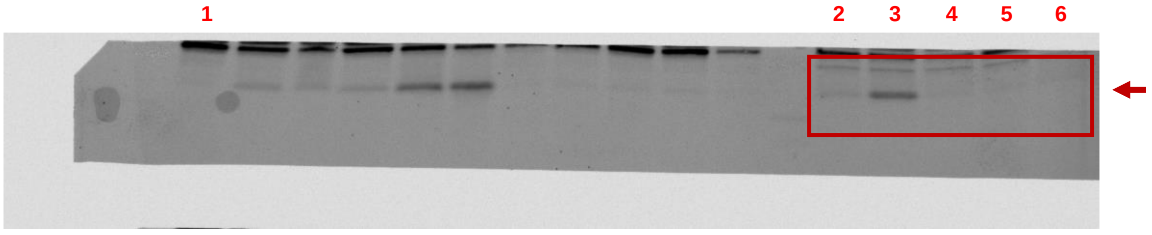

PAN-AKT

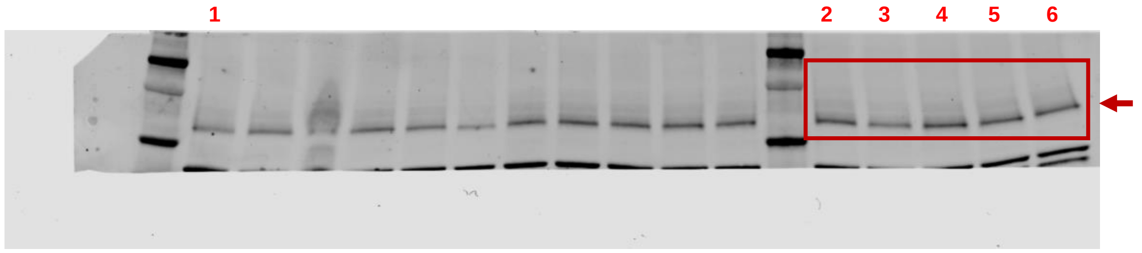

PAN-S6

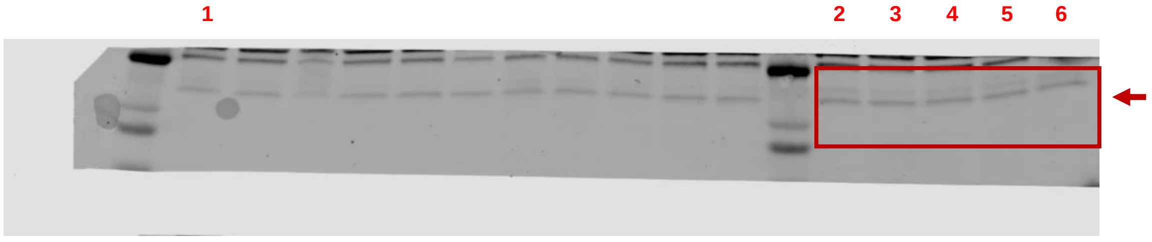

Original blots displayed in Figure 2B(ii)

Figure 2

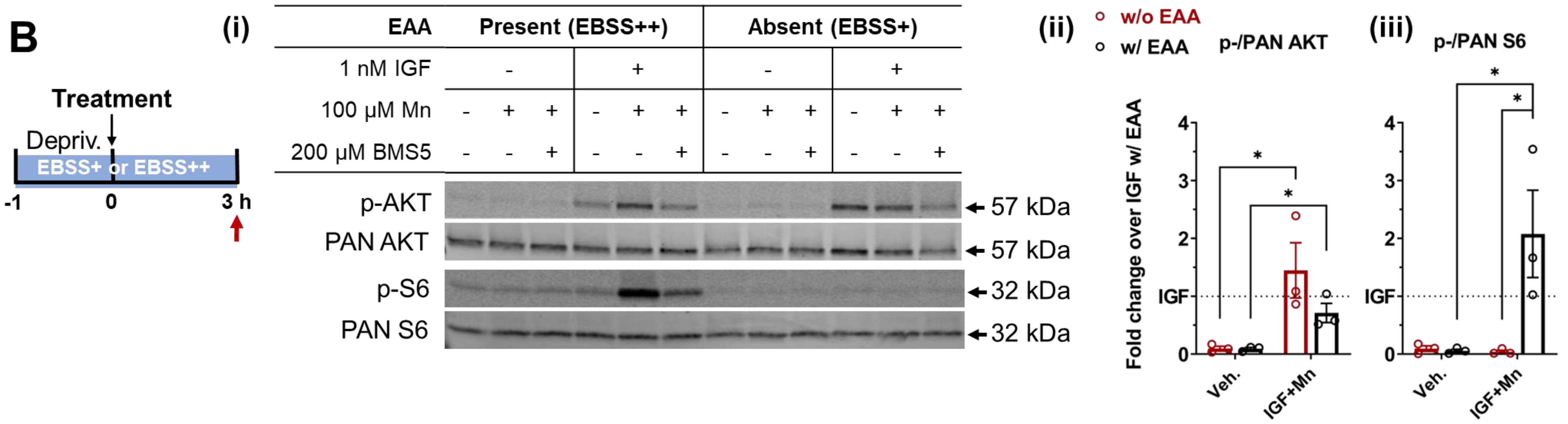

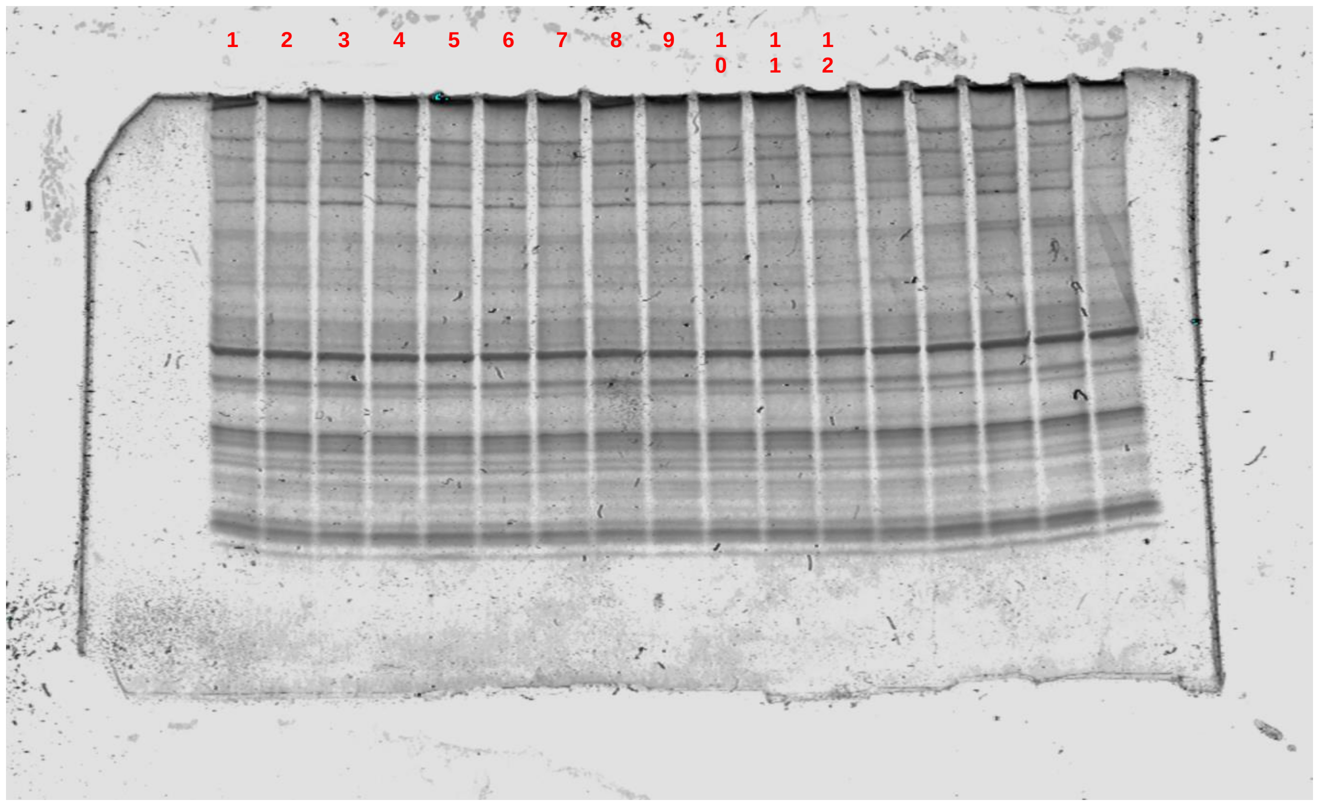

Lane#	Essential Amino Acids	1 nM IGF	100 µM Mn	200 µM BMS5
1	+	-	-	-
2			+	-
3			+	+
4		+	-	-
5			+	-
6*			+	+
7	-	-	-	-
8			+	-
9			+	+
10		+	-	-
11			+	-
12			+	+

* used as loading control for normalization during quantification.

Sections displayed in Figure 2B(ii) were highlighted in red boxes.

p-AKT (Ser473)

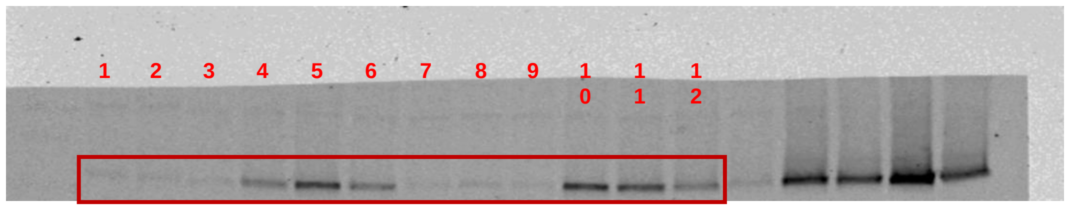

p-S6 (Ser235/236)

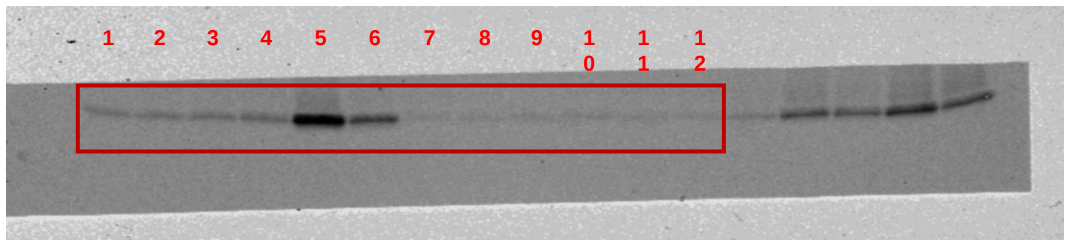

PAN-AKT

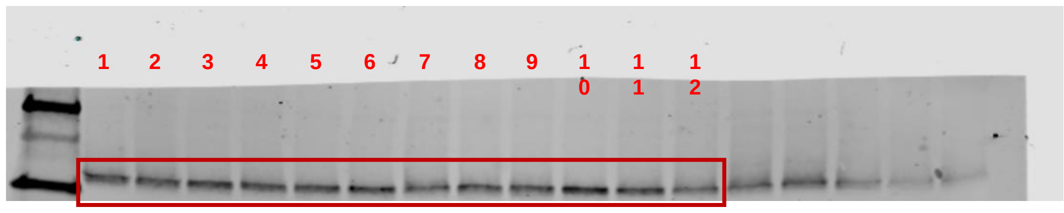

PAN-S6

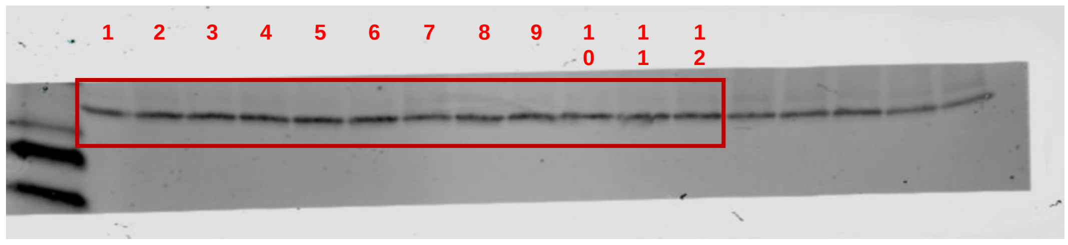

Original blots displayed in Figure 3C top

Figure 3

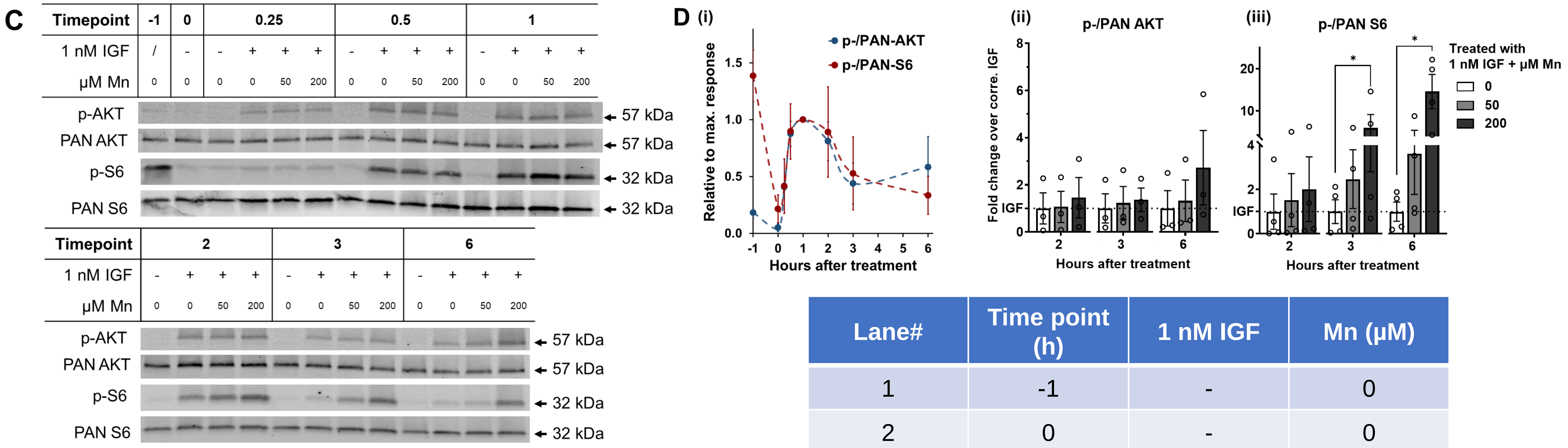

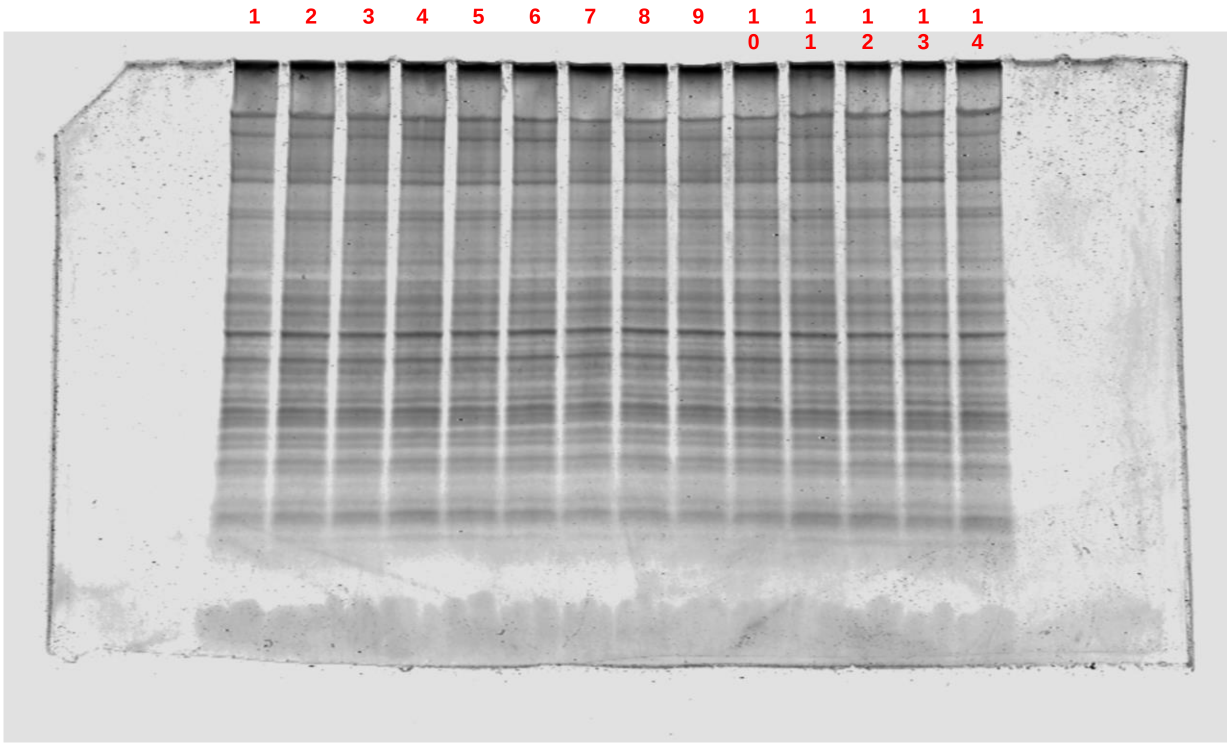

Lane#	Time point (h)	1 nM IGF	Mn (µM)
1	-1	-	0
2	0	-	0
3	0.25	-	0
4		+	0
5		+	50
6		+	200
7	0.5	-	0
8		+	0
9		+	50
10		+	200
11	1	-	0
12		+	0
13		+	50
14		+	200

Sections displayed in Figure 3C top were highlighted in red boxes.

p-AKT (Ser473)

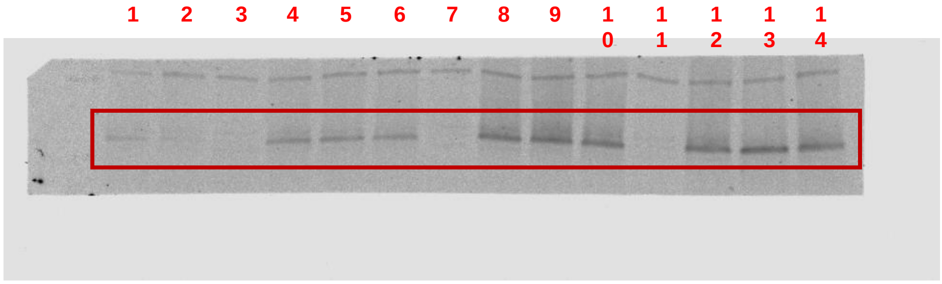

p-S6 (Ser235/236)

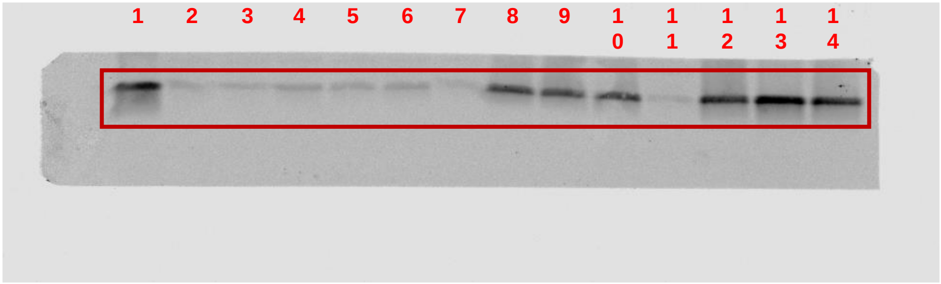

PAN-AKT

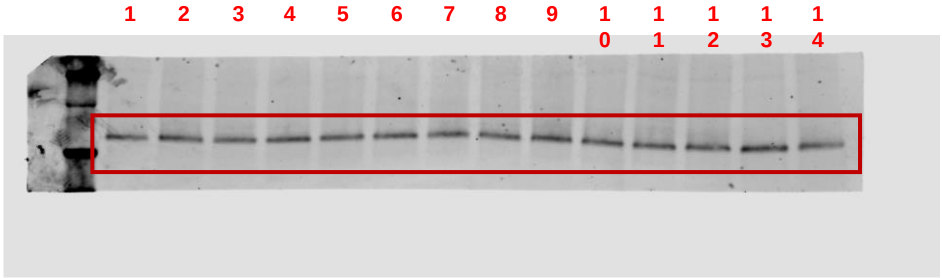

PAN-S6

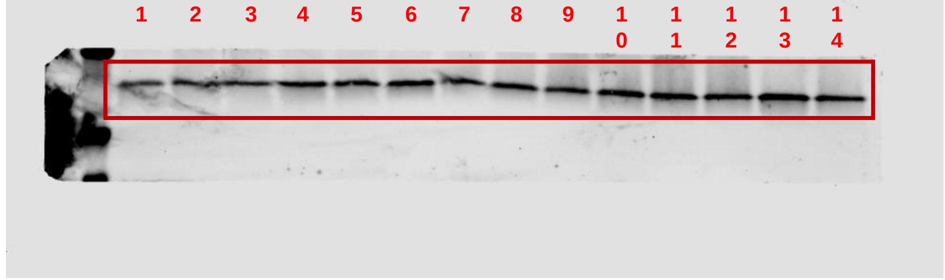

Original blots displayed in Figure 3C bottom

Figure 3

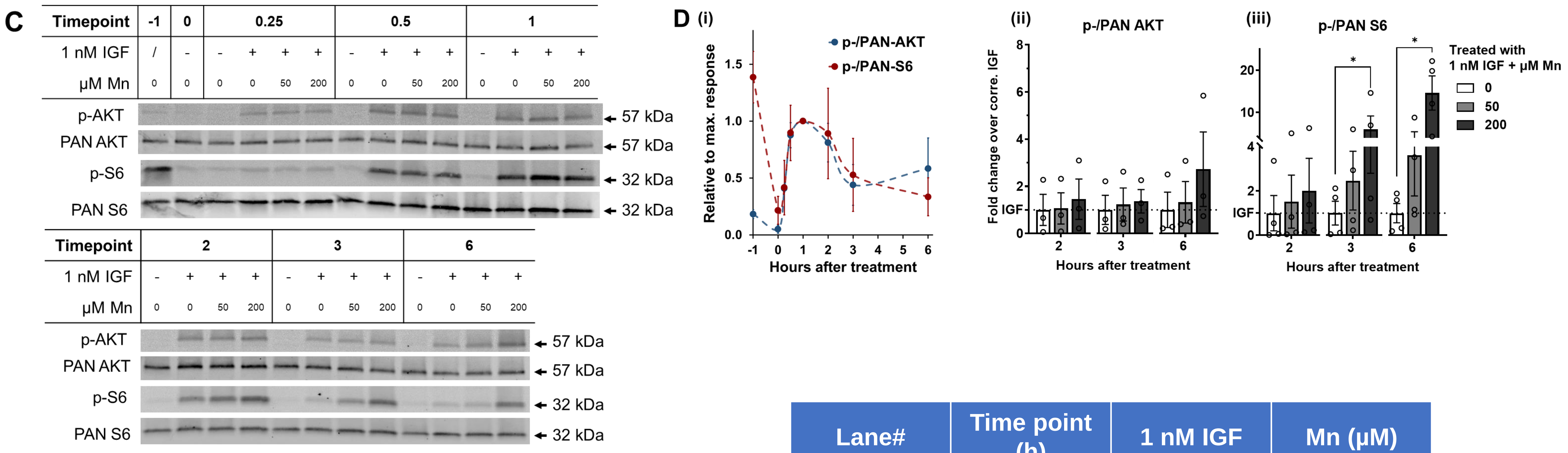

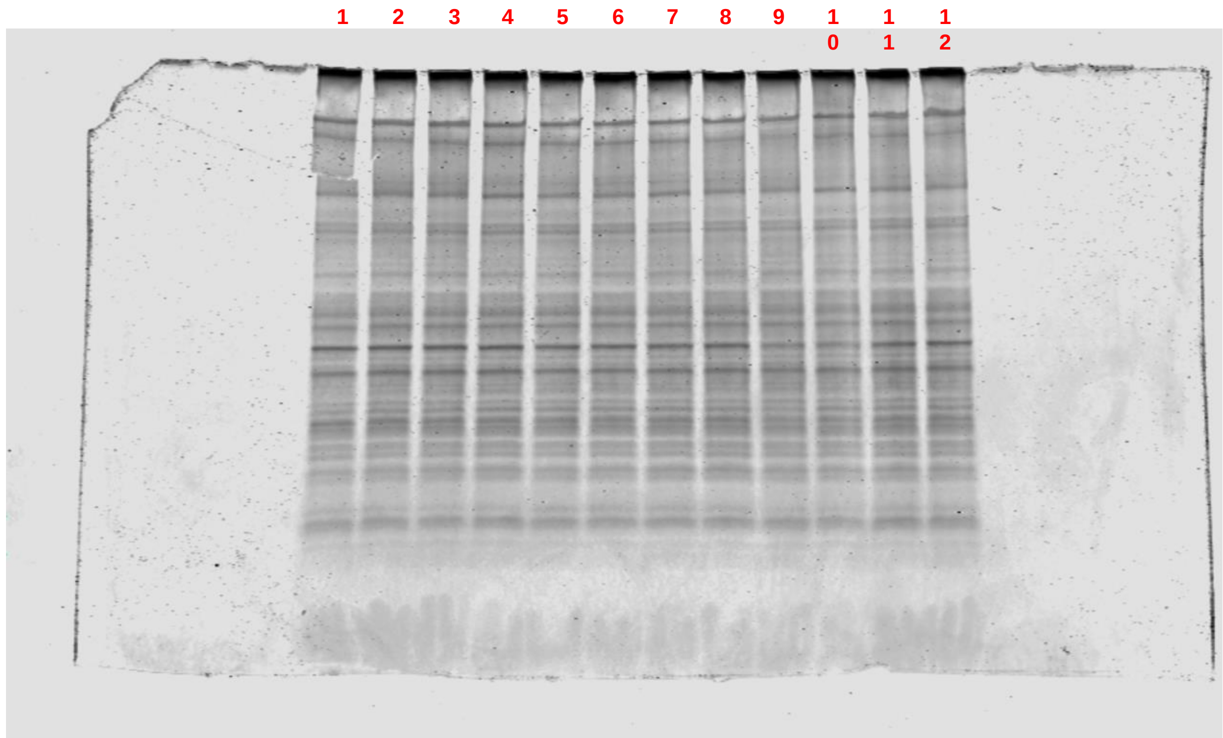

Lane#	Time point (h)	1 nM IGF	Mn (µM)
1	2	-	0
2		+	0
3		+	50
4		+	200
5	3	-	0
6		+	0
7		+	50
8		+	200
9	6	-	0
10		+	0
11		+	50
12		+	200

Sections displayed in Figure 3C bottom were highlighted in red boxes.

p-AKT (Ser473)

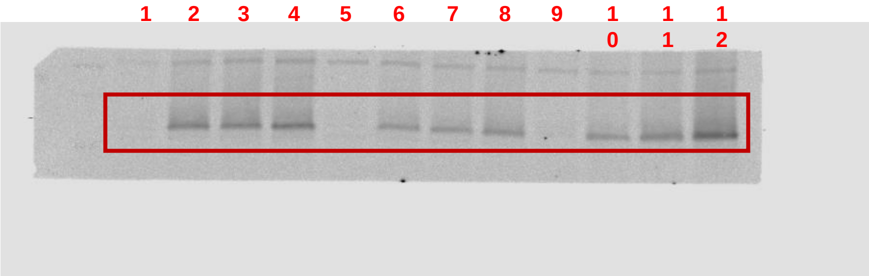

p-S6 (Ser235/236)

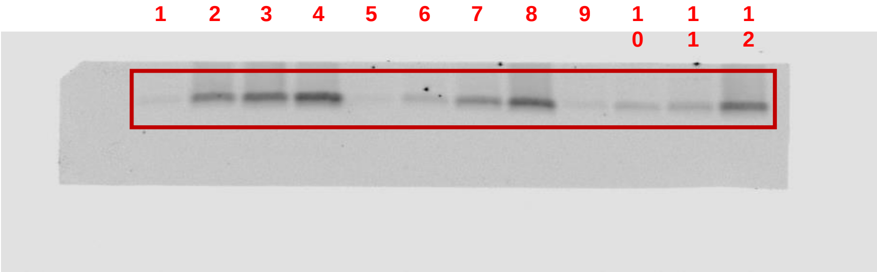

PAN-AKT

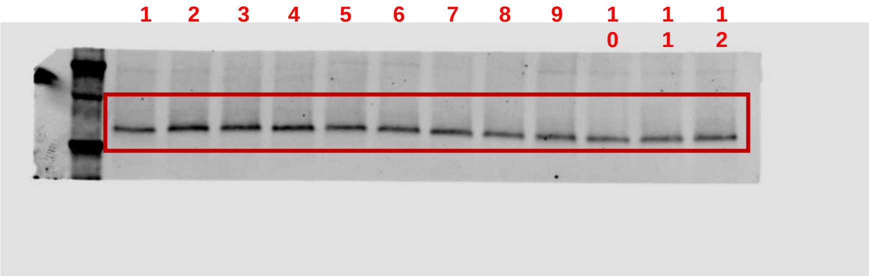

PAN-S6

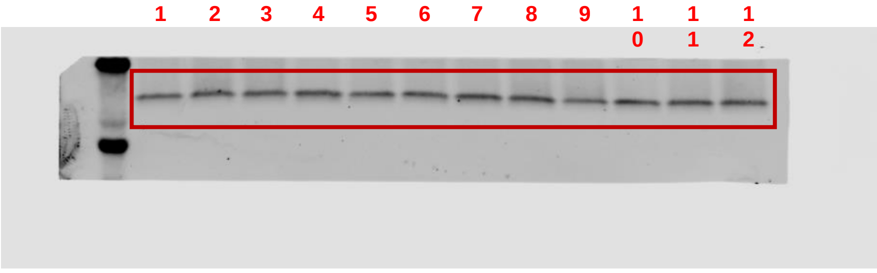

Original blots displayed in Figure 4C

Figure 4

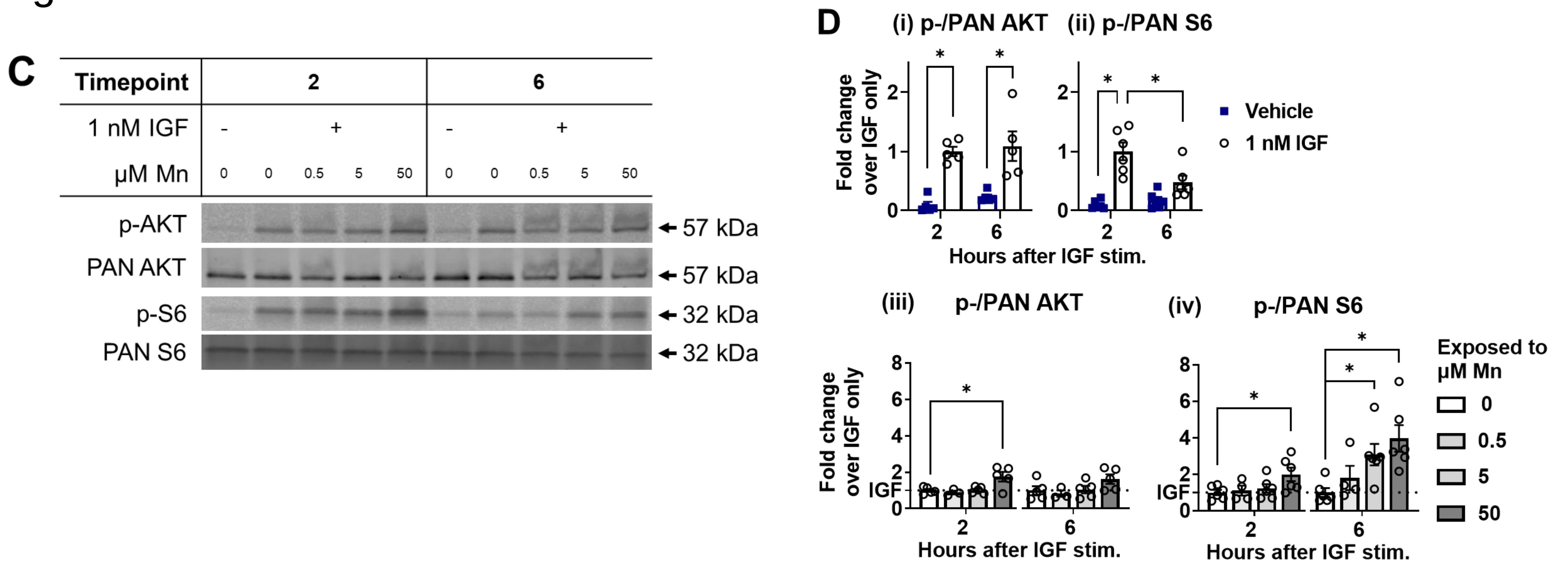

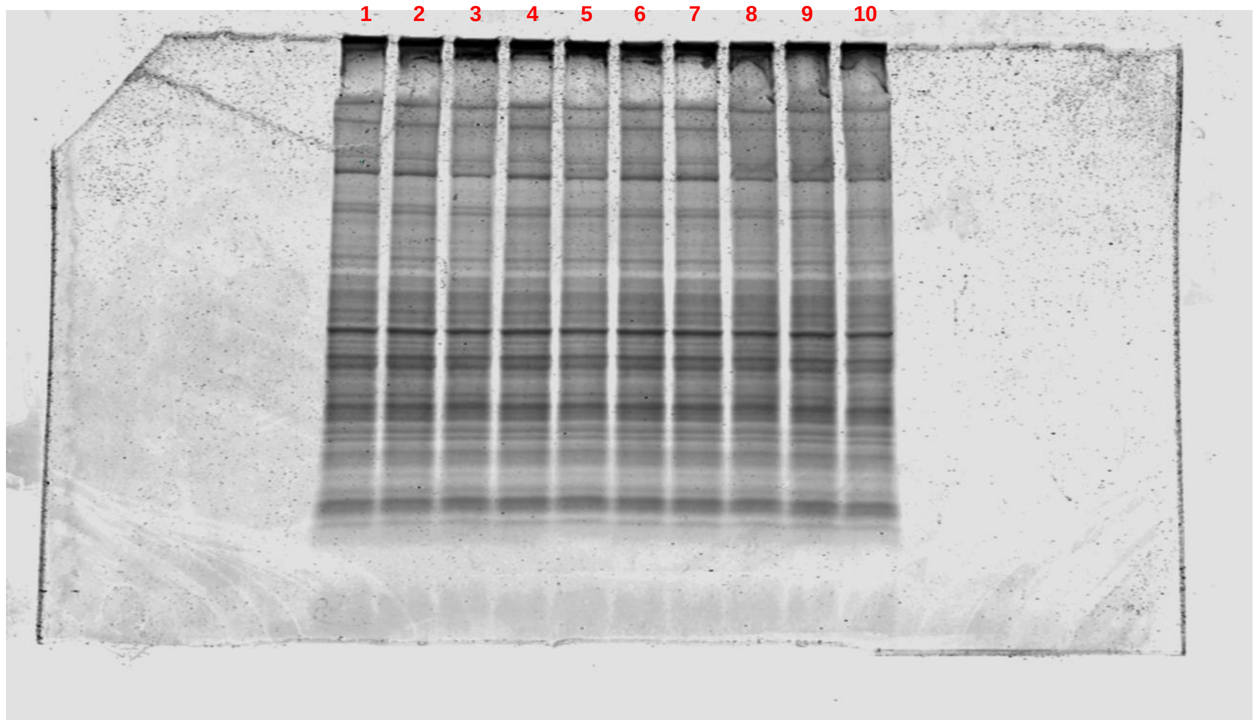

Lane #	Time point (h)	1 nM IGF	Mn (µM)
1	2	-	0
2		+	0
3		+	0.5
4		+	5
5		+	50
6	6	-	0
7		+	0
8		+	0.5
9		+	5
10		+	50

Sections displayed in Figure 4C were highlighted in red boxes.

p-AKT (Ser473)

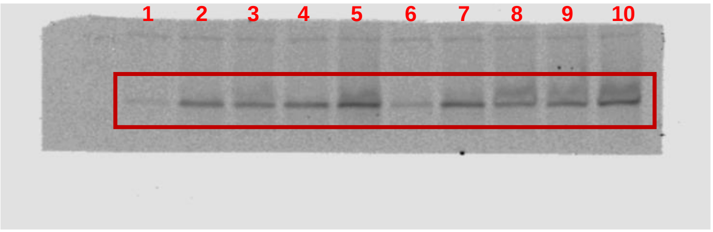

p-S6 (Ser235/236)

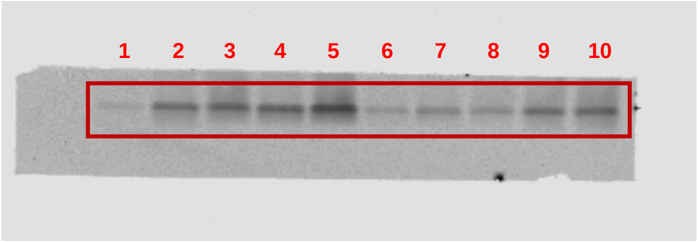

PAN-AKT

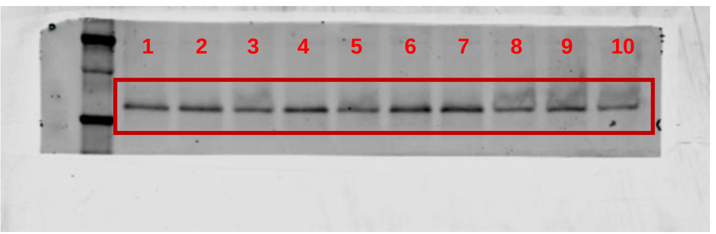

PAN-S6

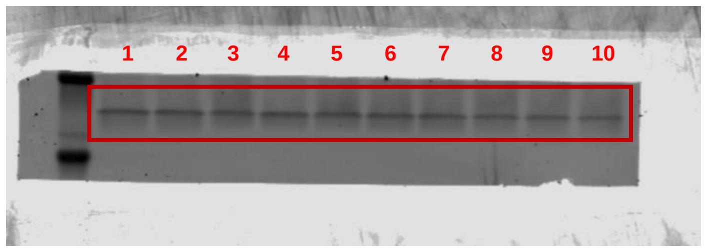

II. Original blots/gels displayed in supplemental figures

Original blots displayed in Supplemental Figure 1B and C

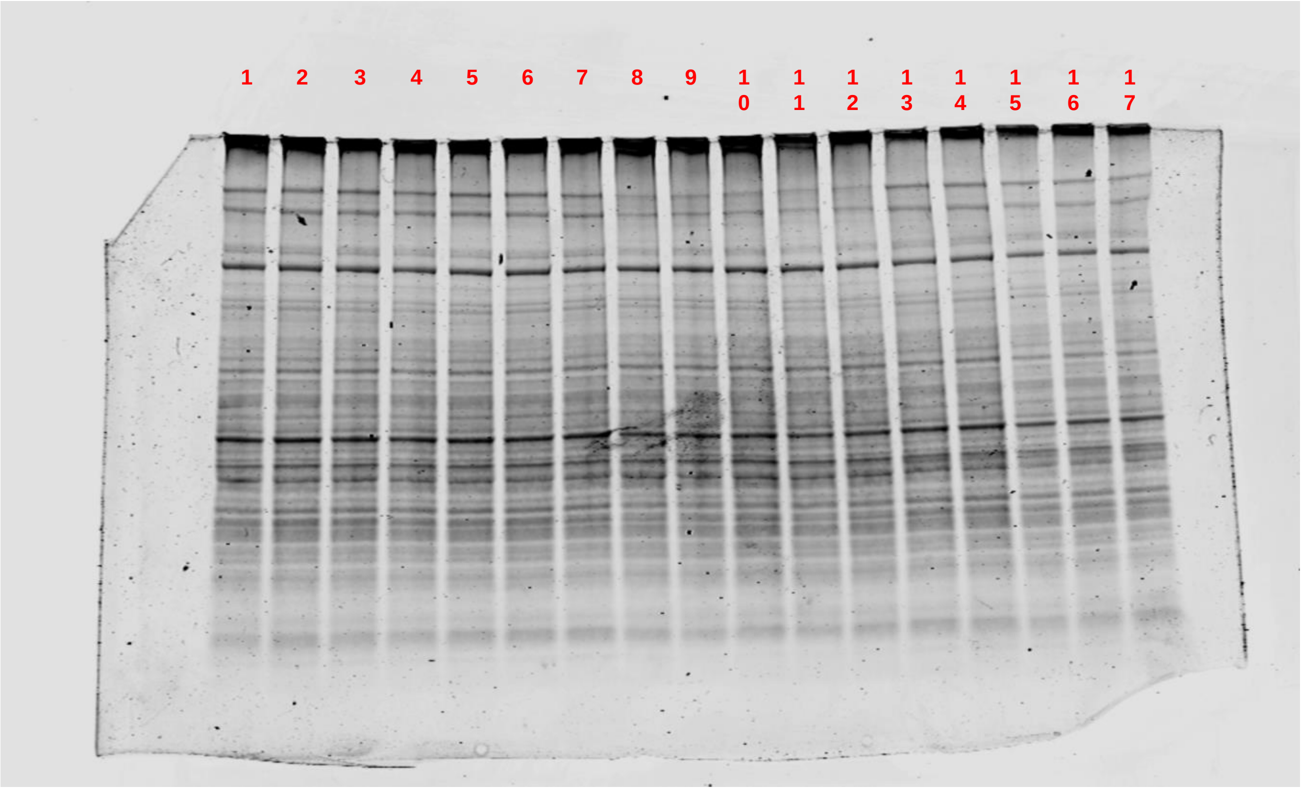

Lane#	Time point (h)	1 nM IGF	Mn (μM)
1	-1		0
2			0.5
3			5
4			50
5	0	-	0
6		-	5
7		-	50
8	2	-	0
9		+	0
10		+	0.5
11		+	5
12		+	50
13	6	-	0
14		+	0
15		+	0.5
16		+	5
17		+	50

Sections displayed in **Supplemental Figure 1B and C** were highlighted in red boxes.

p-mTOR (Ser2448)

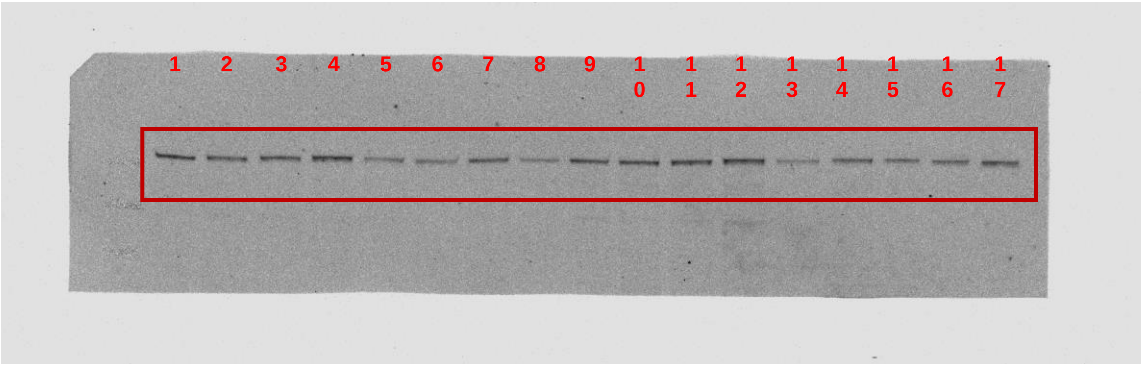

p-4E-BP1 (Thr37/46)

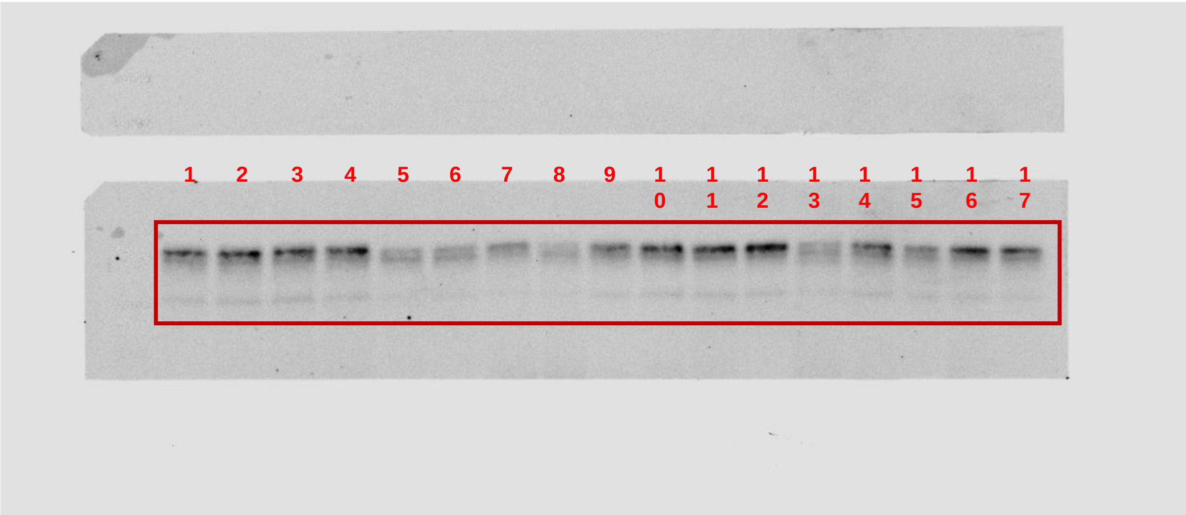

PAN mTOR

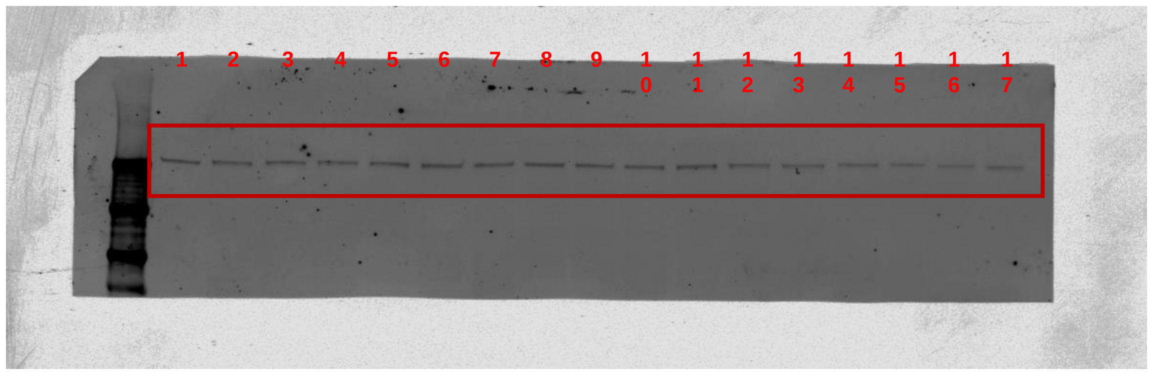

β-Actin

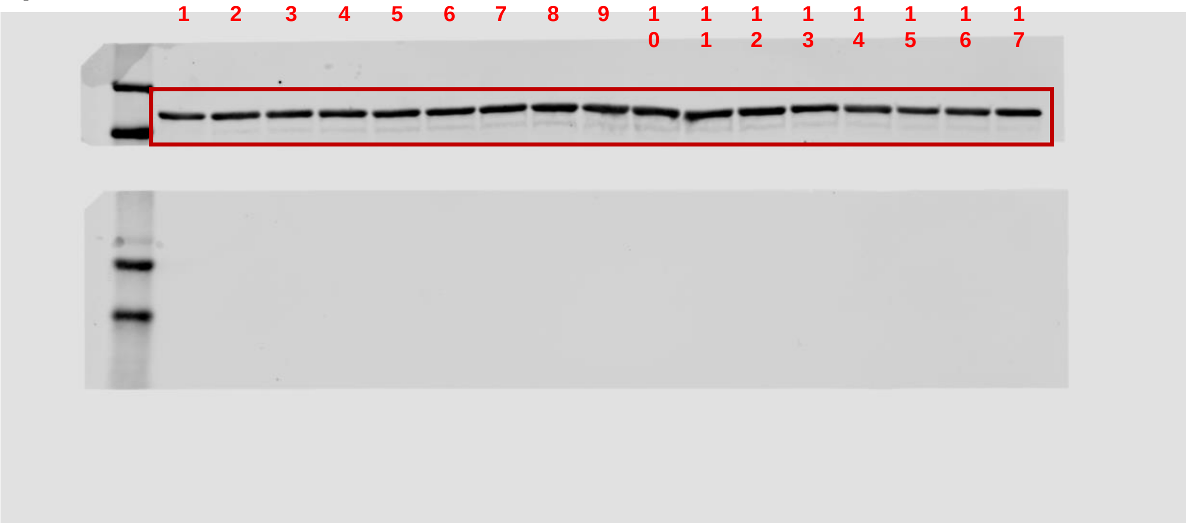

III. Independent biological replicates (BRs) for quantitative analyses

Biological replicate (BR) 3 in Figure 1C quantification

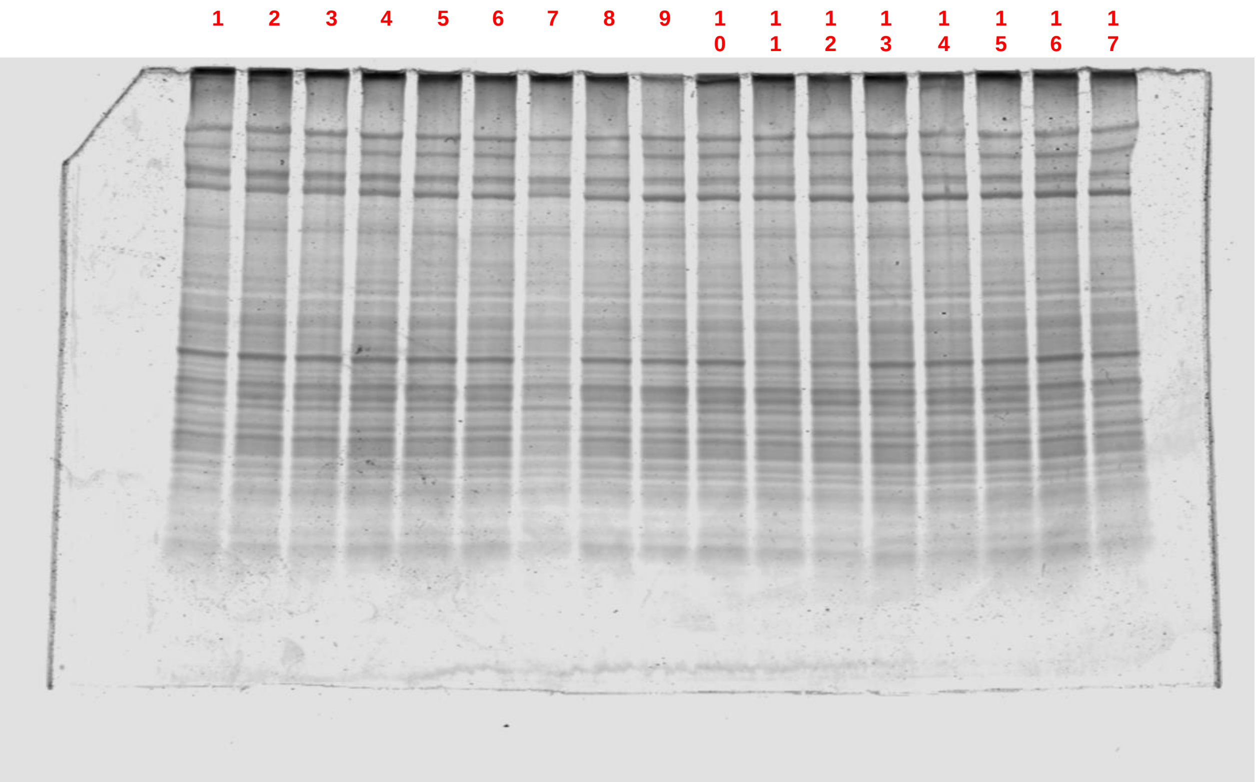

BR#	Lane#	1 nM IGF	Mn (μM)
3	1	-	0
	2	-	100
	3	-	200
	4	+	0
	5*	+	1
	6	+	5
	7	+	10
	8	+	25
	9	+	50
	10	+	100
	11	+	200
1	12	-	0
	13	-	100
	14	-	200
	15	+	0
	16*	+	1
	17	+	5

* used as loading control for normalization during quantification

p-AKT (Ser473)

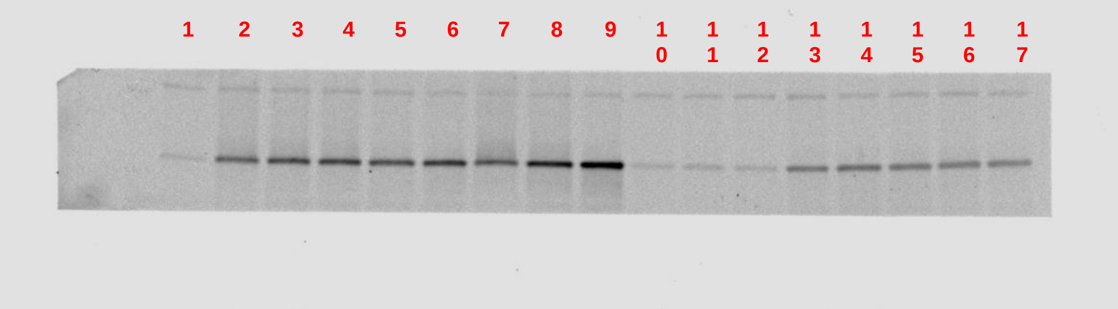

p-S6 (Ser235/236)

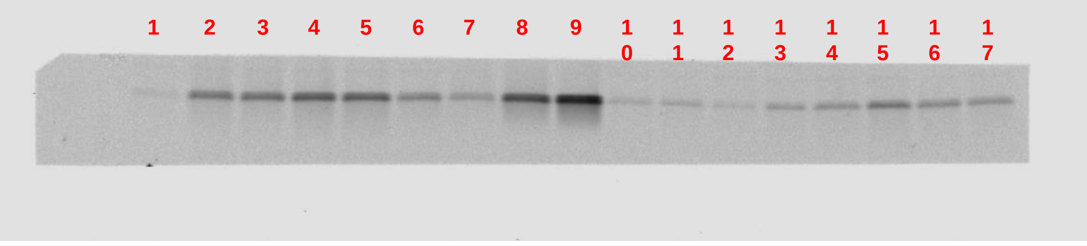

PAN-AKT

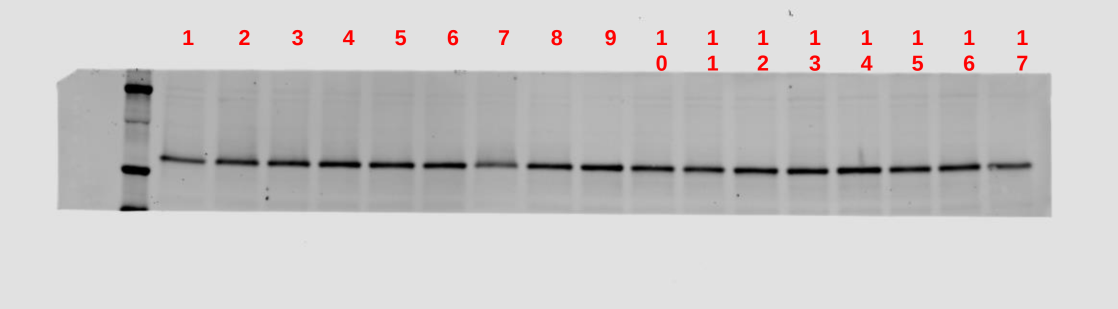

PAN-S6

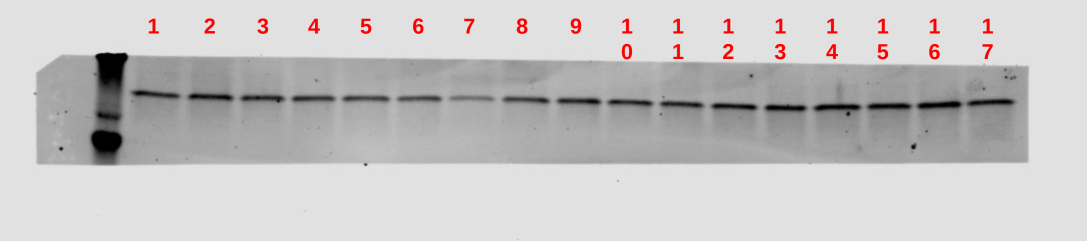

BR4 in Figure 1C quantification

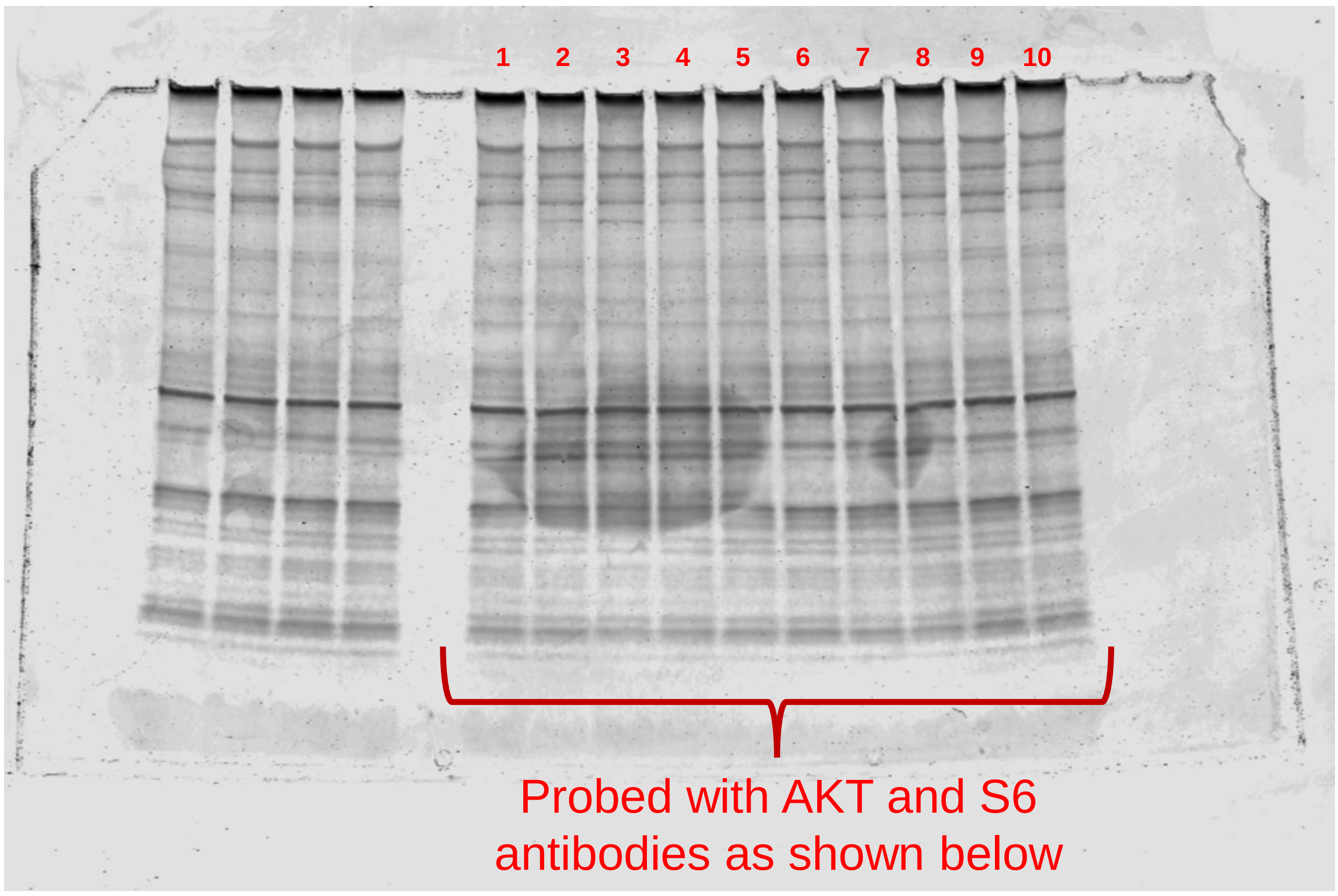

Lane#	1 nM IGF	Mn (μM)
1	-	0
2	+	0
3*	+	1
4	+	10
5	+	25
6	+	50
7	+	100
8	+	200
9	-	100
10	-	200

* used as loading control for normalization during quantification

p-AKT (Ser473)

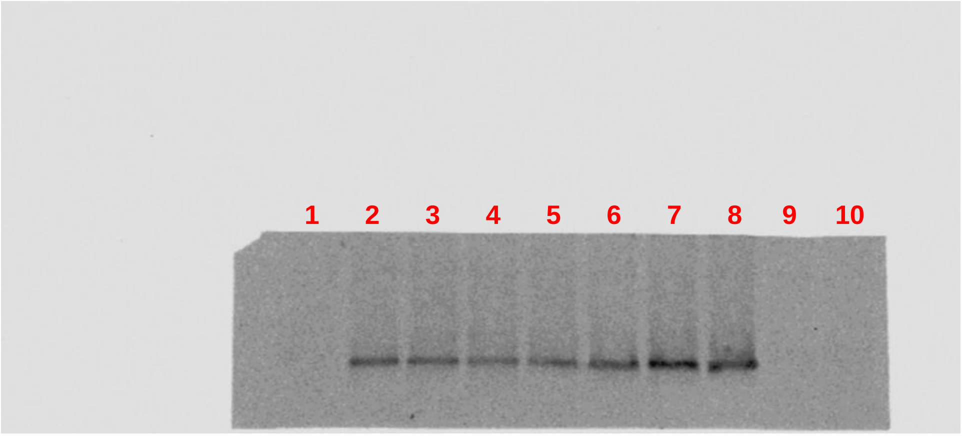

p-S6 (Ser235/236)

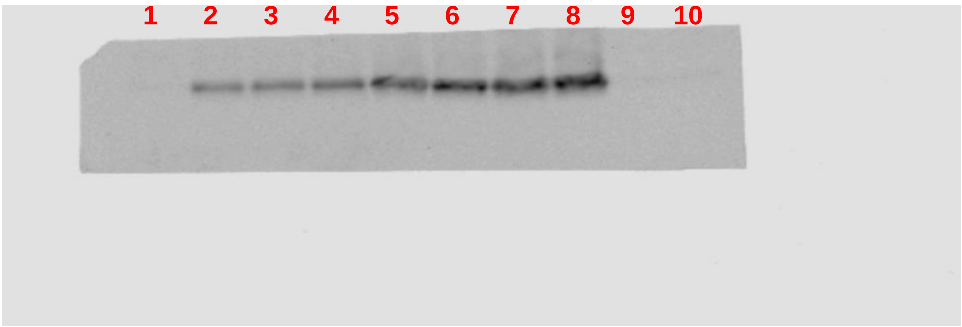

PAN-AKT

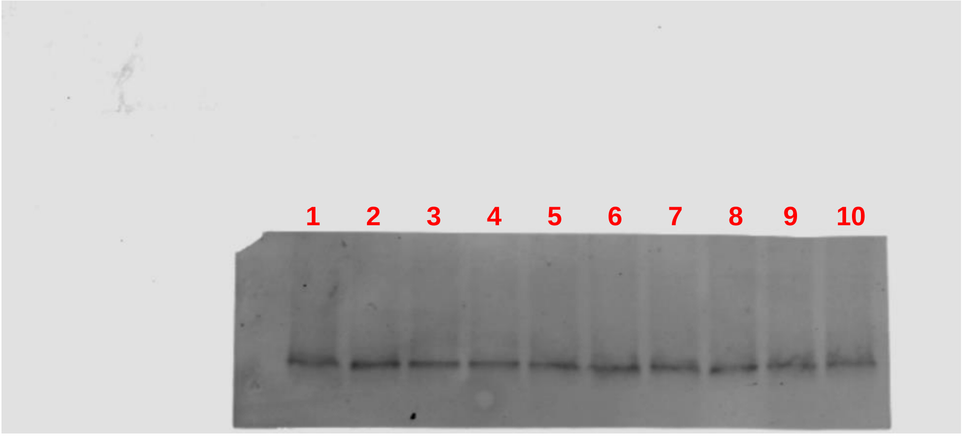

PAN-S6

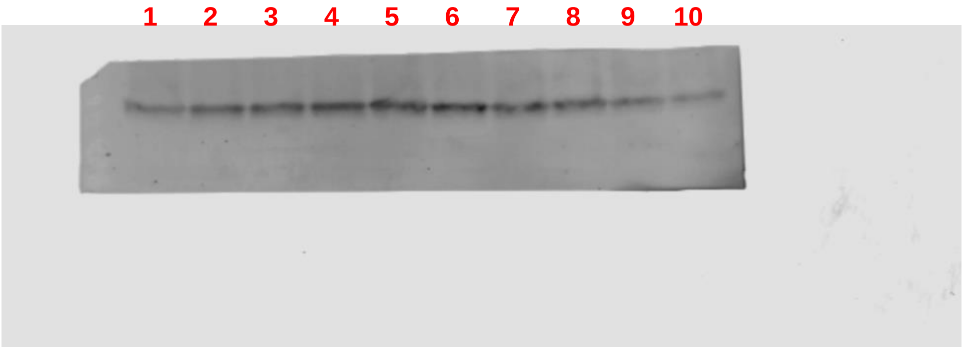

BR5 in Figure 1C quantification

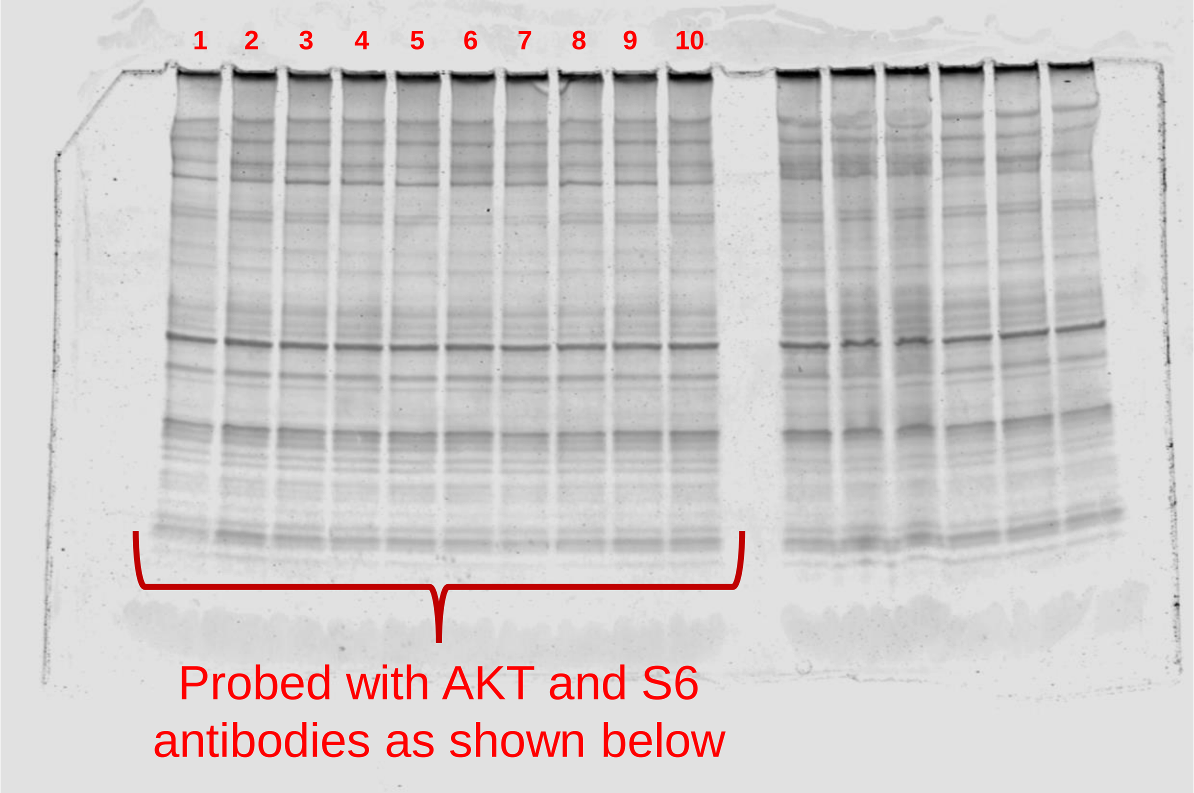

Lane#	1 nM IGF	Mn (μM)
1	-	0
2	+	0
3*	+	1
4	+	10
5	+	25
6	+	50
7	+	100
8	+	200
9	-	100
10	-	200

* used as loading control for normalization during quantification

p-AKT (Ser473)

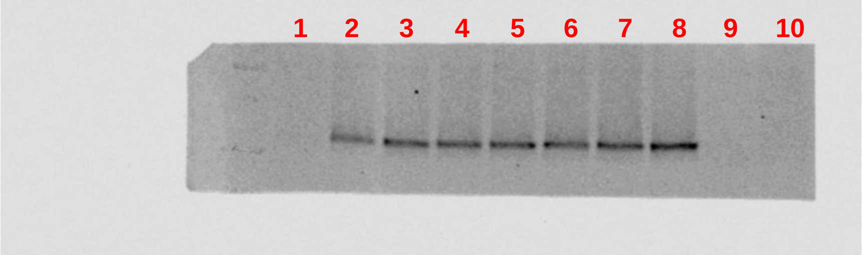

p-S6 (Ser235/236)

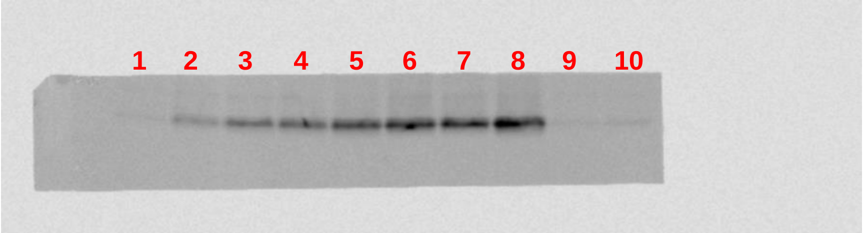

PAN-AKT

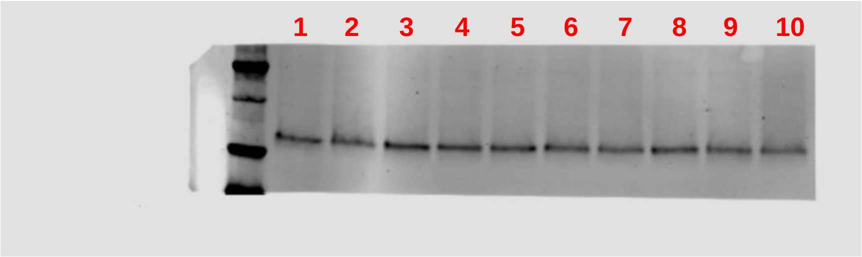

PAN-S6

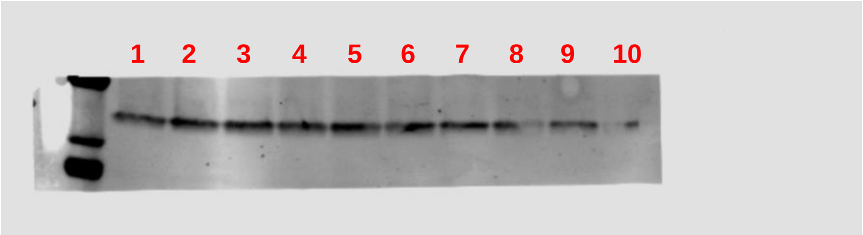

BR6 in Figure 1C quantification

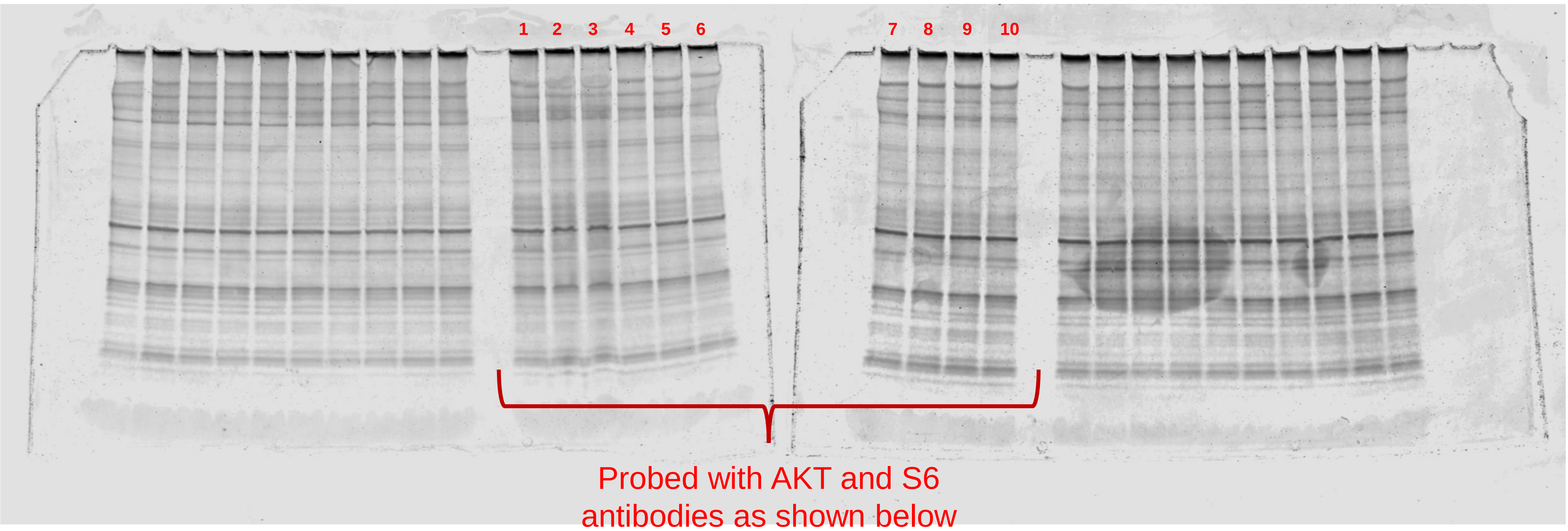

p-AKT (Ser473)

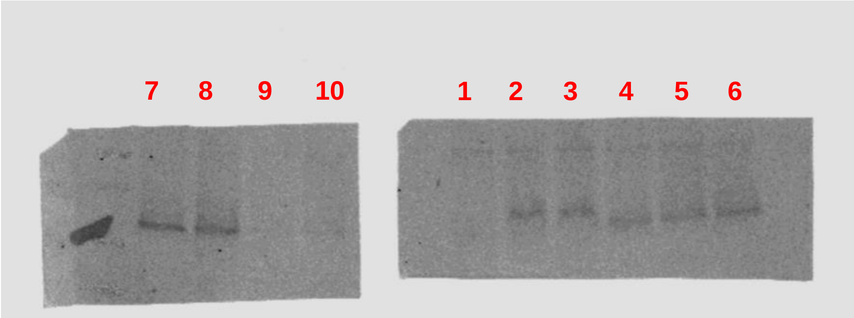

PAN-AKT

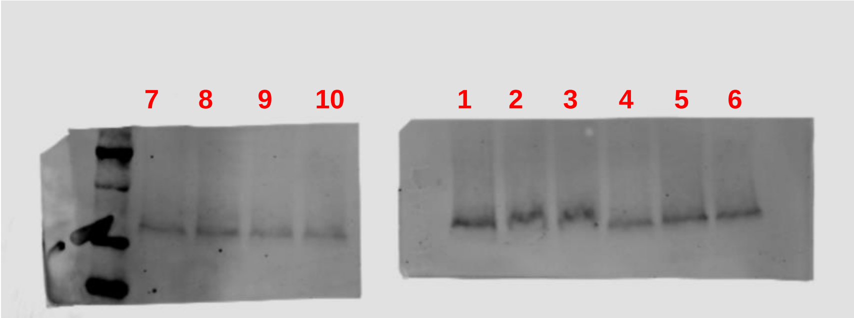

p-S6 (Ser235/236)

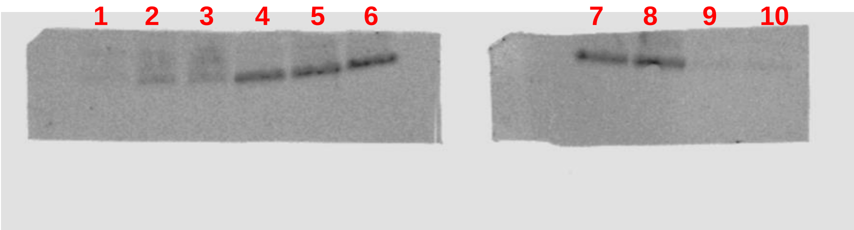

PAN-S6

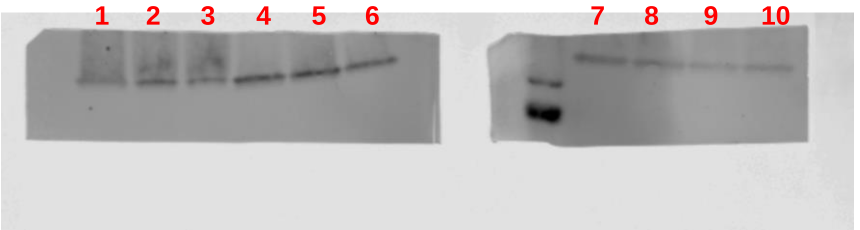

Lane#	1 nM IGF	Mn (μM)
1	-	0
2	+	0
3*	+	1
4	+	10
5	+	25
6	+	50
7	+	100
8	+	200
9	-	100
10	-	200

* used as loading control for normalization during quantification

Note that although the samples were run on separate gels, the blots were simultaneously probed and scanned, therefore allowing comparison across blots

BR2 in Figure 2A(iii) quantification

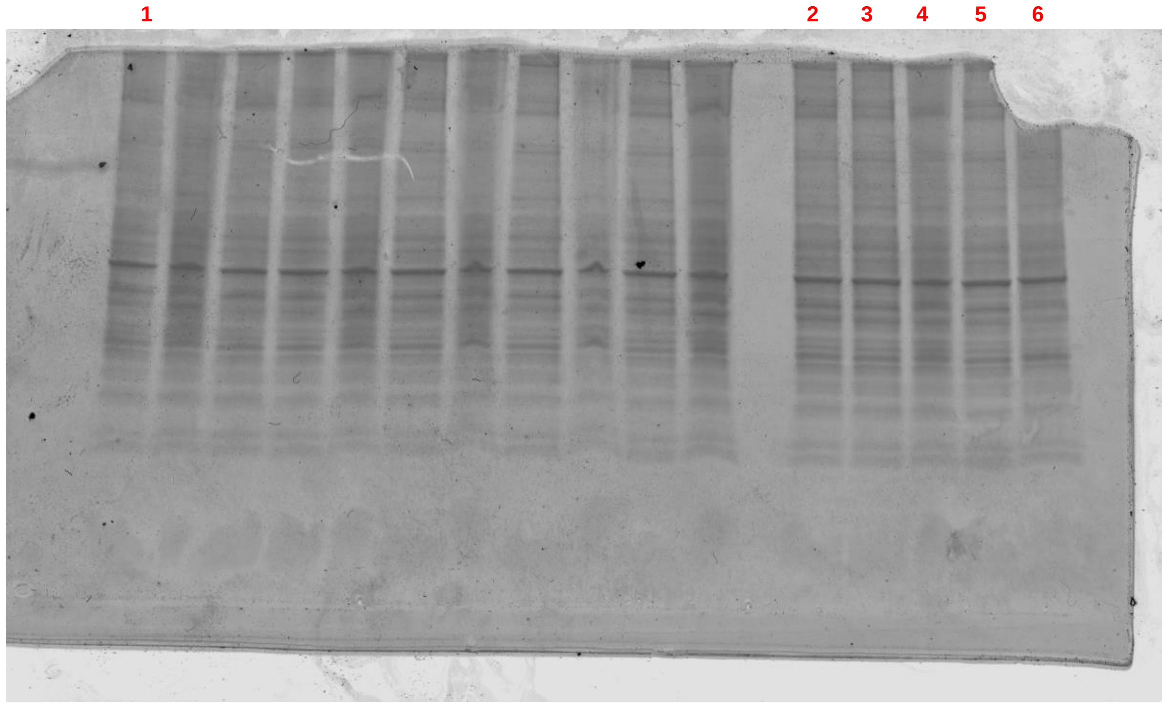

Lane#	1 nM IGF	Mn (µM)	IGFR inhibitor
1*	-	0	-
2	+	0	-
3	+	100	-
4	+	100	BMS5 (200 µM)
5	+	100	NVP (1mM)
6	+	100	Lins (1 mM)

* used as loading control for normalization during quantification
BMS5 = BMS-536924; NVP = NVP-AEW541; Lins = Linsitinib

Only bands at molecular weight indicated by arrows represent targeted proteins.

p-AKT (Ser473)

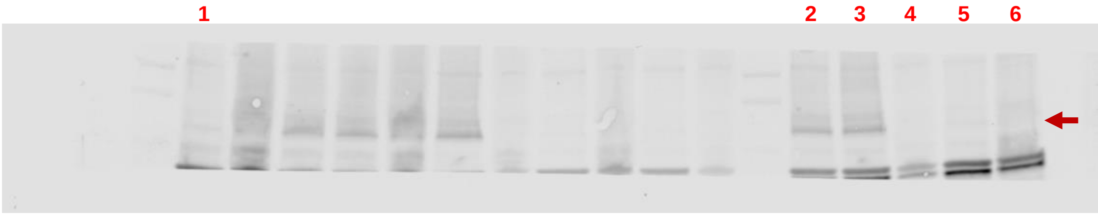

p-S6 (Ser235/236)

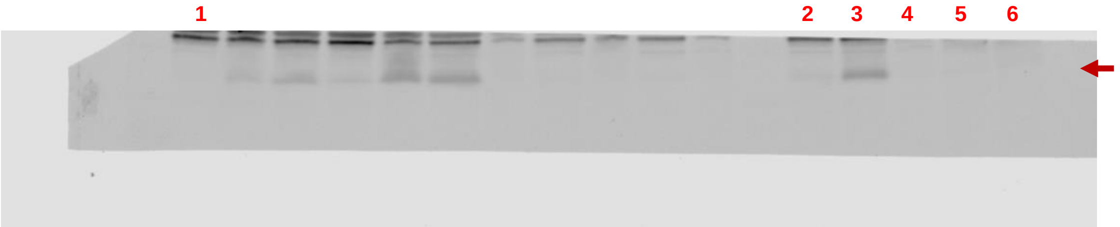

PAN-AKT

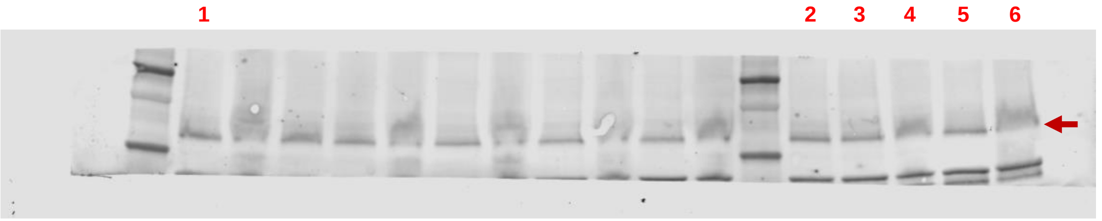

PAN-S6

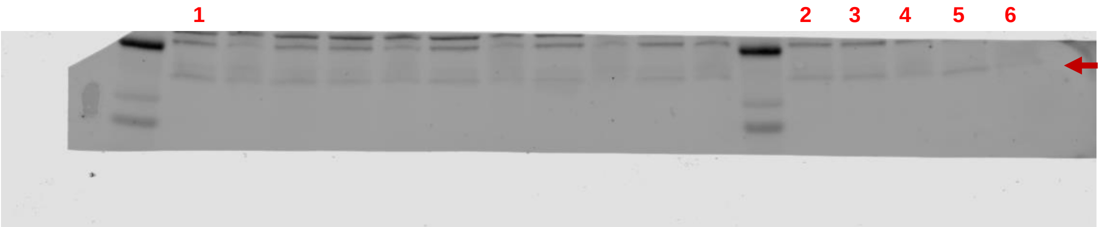

BR3 in Figure 2A(iii) quantification

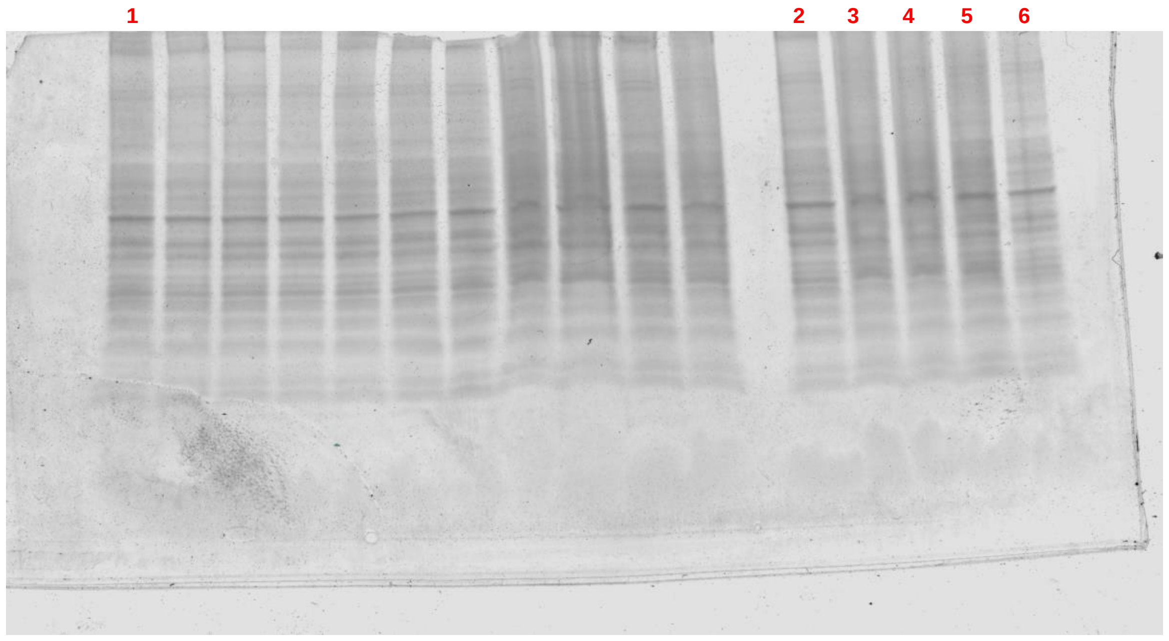

Lane#	1 nM IGF	Mn (μM)	IGFR inhibitor
1*	-	0	-
2	+	0	-
3	+	100	-
4	+	100	BMS5 (200 μM)
5	+	100	NVP (1mM)
6	+	100	Lins (1 mM)

* used as loading control for normalization during quantification.
BMS5 = BMS-536924; NVP = NVP-AEW541; Lins = Linsitinib

Only bands at molecular weight indicated by arrows represent targeted proteins.

p-AKT (Ser473)

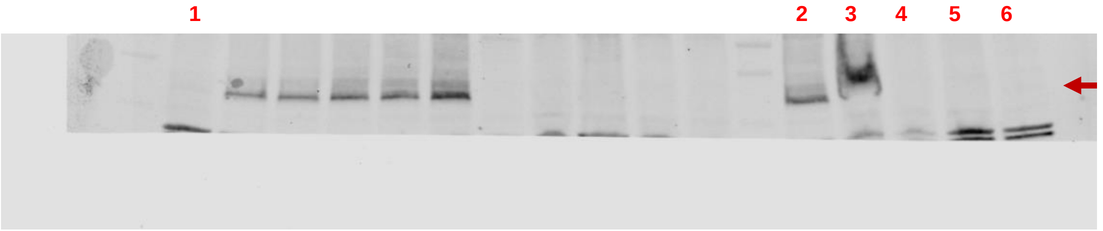

p-S6 (Ser235/236)

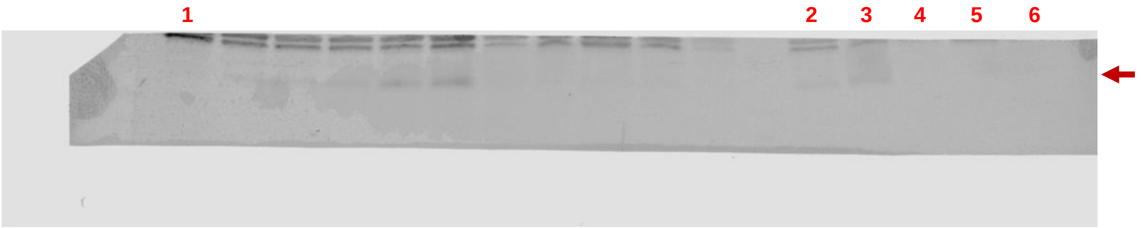

PAN-AKT

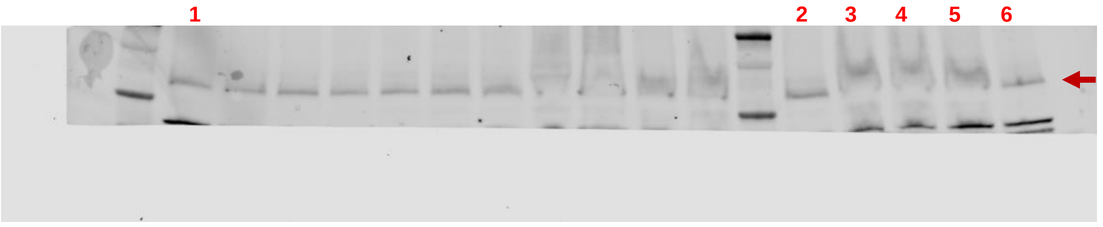

PAN-S6

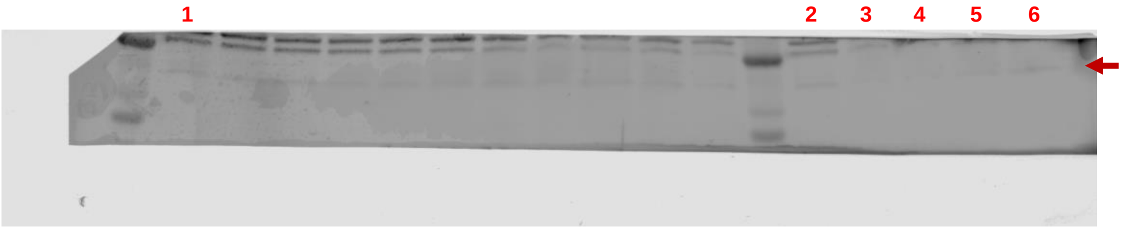

BR2 in Figure 2B(iii) quantification

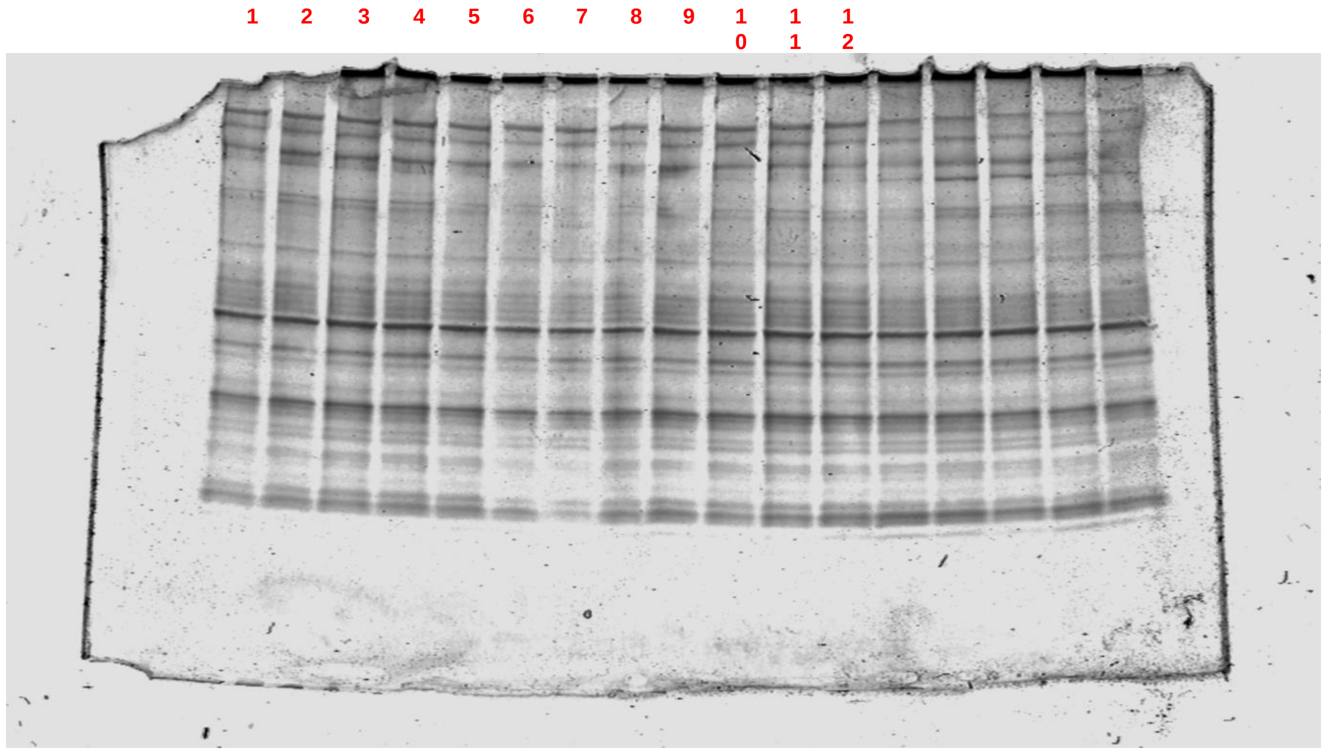

<table>
<tr><th>Lane#</th><th>Essential Amino Acids</th><th>1 nM IGF</th><th>100 µM Mn</th><th>200 µM BMS5</th></tr>
<tr><td>1</td><td rowspan="6">+</td><td rowspan="3">-</td><td>-</td><td>-</td></tr>
<tr><td>2</td><td>+</td><td>-</td></tr>
<tr><td>3</td><td>+</td><td>+</td></tr>
<tr><td>4</td><td rowspan="3">+</td><td>-</td><td>-</td></tr>
<tr><td>5</td><td>+</td><td>-</td></tr>
<tr><td>6*</td><td>+</td><td>+</td></tr>
<tr><td>7</td><td rowspan="6">-</td><td rowspan="3">-</td><td>-</td><td>-</td></tr>
<tr><td>8</td><td>+</td><td>-</td></tr>
<tr><td>9</td><td>+</td><td>+</td></tr>
<tr><td>10</td><td rowspan="3">+</td><td>-</td><td>-</td></tr>
<tr><td>11</td><td>+</td><td>-</td></tr>
<tr><td>12</td><td>+</td><td>+</td></tr>
</table>

* used as loading control for normalization during quantification.

p-AKT (Ser473)

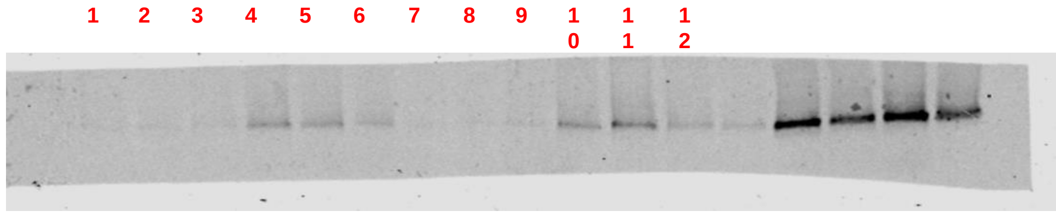

p-S6 (Ser235/236)

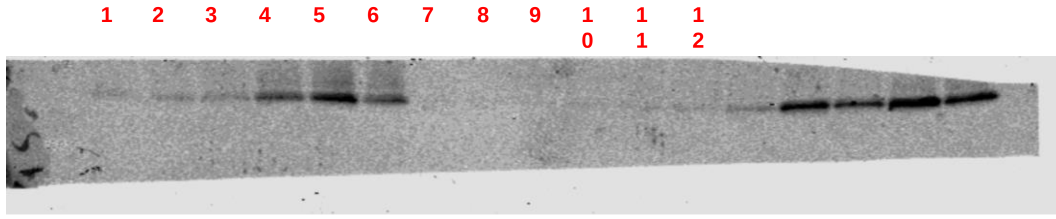

PAN-AKT

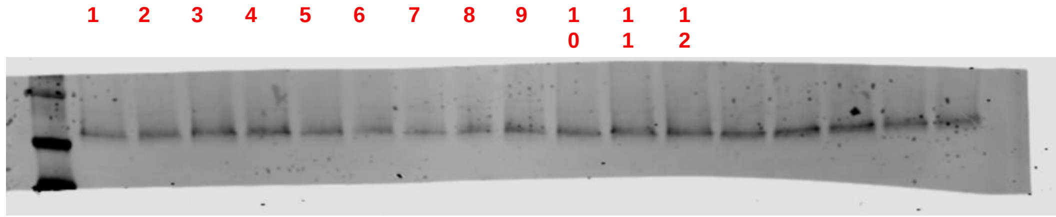

PAN-S6

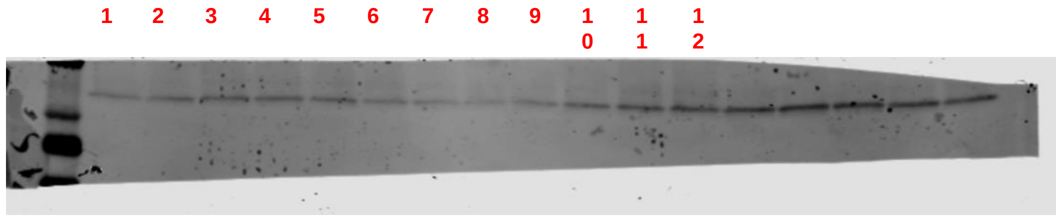

BR3 in Figure 2B(iii) quantification

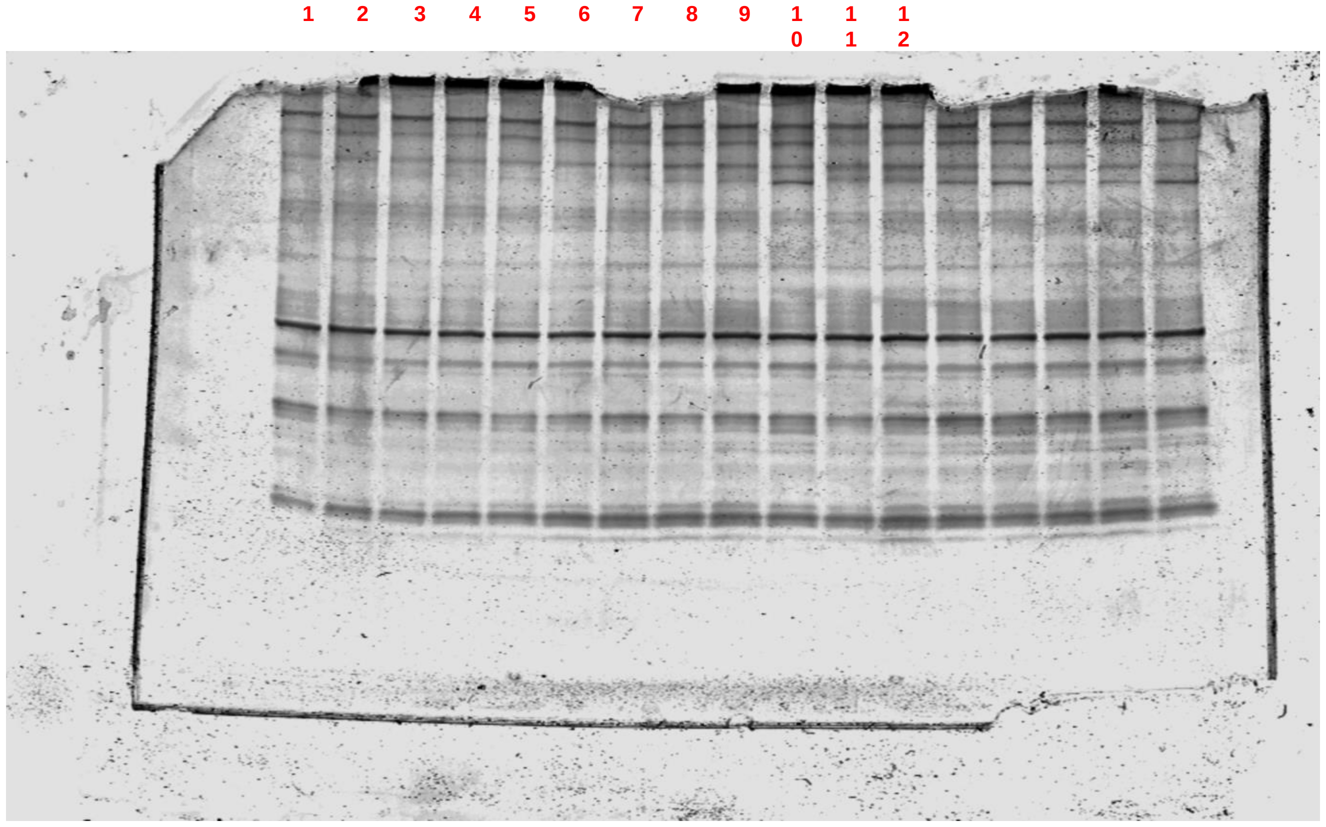

<table>
<tr><th>Lane#</th><th>Essential Amino Acids</th><th>1 nM IGF</th><th>100 μM Mn</th><th>200 μM BMS5</th></tr>
<tr><td>1</td><td rowspan="6">+</td><td rowspan="3">-</td><td>-</td><td>-</td></tr>
<tr><td>2</td><td>+</td><td>-</td></tr>
<tr><td>3</td><td>+</td><td>+</td></tr>
<tr><td>4</td><td rowspan="3">+</td><td>-</td><td>-</td></tr>
<tr><td>5</td><td>+</td><td>-</td></tr>
<tr><td>6*</td><td>+</td><td>+</td></tr>
<tr><td>7</td><td rowspan="6">-</td><td rowspan="3">-</td><td>-</td><td>-</td></tr>
<tr><td>8</td><td>+</td><td>-</td></tr>
<tr><td>9</td><td>+</td><td>+</td></tr>
<tr><td>10</td><td rowspan="3">+</td><td>-</td><td>-</td></tr>
<tr><td>11</td><td>+</td><td>-</td></tr>
<tr><td>12</td><td>+</td><td>+</td></tr>
</table>

* used as loading control for normalization during quantification.

p-AKT (Ser473)

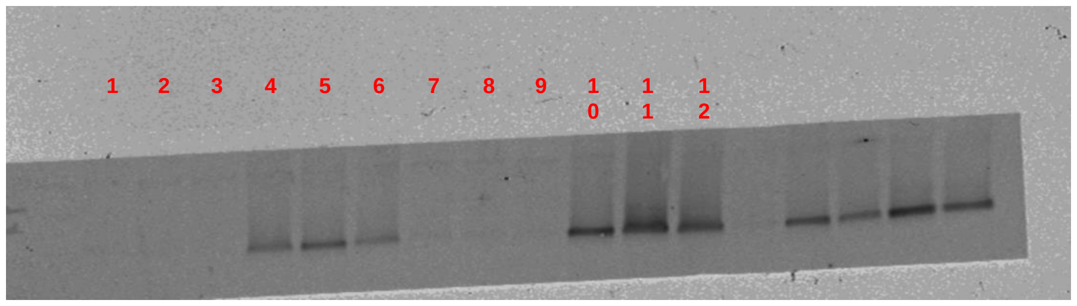

p-S6 (Ser235/236)

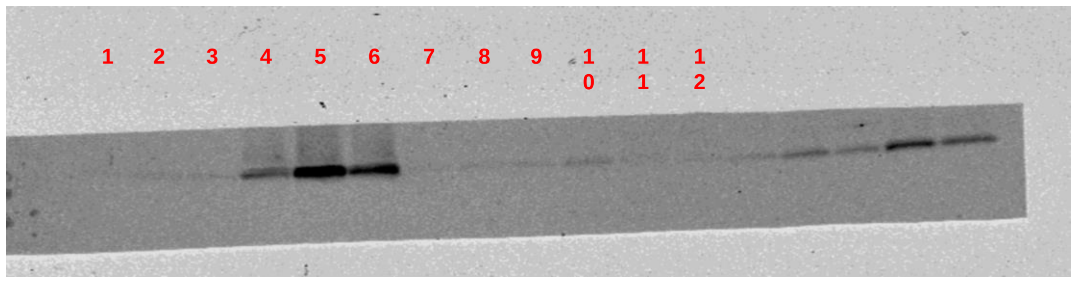

PAN-AKT

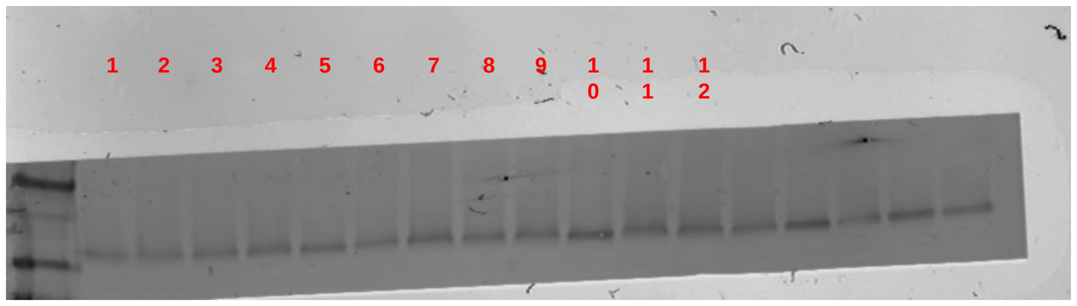

PAN-S6

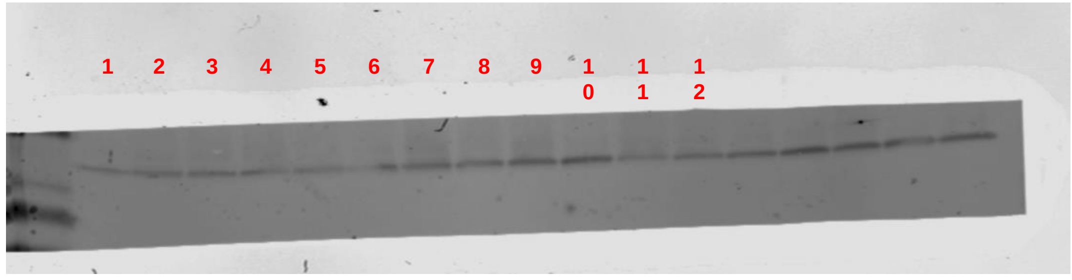

BR2-1 in Figure 3D (i-iii) quantification

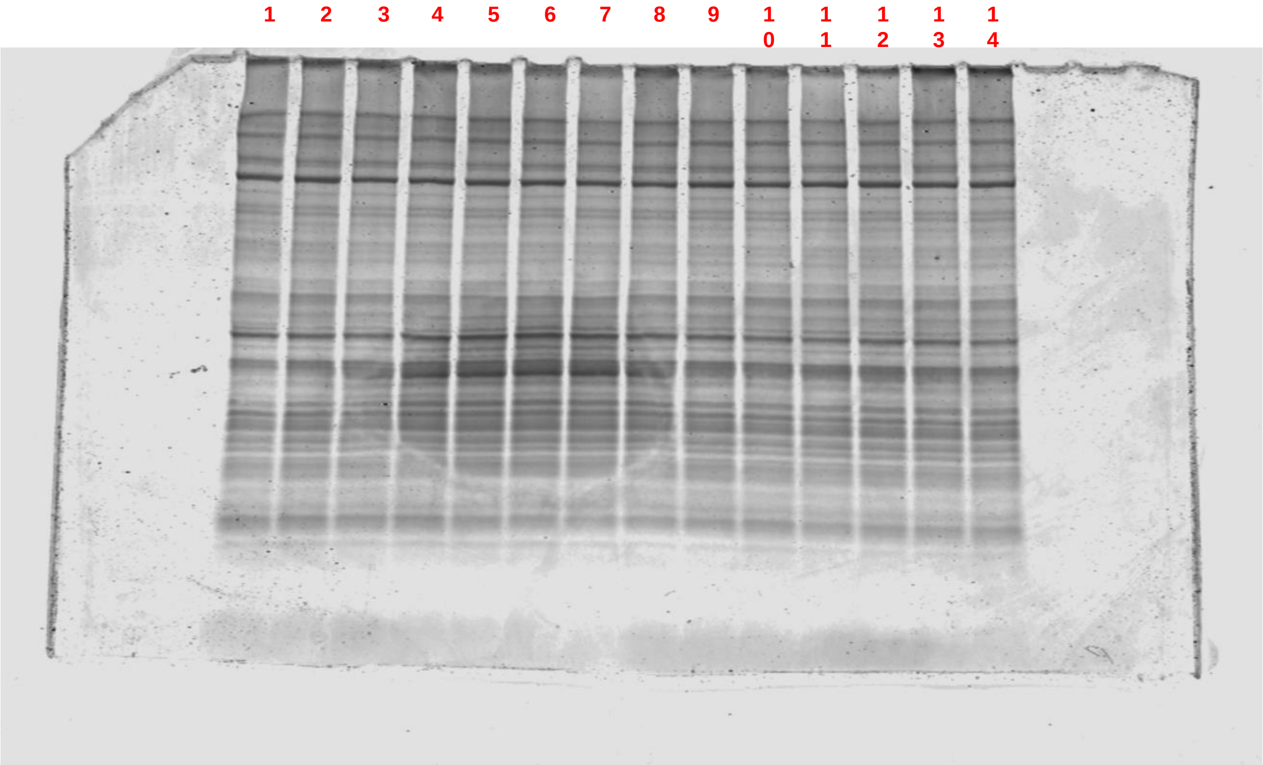

Lane#	Time point (h)	1 nM IGF	Mn (μM)
1	-1	-	0
2	0	-	0
3	0.25	-	0
4		+	0
5		+	50
6		+	200
7	0.5	-	0
8		+	0
9		+	50
10		+	200
11	1	-	0
12		+	0
13		+	50
14		+	200

p-AKT (Ser473)

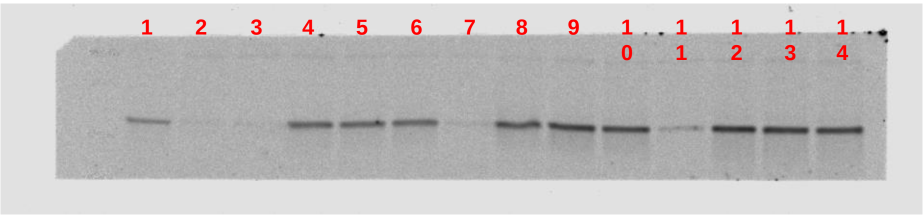

p-S6 (Ser235/236)

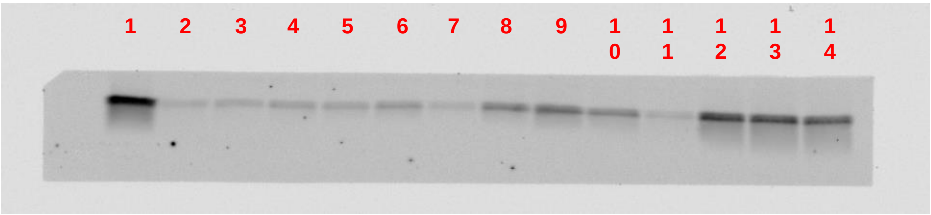

PAN-AKT

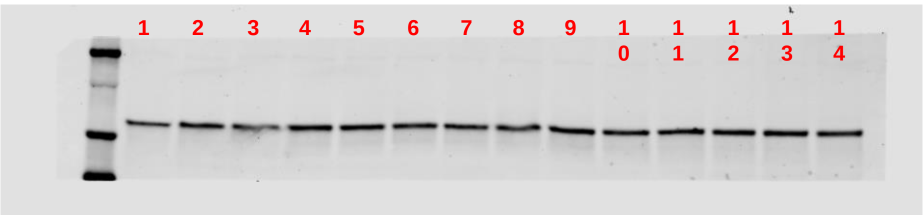

PAN-S6

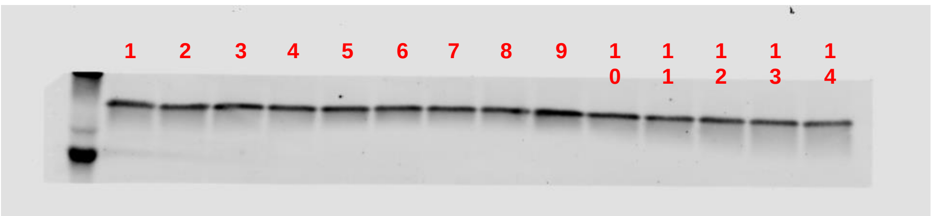

BR2-2 in Figure 3D (i-iii) quantification

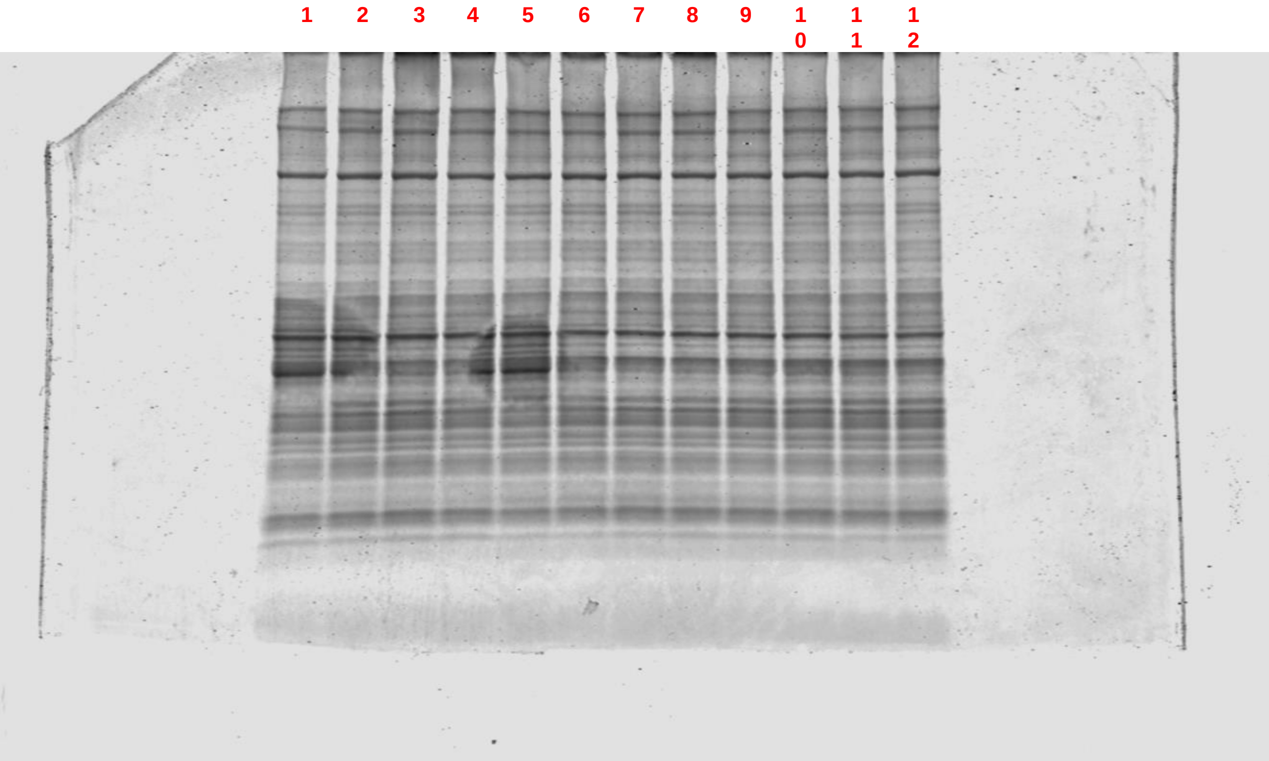

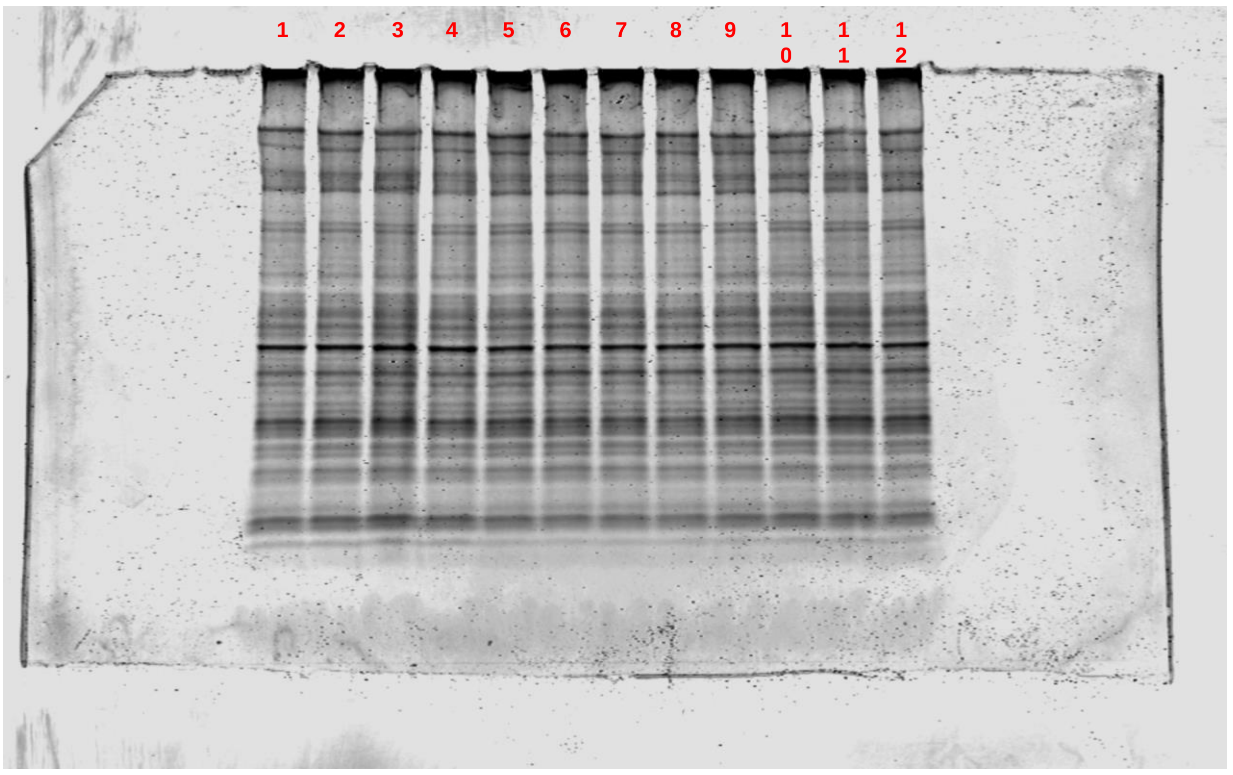

p-AKT (Ser473)

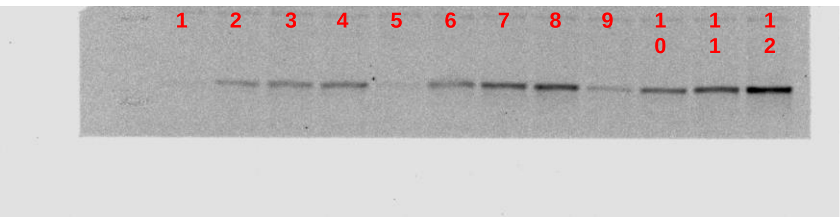

p-S6 (Ser235/236)

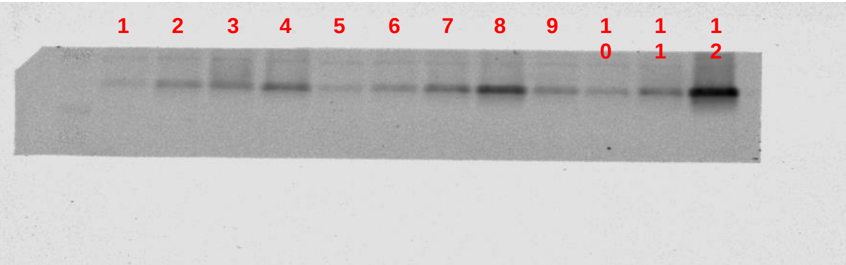

PAN-AKT

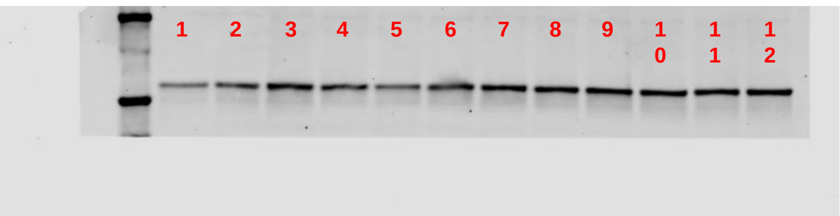

PAN-S6

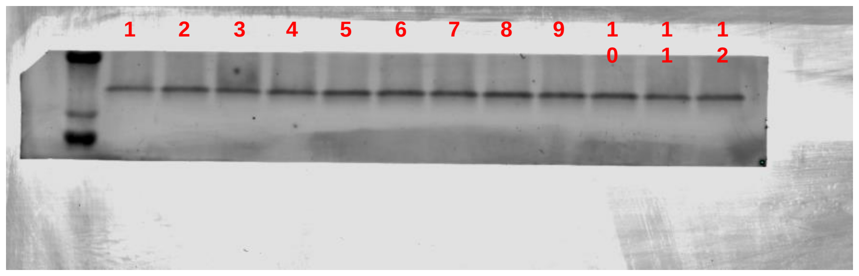

Lane#	Time point (h)	1 nM IGF	Mn (μM)
1	2	-	0
2		+	0
3		+	50
4		+	200
5	3	-	0
6		+	0
7		+	50
8		+	200
9	6	-	0
10		+	0
11		+	50
12		+	200

Note: Both gels were loaded with the same set of samples, in the same order as shown in the table; then separately probed with AKT or S6.

BR3 in Figure 3D(ii) quantification

Note: Only bands representing AKT expression level at time point 2, 3, and 6 were included in Figure 3D(ii) quantification processes.

Lane#	Time point (h)	1 nM IGF	Mn (μM)
1	-1	-	0
2	0	-	0
3	0.25	-	0
4		+	0
5		+	50
6		+	200
7	1	-	0
8		+	0
9		+	50
10		+	200
11	2	-	0
12		+	0
13		+	50
14		+	200

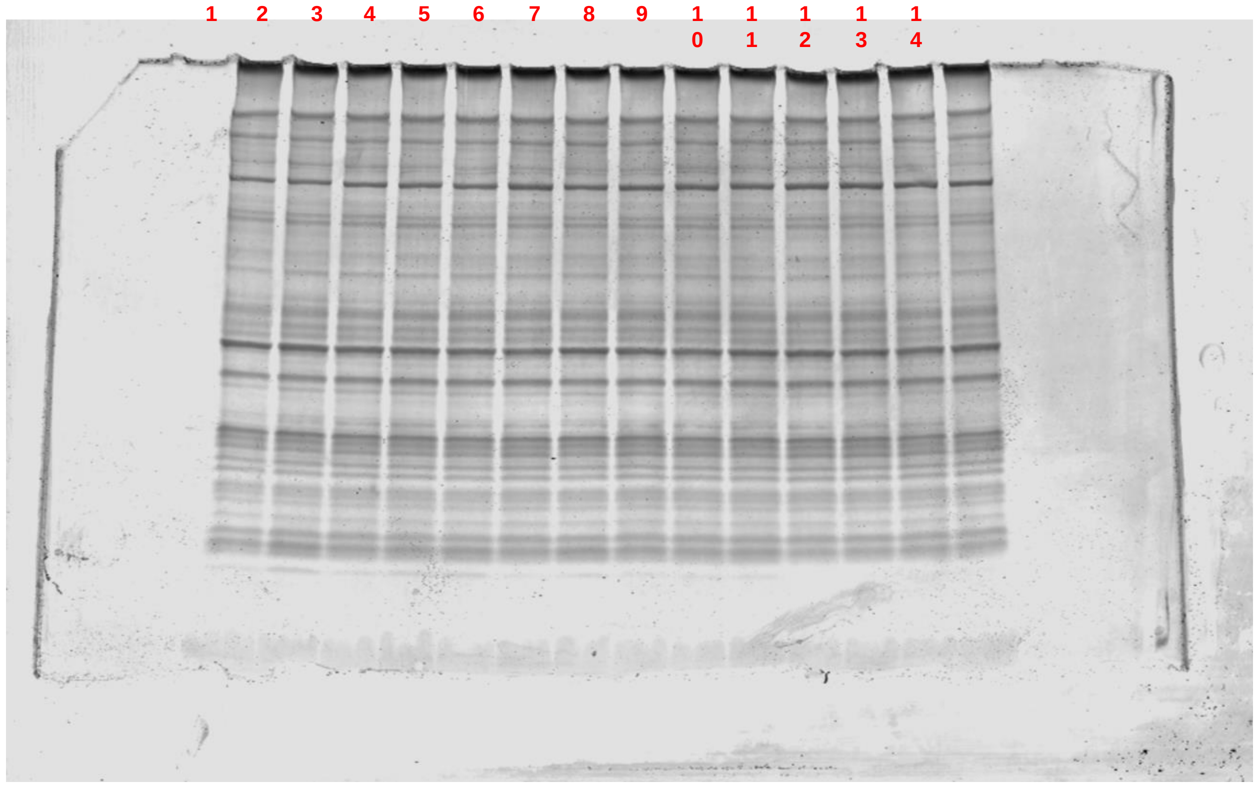

p-AKT (Ser473)

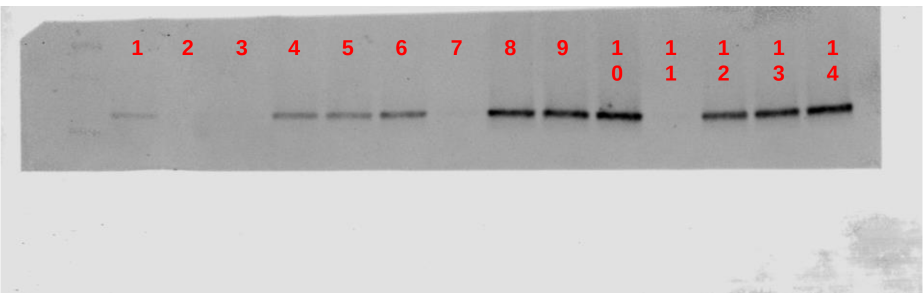

PAN-AKT

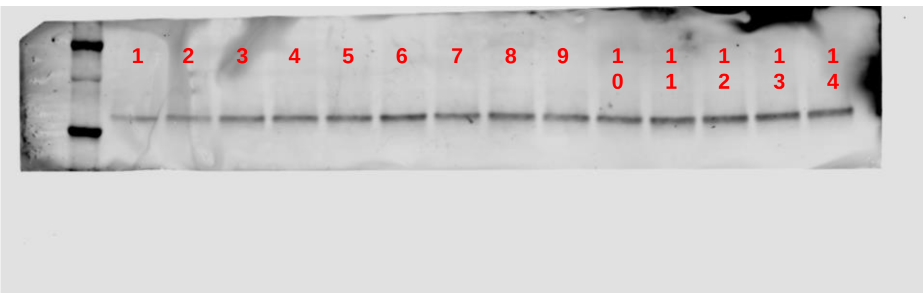

Lane #	Time point (h)	1 nM IGF	Mn (μM)
1	0.5	-	0
2		+	0
3		+	50
4		+	200
5	3	-	0
6		+	0
7		+	50
8		+	200
9	6	-	0
10		+	0
11		+	50
12		+	200

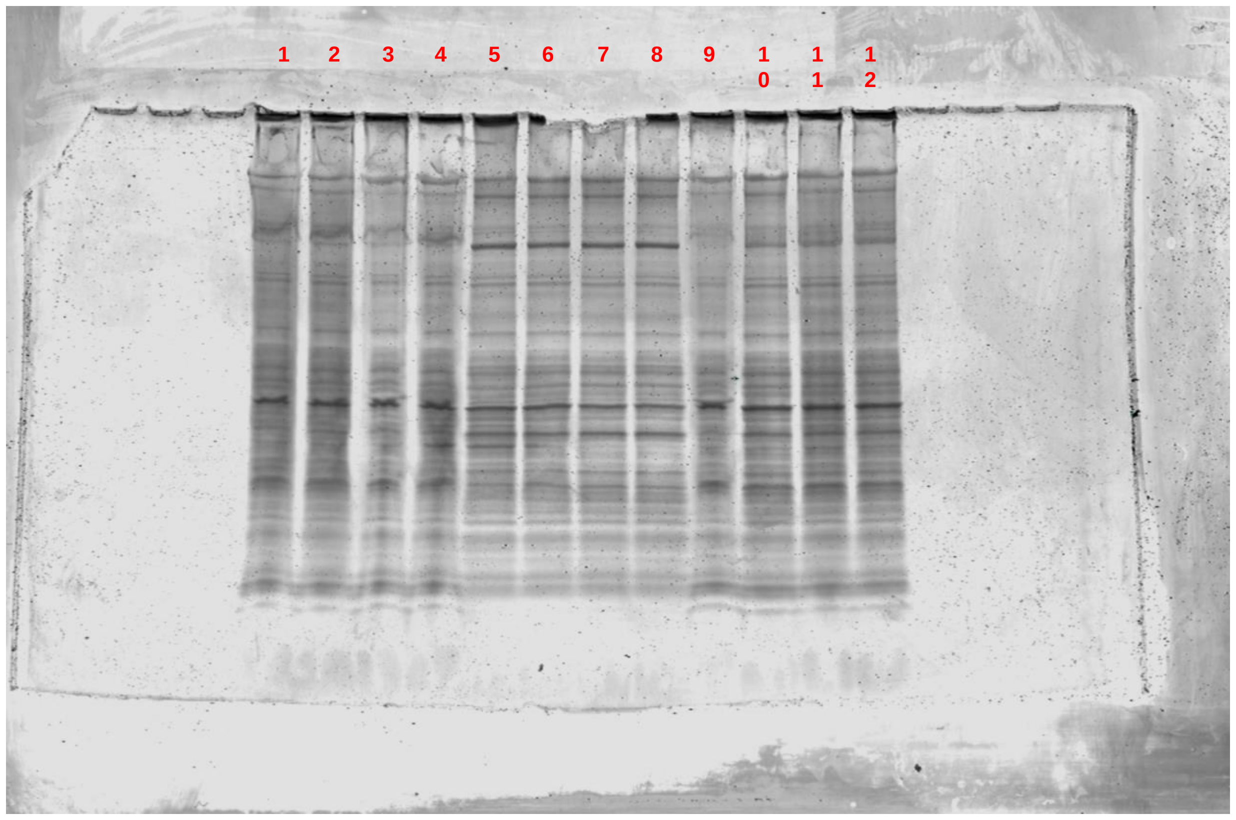

p-AKT (Ser473)

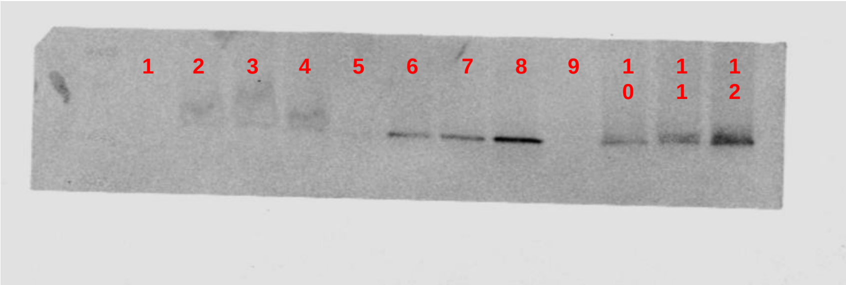

PAN-AKT

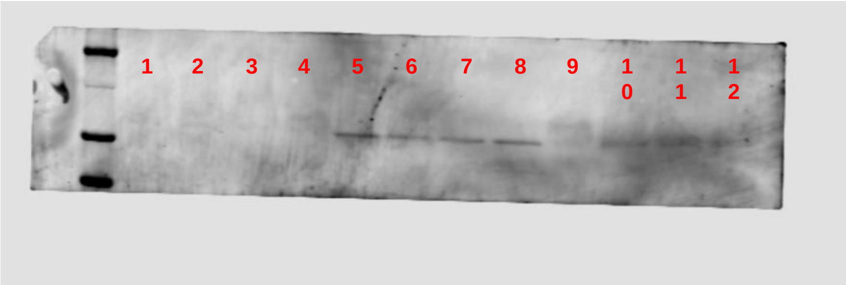

BR3 in Figure 3D(iii) quantification

Lane #	Time point (h)	1 nM IGF	Mn (µM)
1	2	-	0
2		+	0
3		+	50
4		+	200
5	3	-	0
6		+	0
7		+	50
8		+	200
9	6	-	0
10		+	0
11		+	50
12		+	200

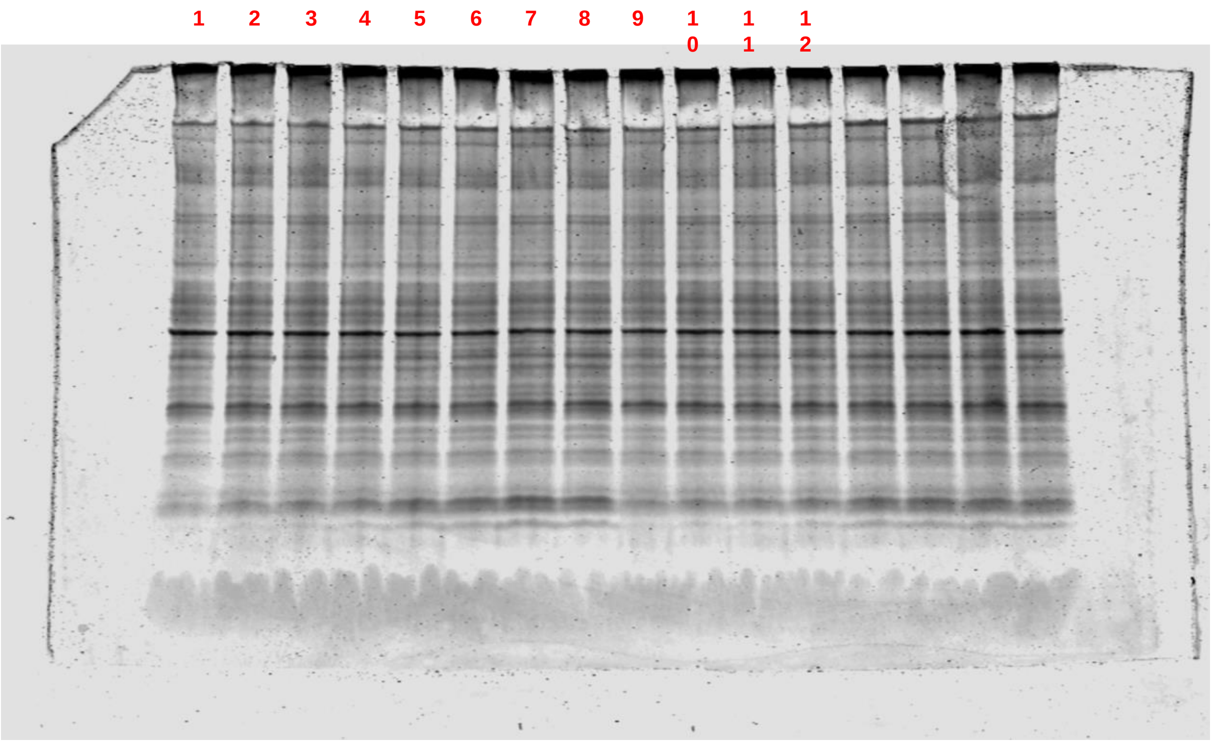

p-S6 (Ser235/236)

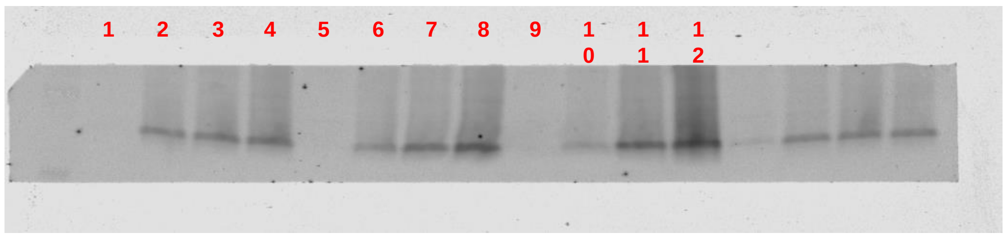

PAN-S6

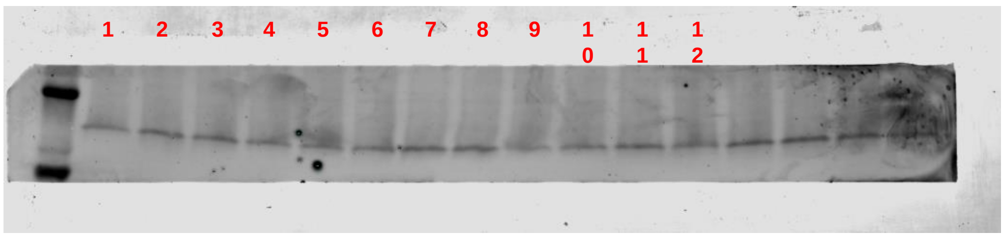

BR4 in Figure 3D(iii) quantification

Lane #	Time point (h)	1 nM IGF	Mn (µM)
1	2	-	0
2		+	0
3		+	50
4		+	200
5	3	-	0
6		+	0
7		+	50
8		+	200
9	6	-	0
10		+	0
11		+	50
12		+	200

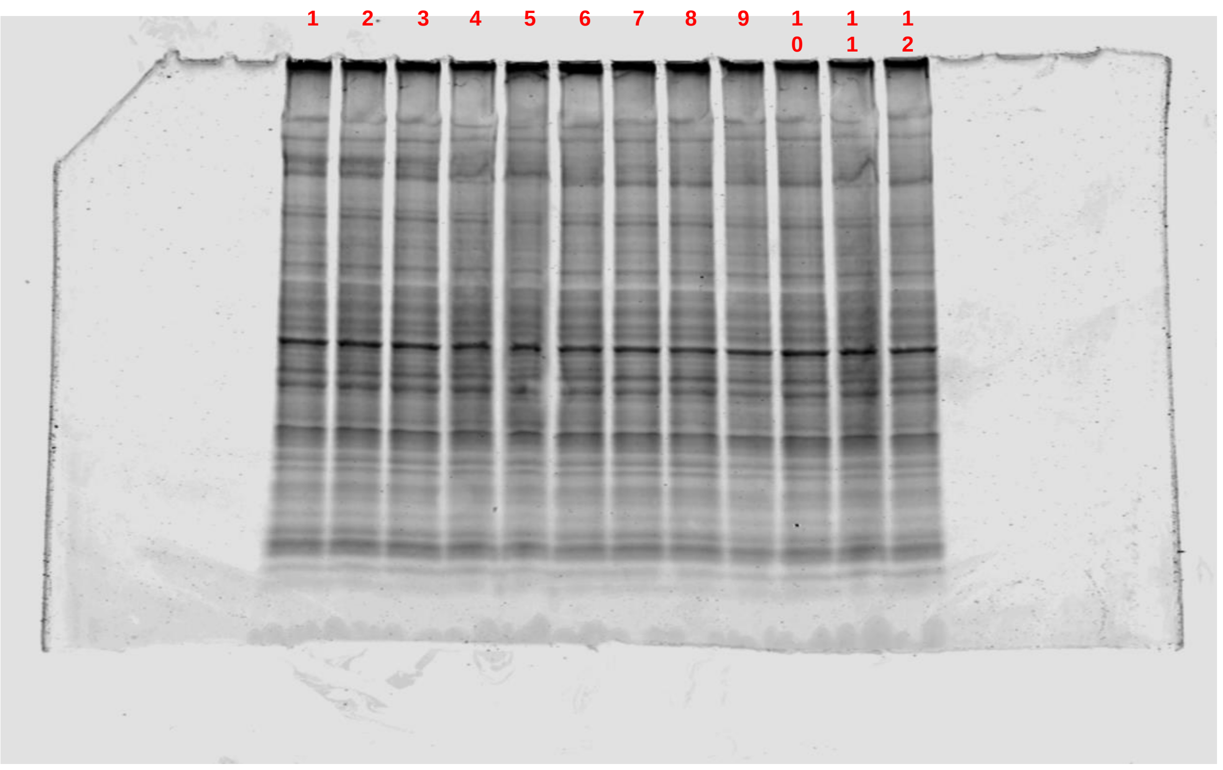

p-S6 (Ser235/236)

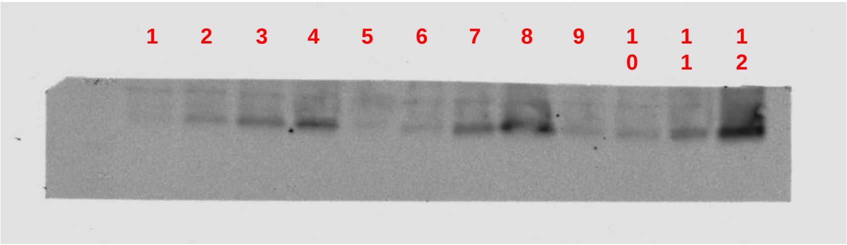

PAN-S6

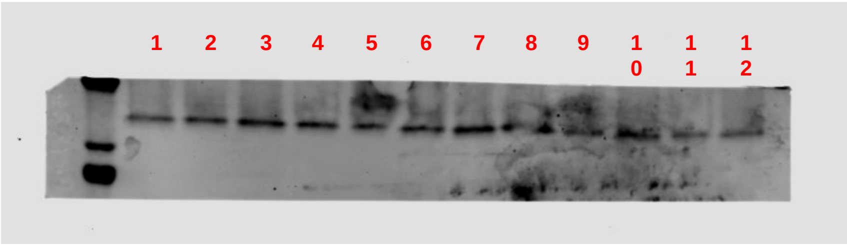

BR2 in Figure 4D quantification

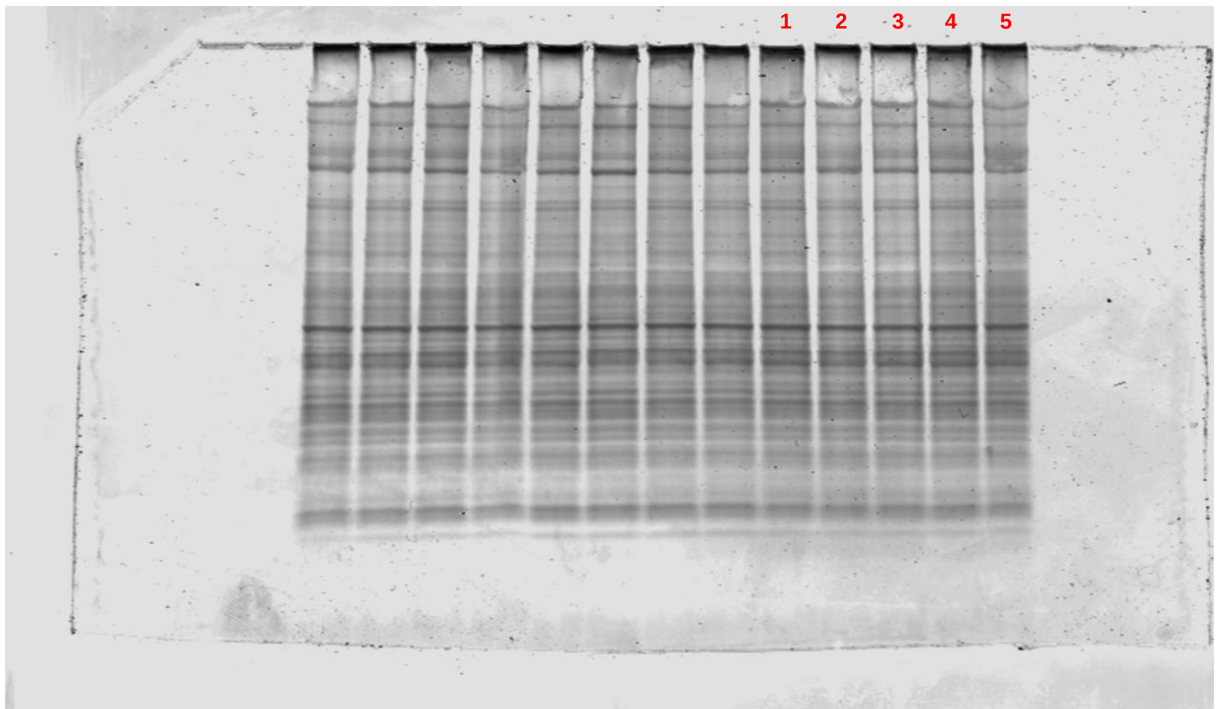

p-AKT (Ser473)

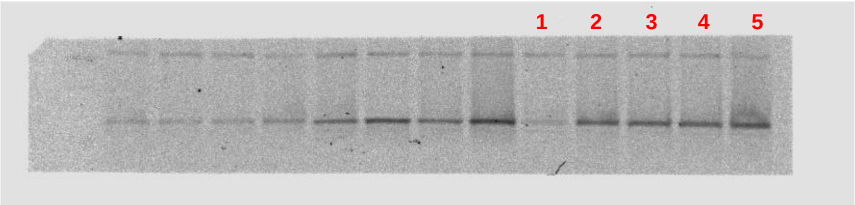

p-S6 (Ser235/236)

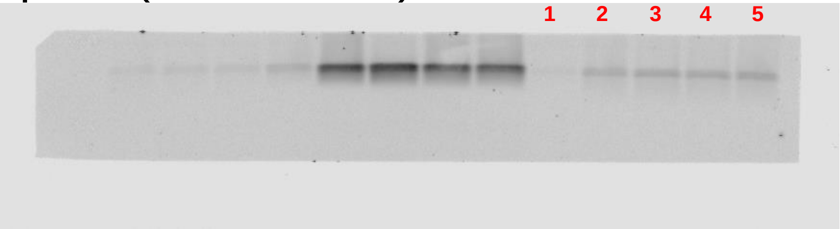

PAN-AKT

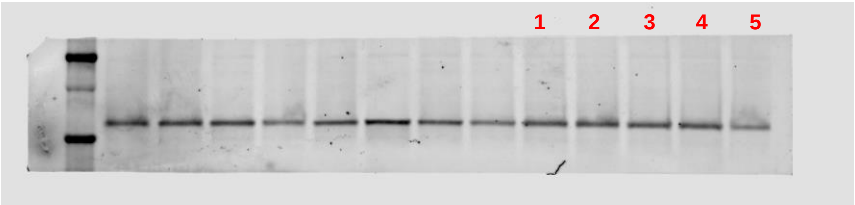

PAN-S6

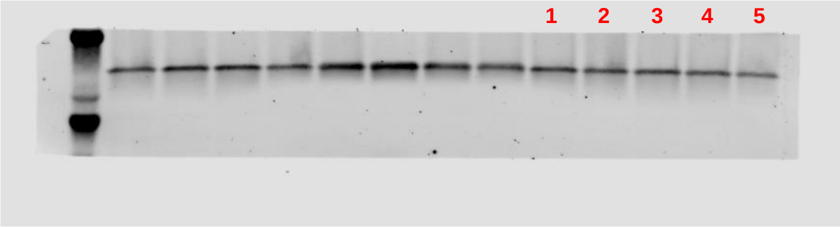

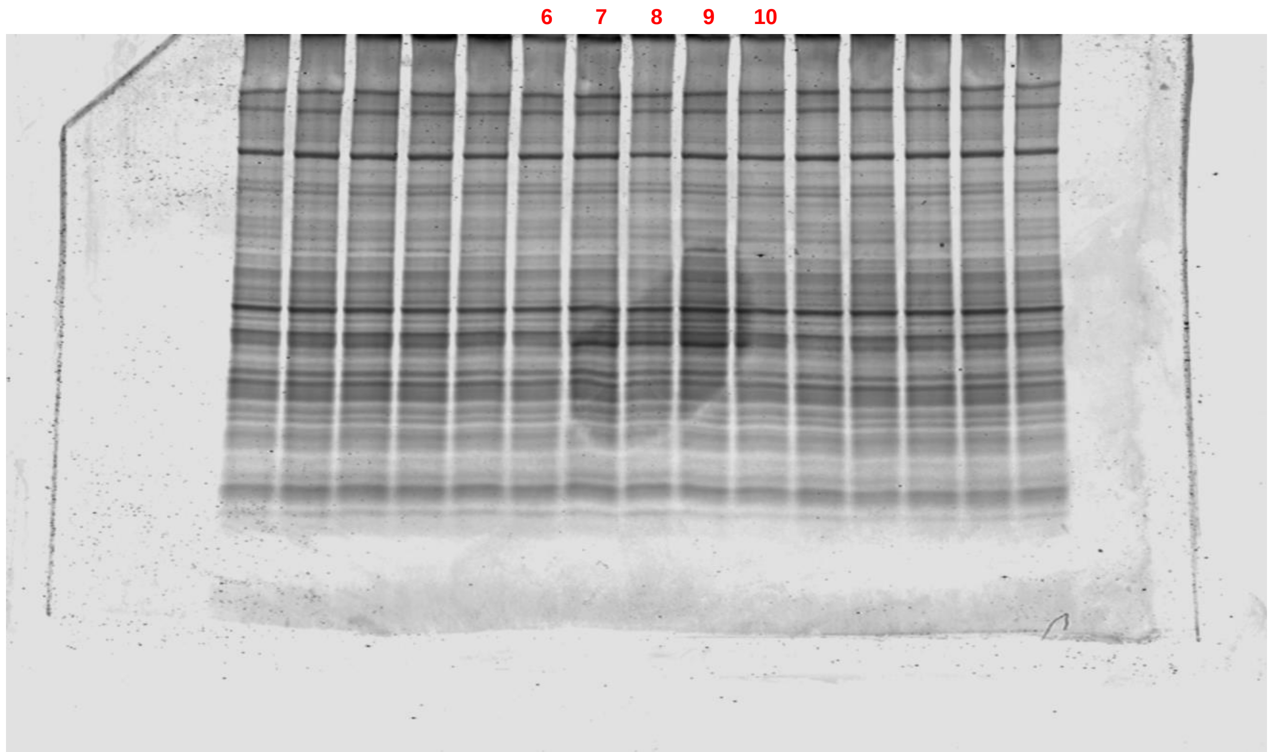

p-AKT (Ser473)

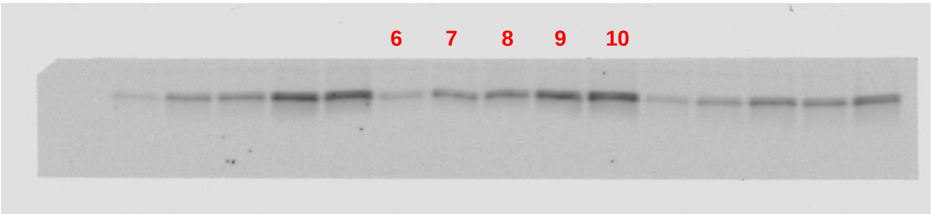

p-S6 (Ser235/236)

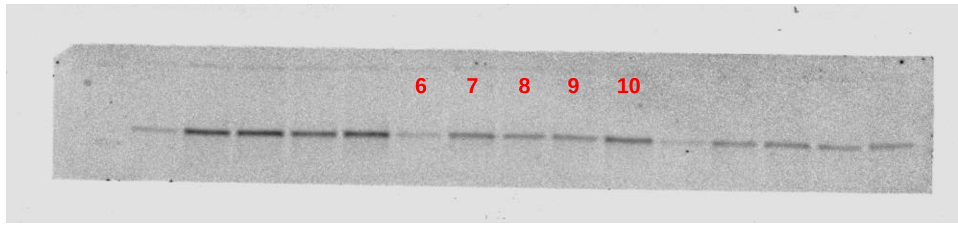

PAN-AKT

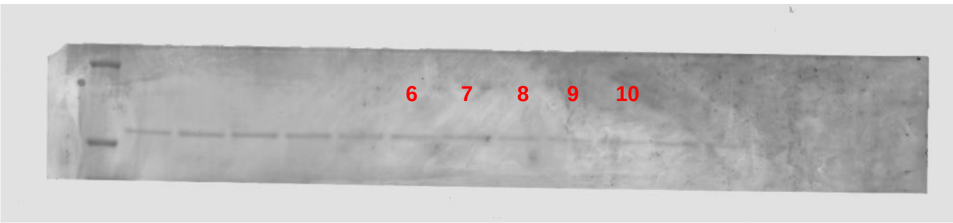

PAN-S6

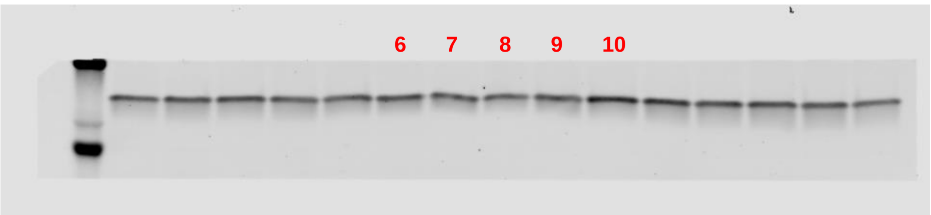

Lane #	Time point (h)	1 nM IGF	Mn (μM)
1*	2	-	0
2		+	0
3		+	0.5
4		+	5
5		+	50
6*	6	-	0
7		+	0
8		+	0.5
9		+	5
10		+	50

* used as loading control for normalization during quantification.

BR3 in Figure 4D quantification

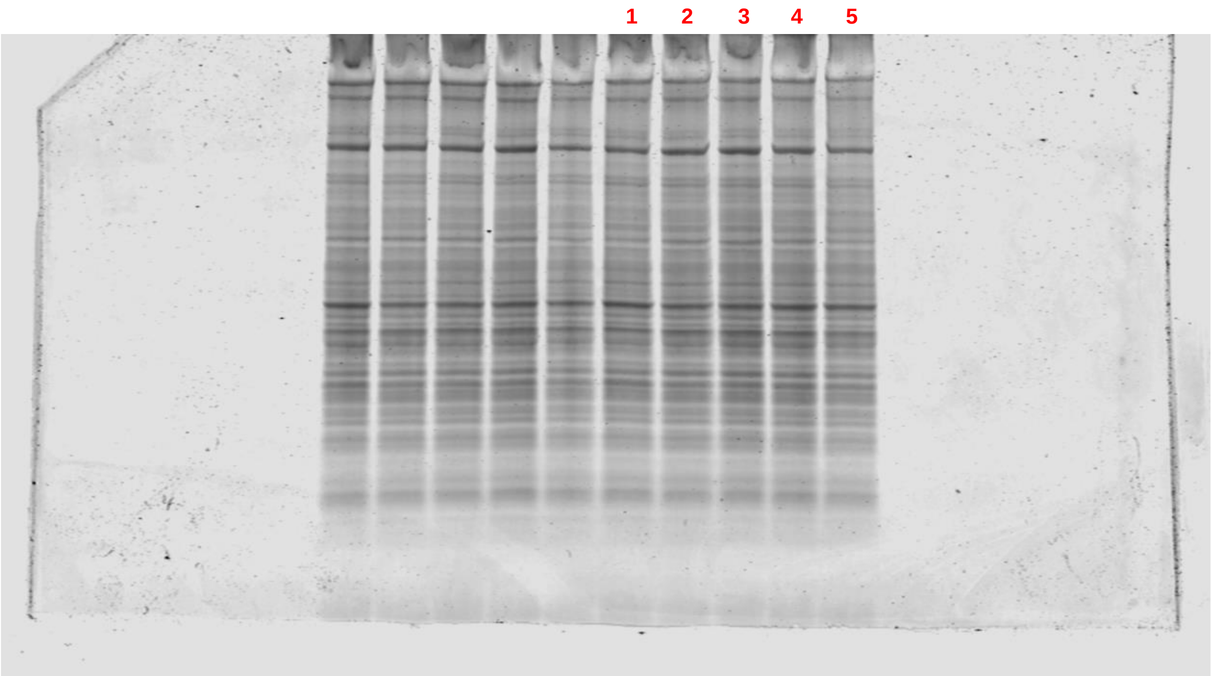

p-AKT (Ser473)

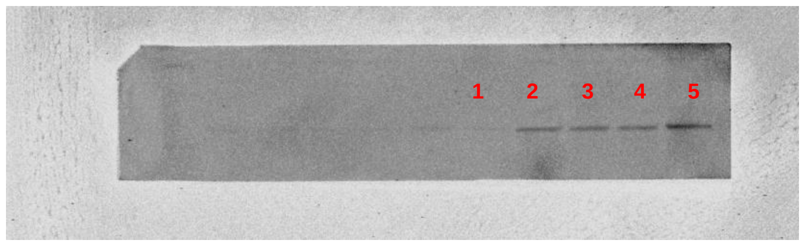

PAN-AKT

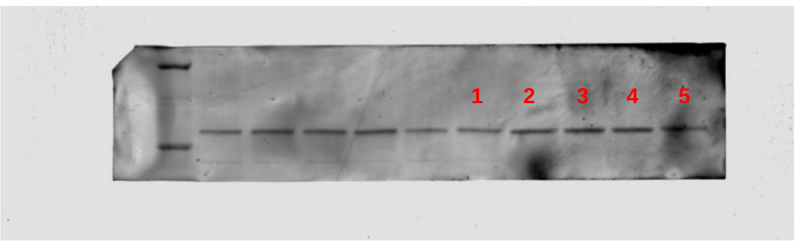

p-S6 (Ser235/236)

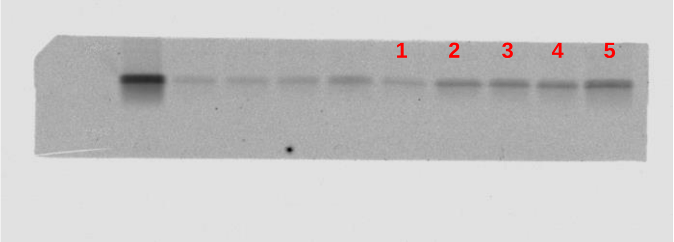

PAN-S6

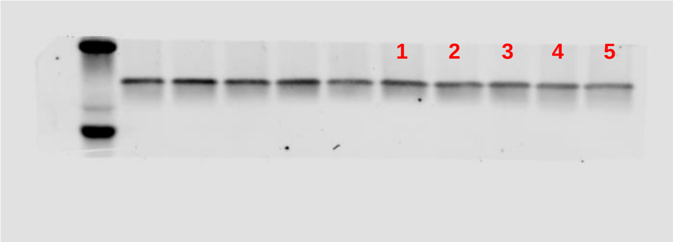

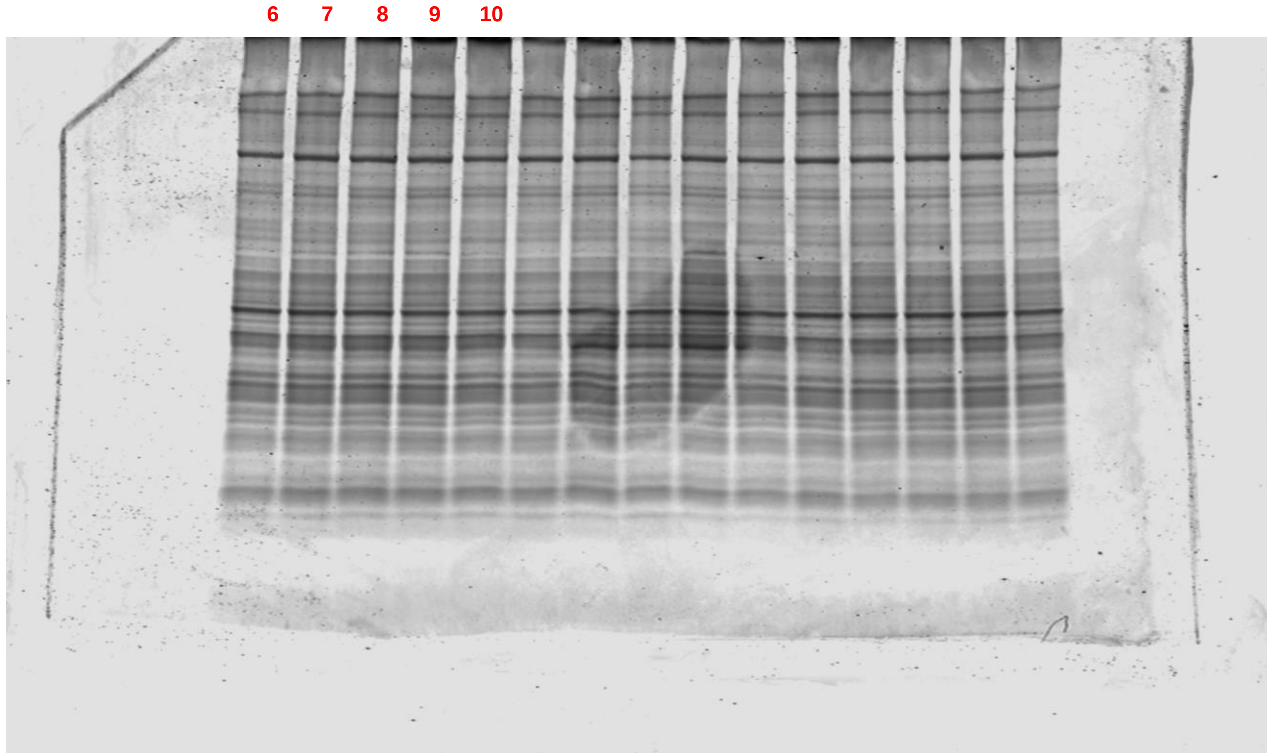

p-AKT (Ser473)

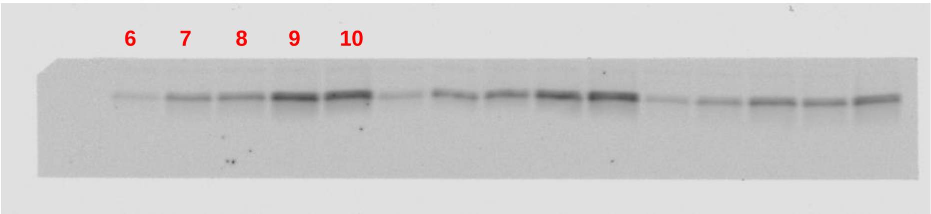

PAN-AKT

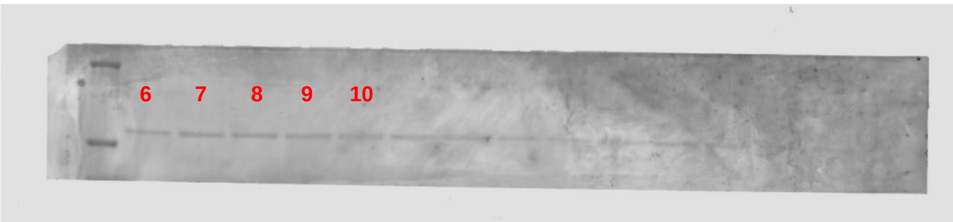

p-S6 (Ser235/236)

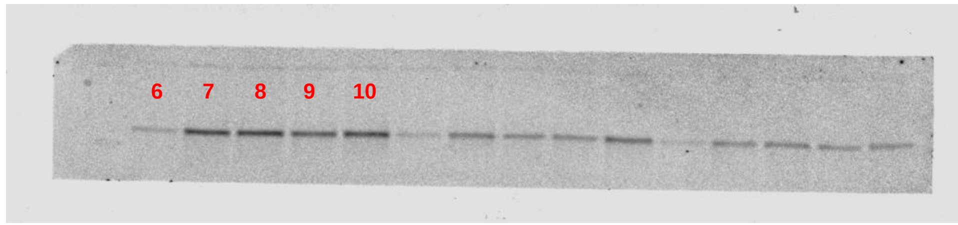

PAN-S6

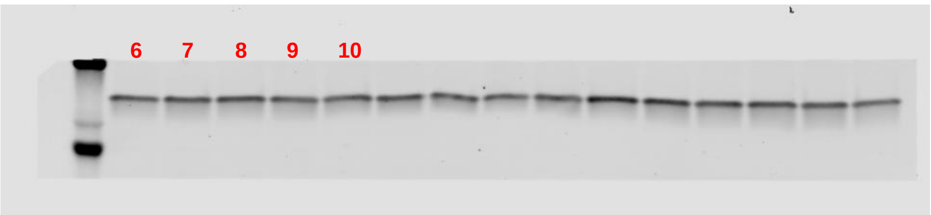

Lane #	Time point (h)	1 nM IGF	Mn (μM)
1*	2	-	0
2		+	0
3		+	0.5
4		+	5
5		+	50
6*	6	-	0
7		+	0
8		+	0.5
9		+	5
10		+	50

* used as loading control for normalization during quantification.

AKT and S6 bands were quantified as BR4 in Figure 4D
mTOR, 4E-BP1, and Actin bands were quantified as BR5 in Supplemental Figure 1

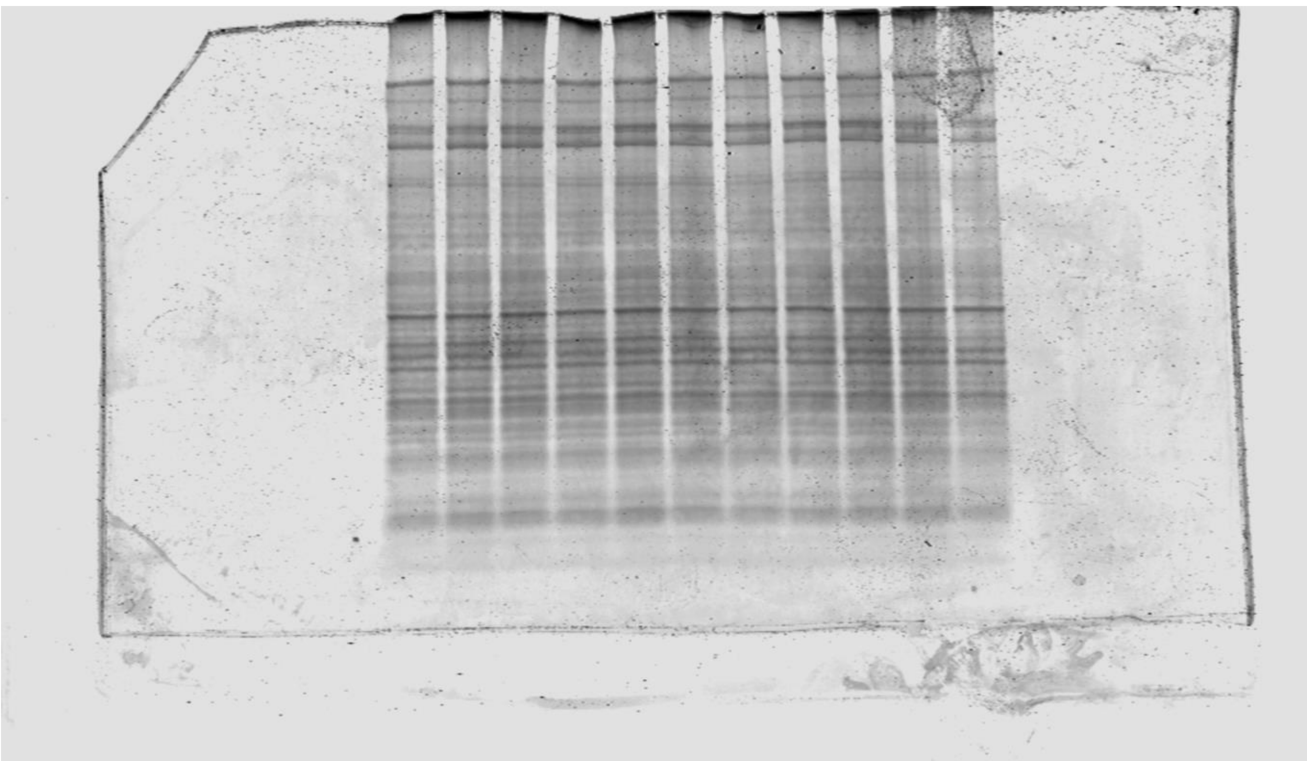

phosphorylated target proteins:

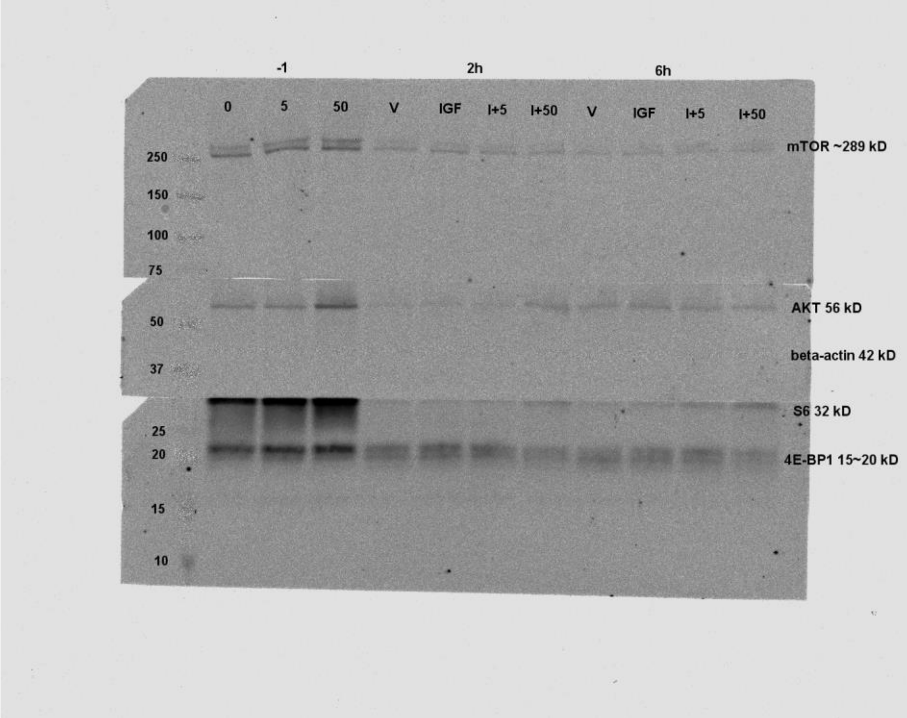

PAN/total target proteins:

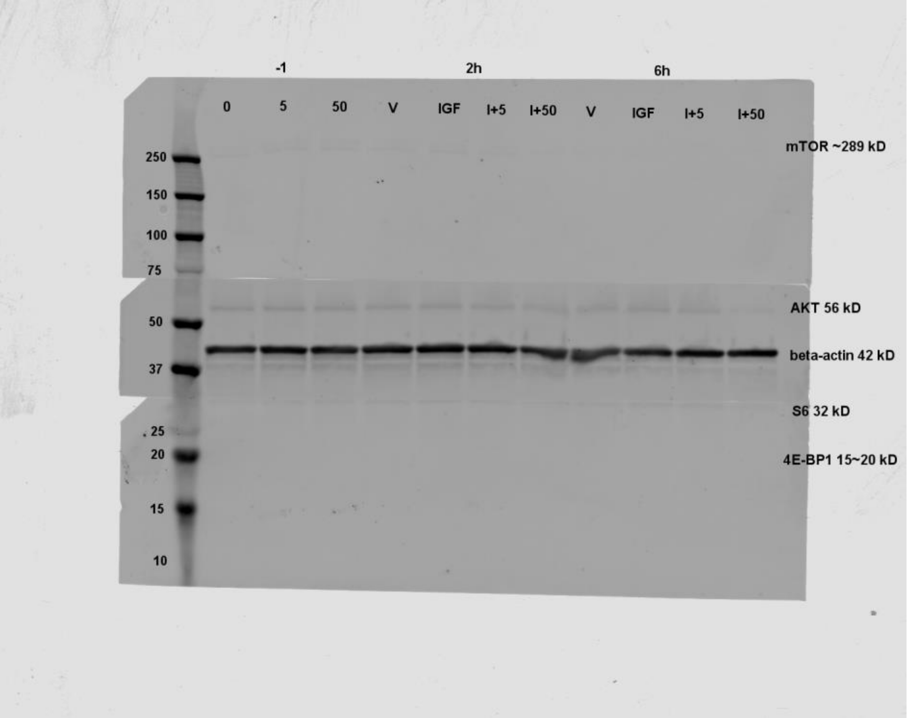

Note: only 2 and 6 hour (h) time points were included in the quantification.

AKT and S6 bands were quantified as BR5 in Figure 4D
mTOR, 4E-BP1, and Actin bands were quantified as BR6 in Supplemental Figure 1

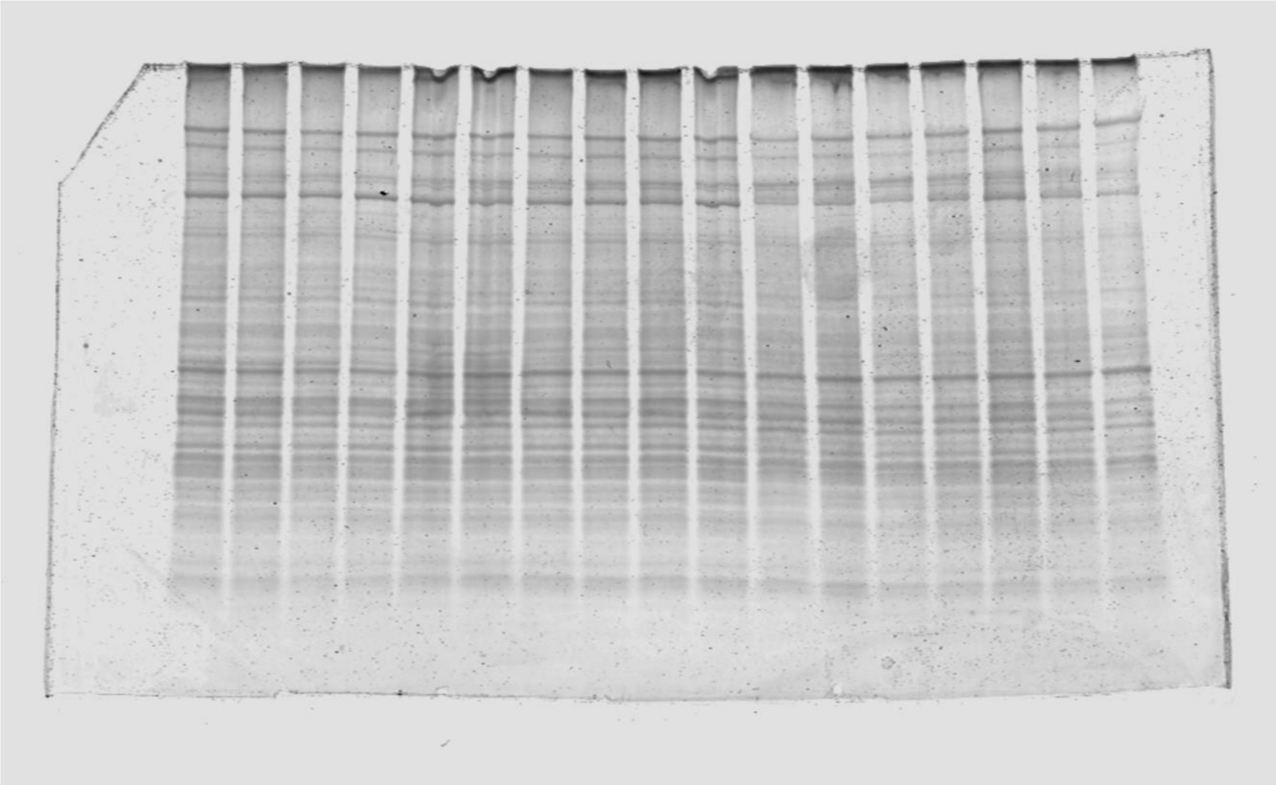

phosphorylated target proteins:

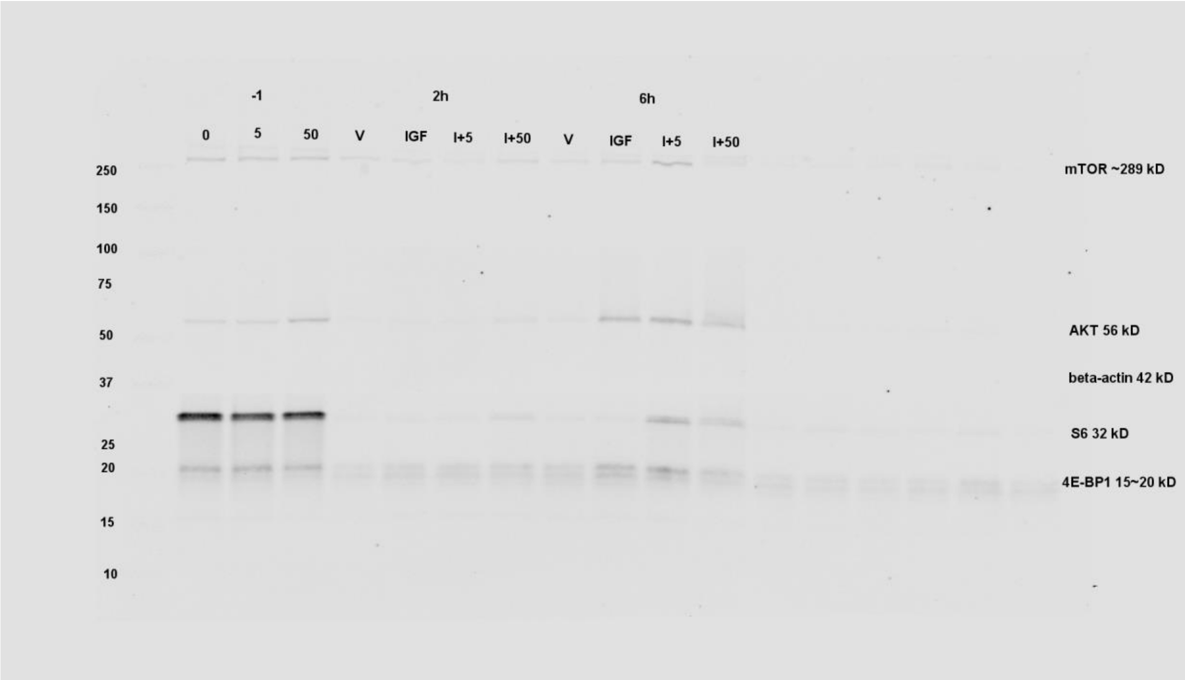

PAN/total target proteins:

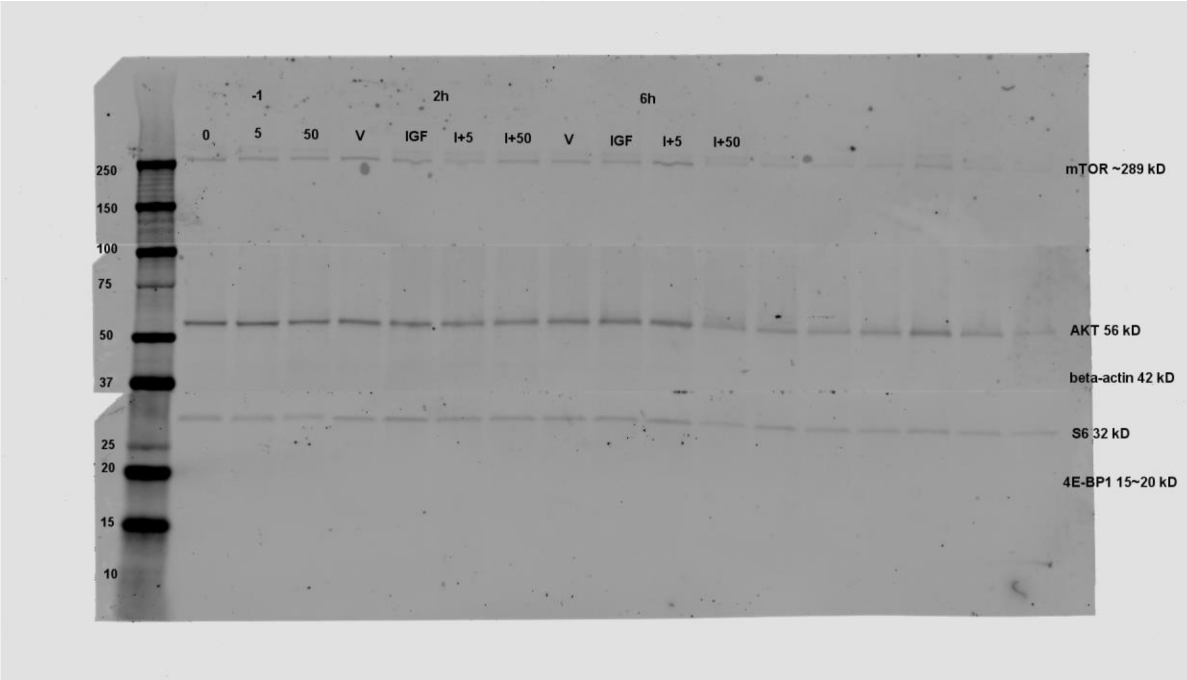

Note: only 2 and 6 hour (h) time points were included in the quantification.

BR2 in Supplemental Figure 1 quantification

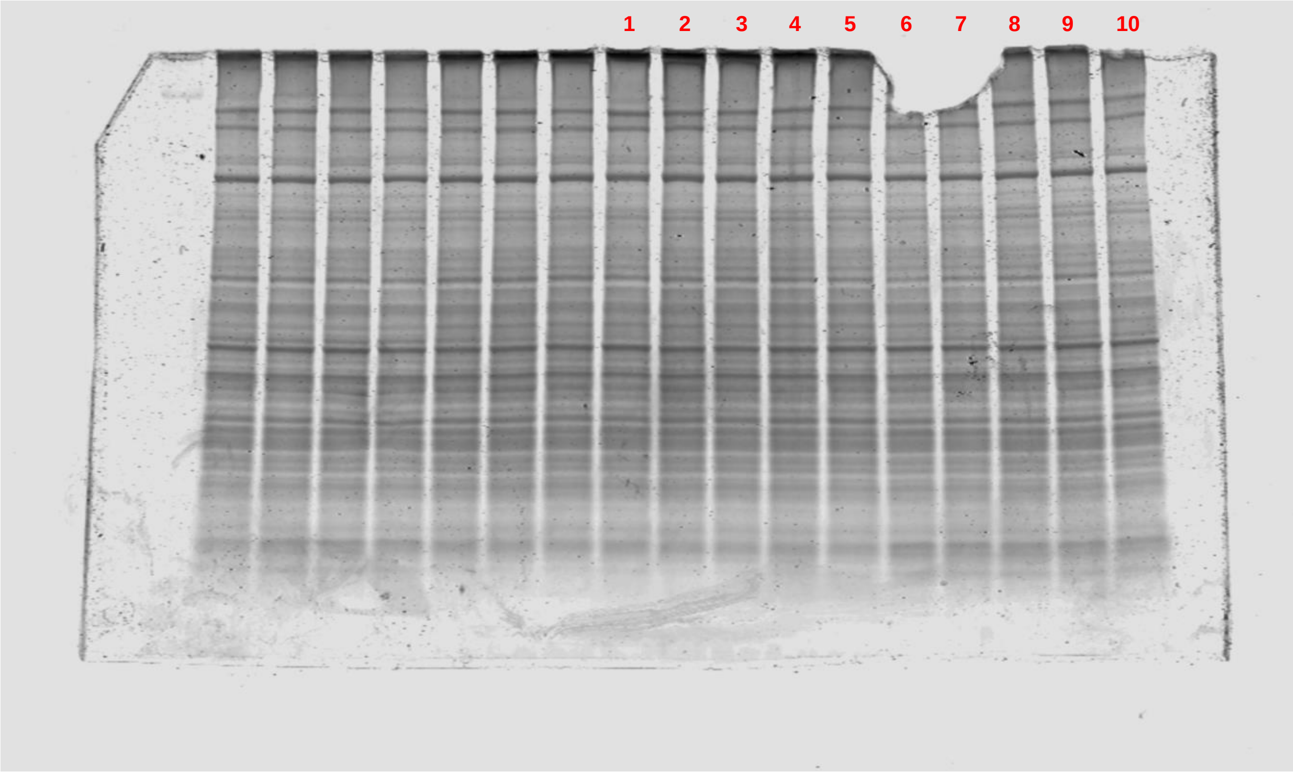

Lane #	Time point (h)	1 nM IGF	Mn (μM)
1		-	0
2		+	0
3	2	+	0.5
4		+	5
5		+	50
6		-	0
7		+	0
8	6	+	0.5
9		+	5
10		+	50

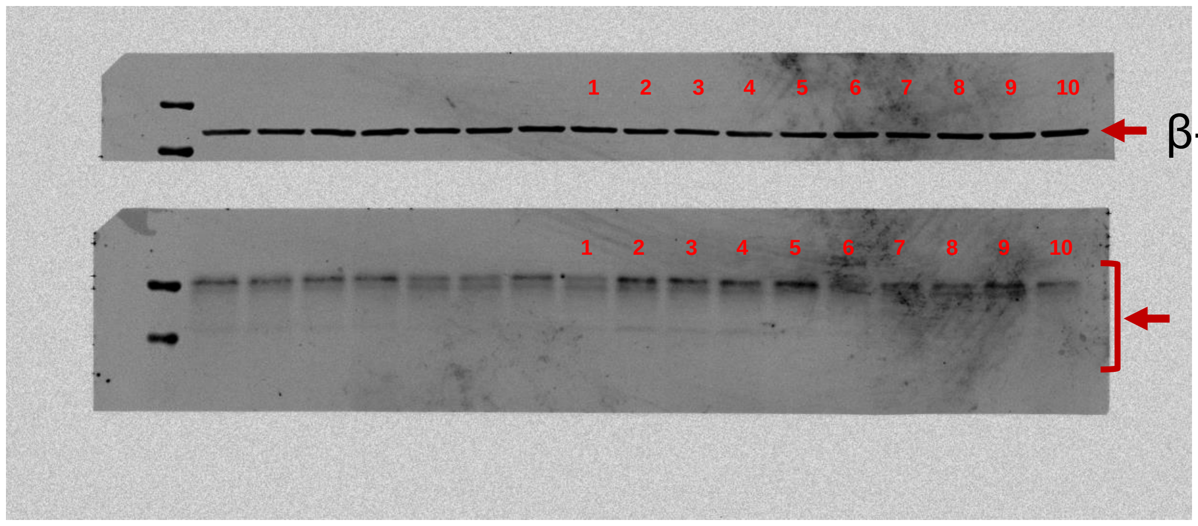

β-Actin (42 kD)

p-4E-BP1 (Thr37/46, 15-20 kD)

p-mTOR (Ser2448)

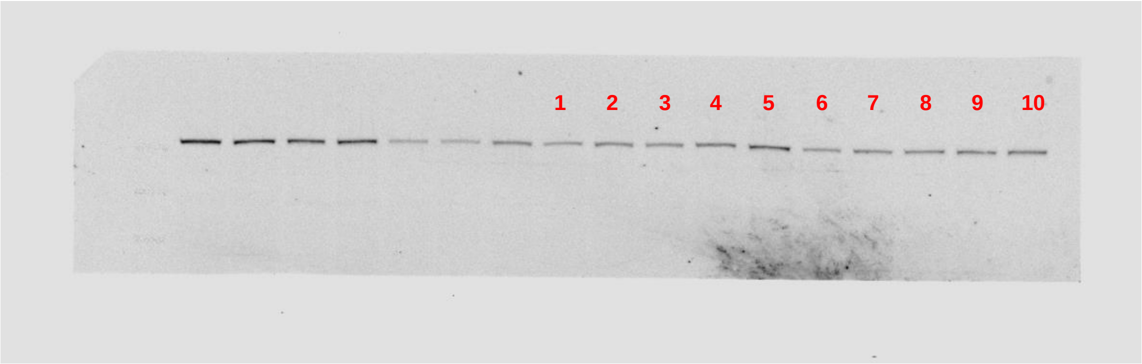

PAN mTOR

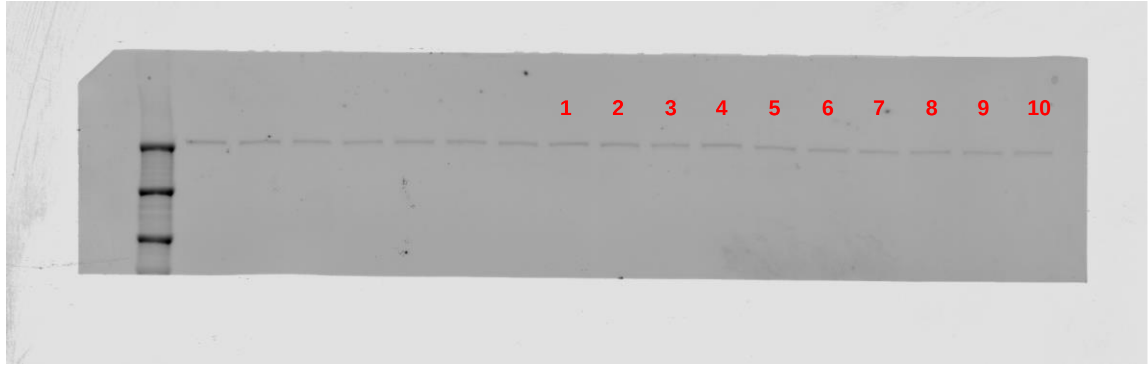

BR3 in Supplemental Figure 1 quantification

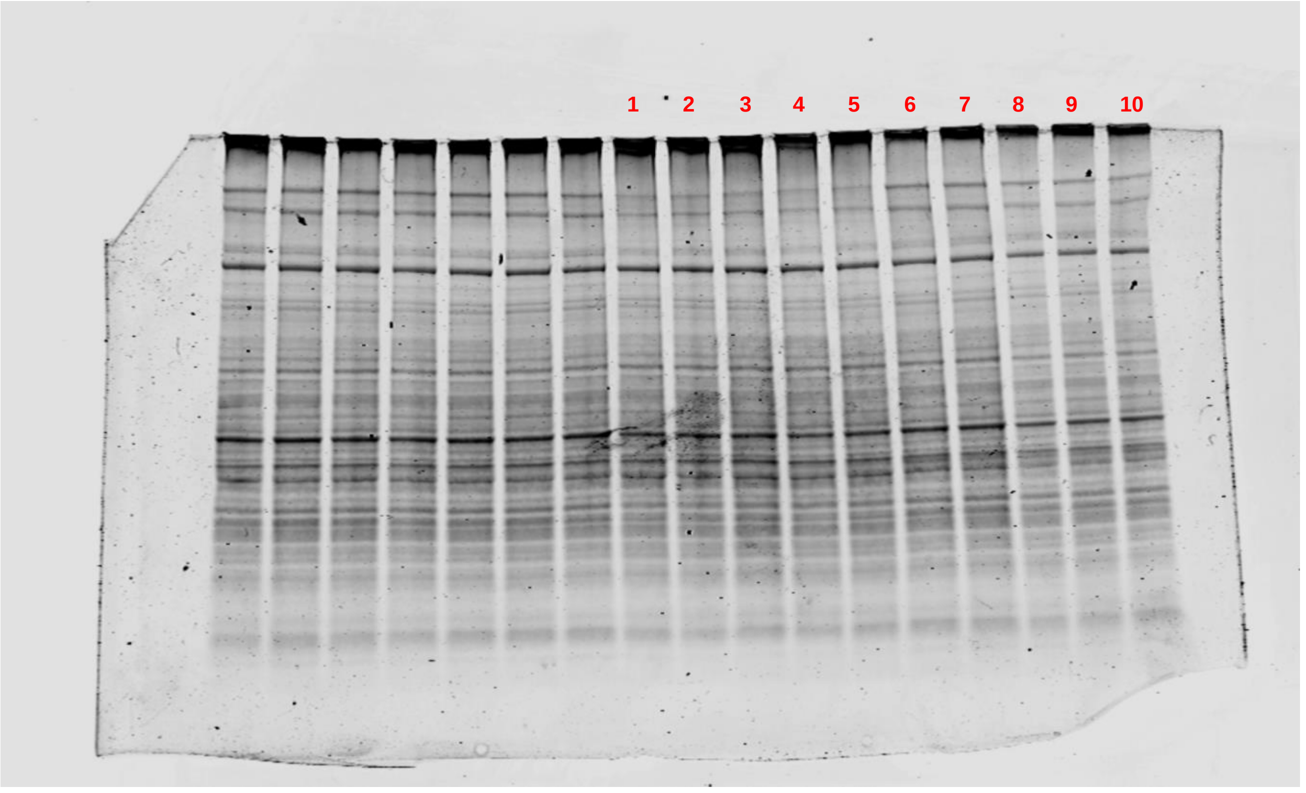

Lane #	Time point (h)	1 nM IGF	Mn (μM)
1		-	0
2		+	0
3	2	+	0.5
4		+	5
5		+	50
6		-	0
7		+	0
8	6	+	0.5
9		+	5
10		+	50

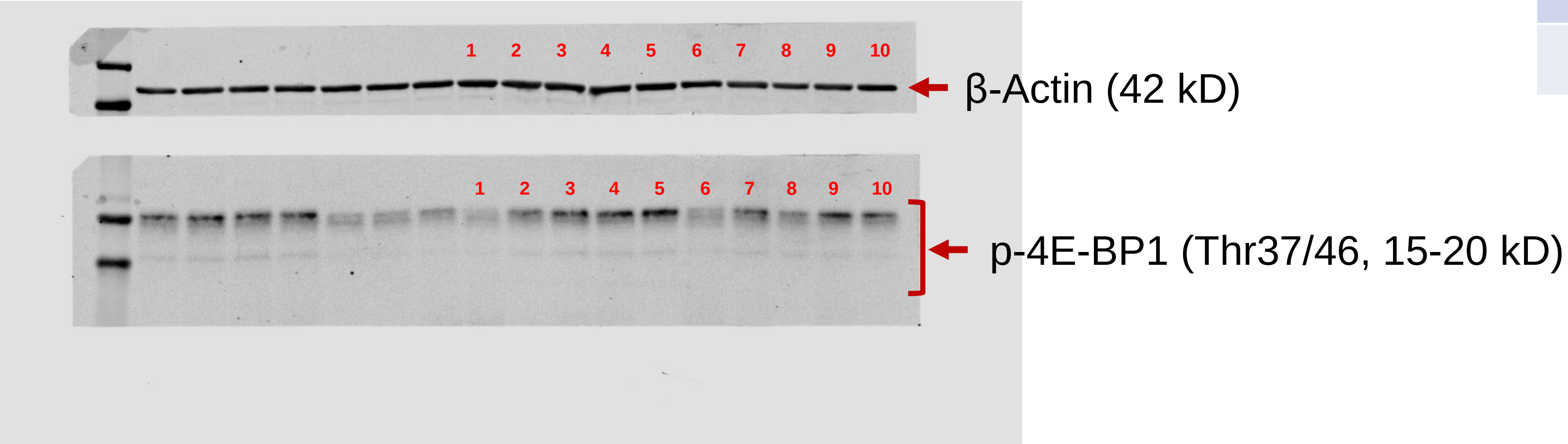

p-mTOR (Ser2448)

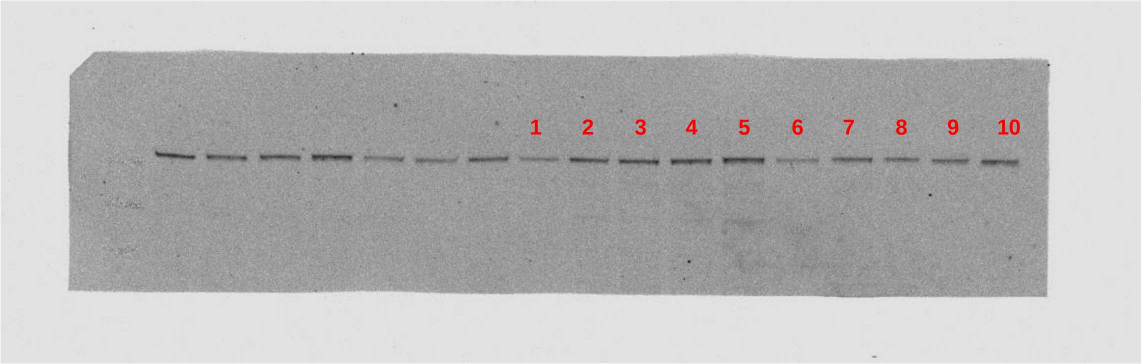

PAN mTOR

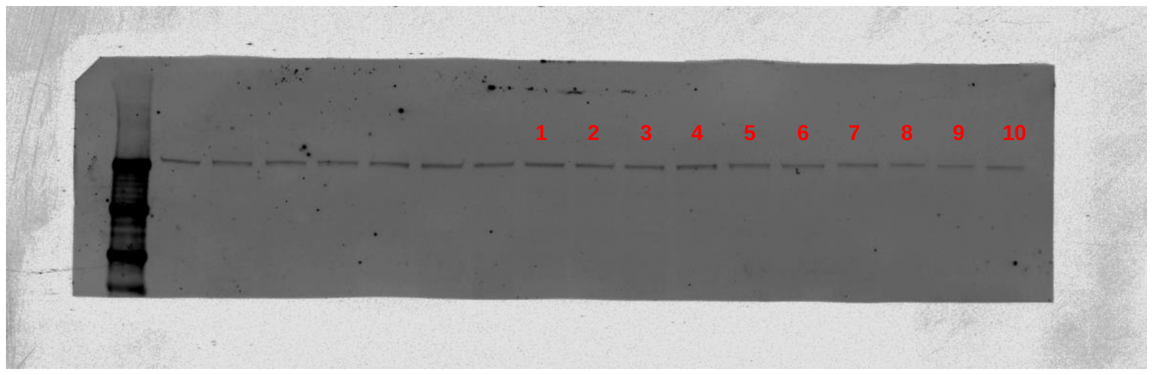

BR4 in Supplemental Figure 1 quantification

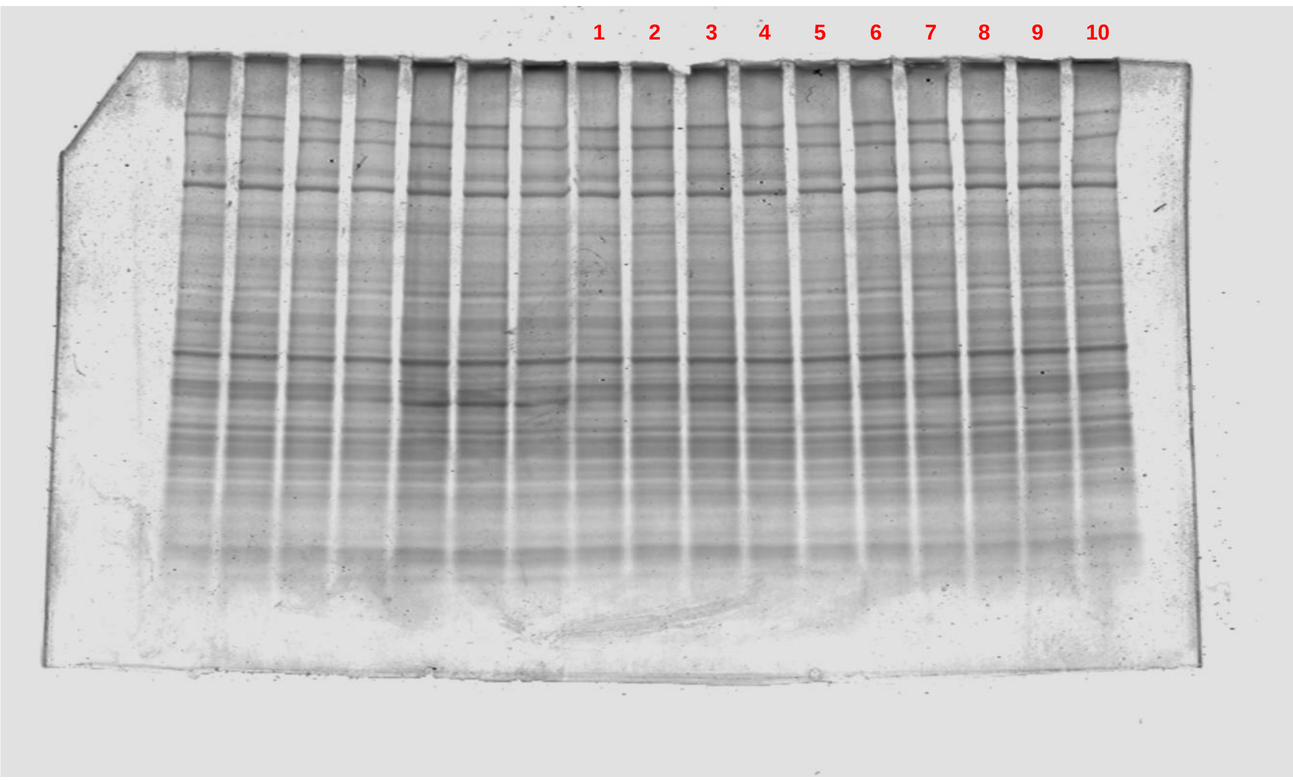

Lane #	Time point (h)	1 nM IGF	Mn (μM)
1		-	0
2		+	0
3	2	+	0.5
4		+	5
5		+	50
6		-	0
7		+	0
8	6	+	0.5
9		+	5
10		+	50

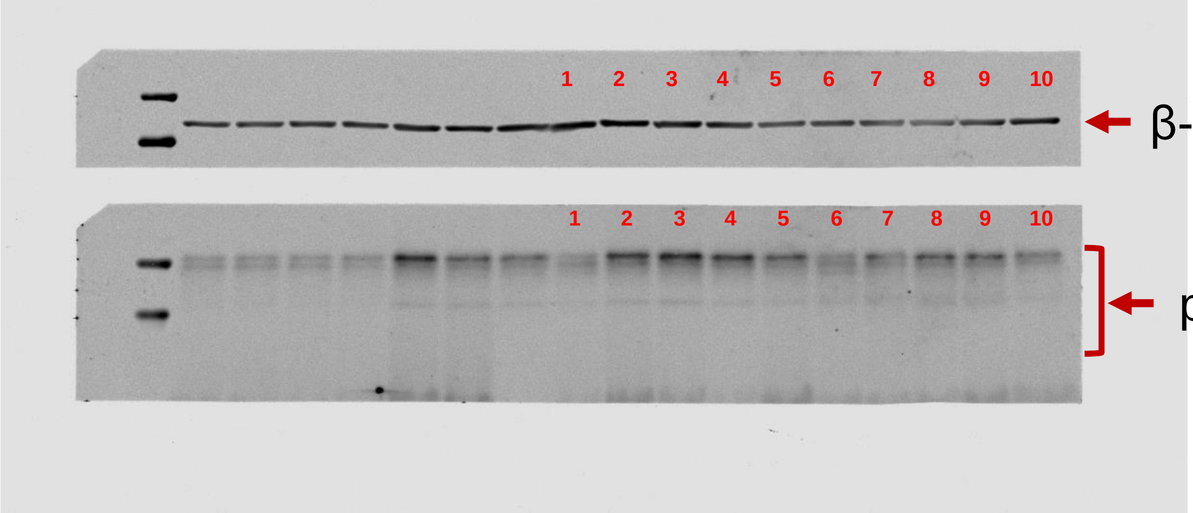

β-Actin (42 kD)

p-4E-BP1 (Thr37/46, 15-20 kD)

p-mTOR (Ser2448)

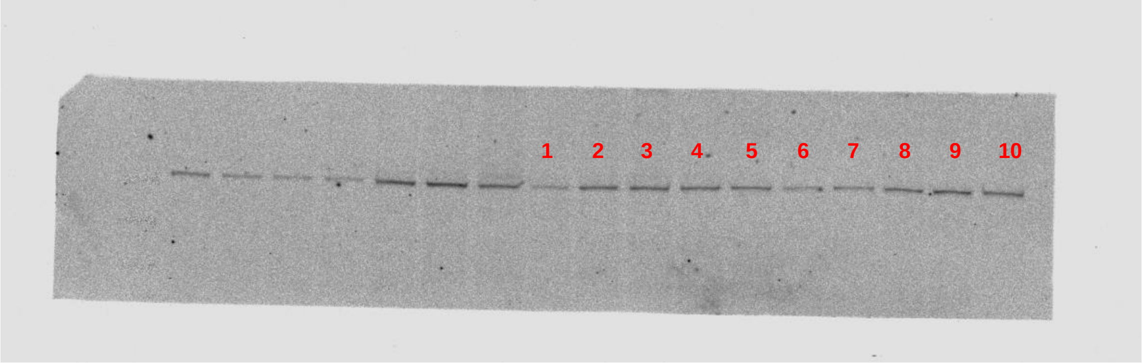

PAN mTOR

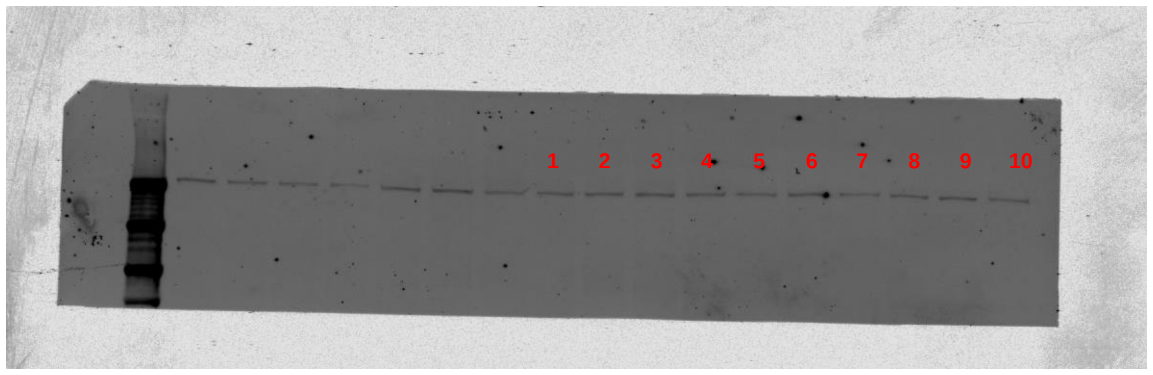

BR4 in Supplemental Figure 1 quantification

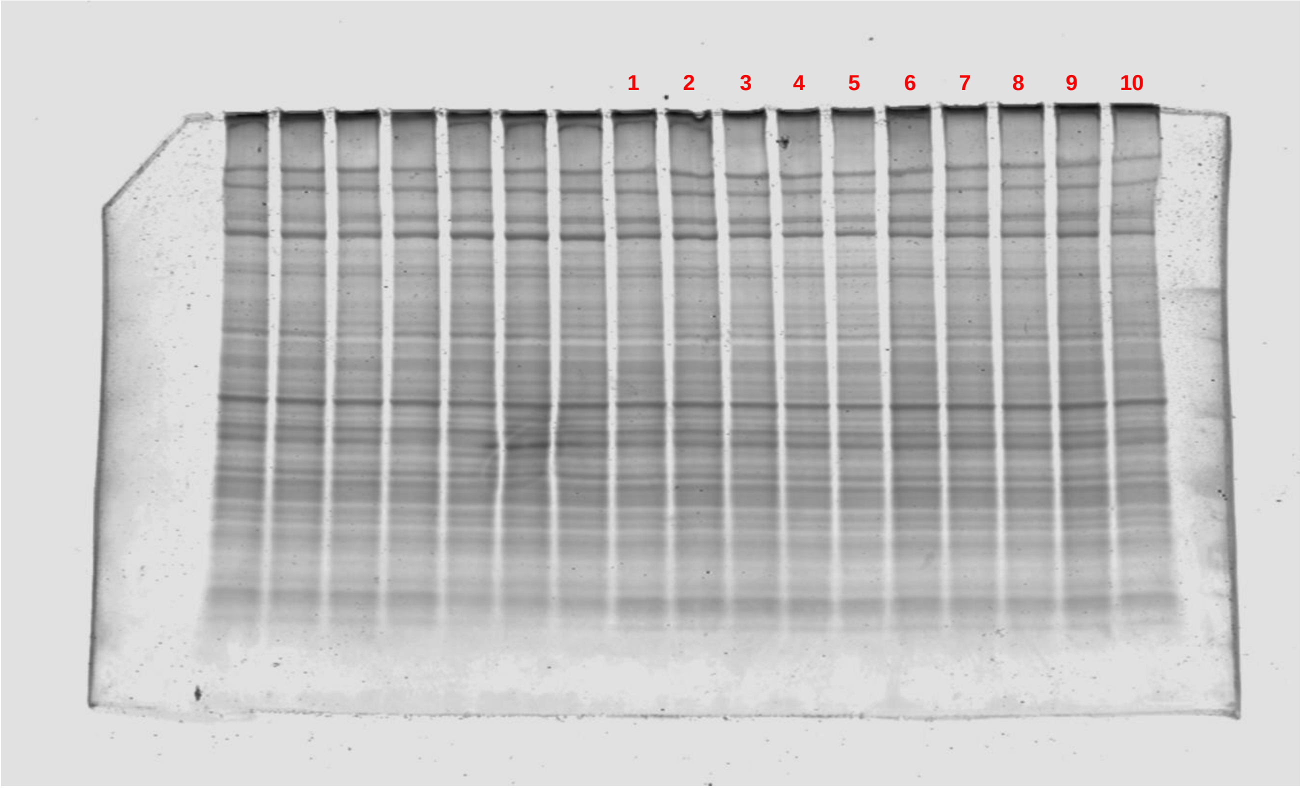

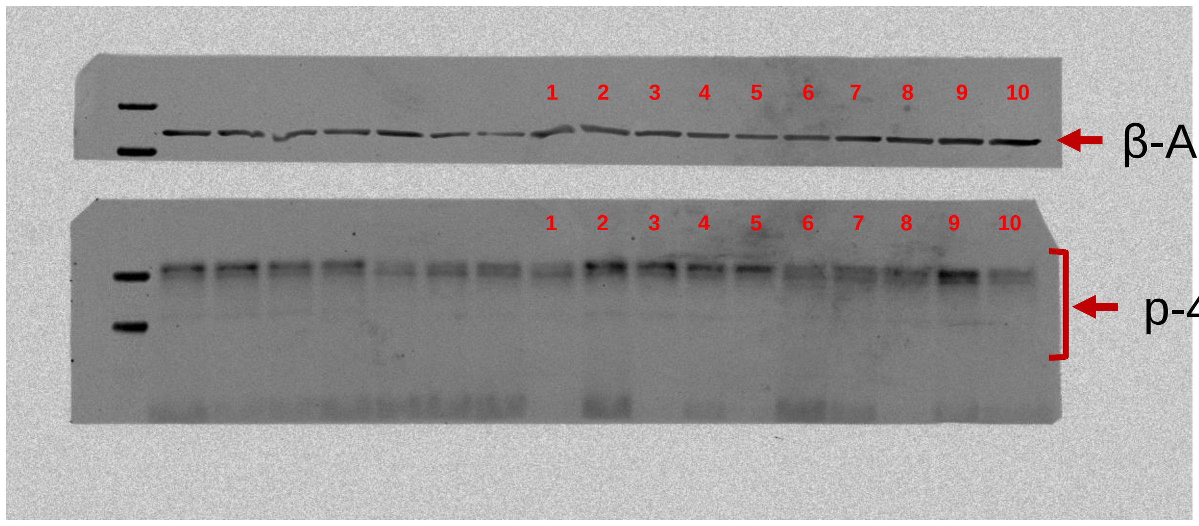

β-Actin (42 kD)

p-4E-BP1 (Thr37/46, 15-20 kD)

Lane #	Time point (h)	1 nM IGF	Mn (μM)
1		-	0
2		+	0
3	2	+	0.5
4		+	5
5		+	50
6		-	0
7		+	0
8	6	+	0.5
9		+	5
10		+	50

p-mTOR (Ser2448)

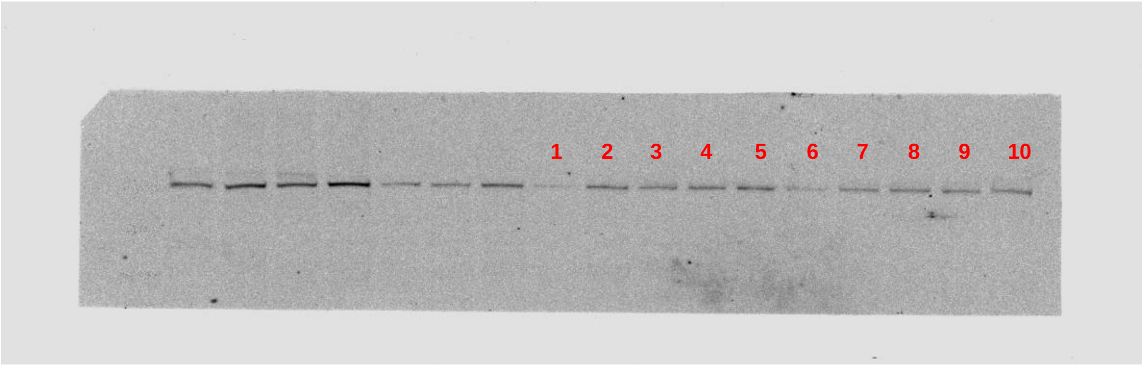

PAN mTOR

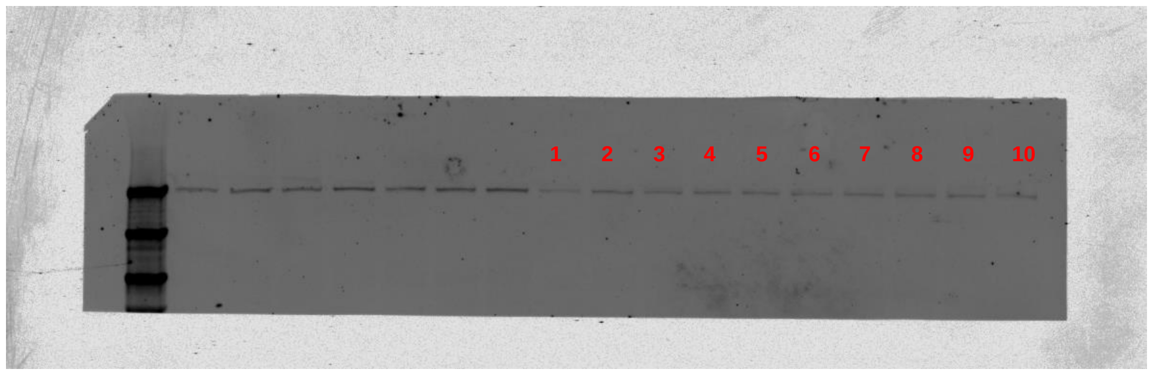

Supplement: Supplementary file 1 [file biomolecules-13-01229-s001.zip › Original Blots_IGF- mTORC1-S6 Signaling Is Potentiated and Prolonged by Acute Loading of Subtoxicological Manganese Ion-file S1.pdf]
