# Supplementary material for: IGF/mTORC1/S6 Signaling Is Potentiated and Prolonged by Acute Loading of Subtoxicological Manganese Ion
Source: Biomolecules. 2023 Aug 8;13(8):1229. doi: 10.3390/biom13081229 (PMC10452562; doi:10.3390/biom13081229)
Supplement: Supplementary file 1 [file biomolecules-13-01229-s001.zip › Supplemental Figure S1.pdf]

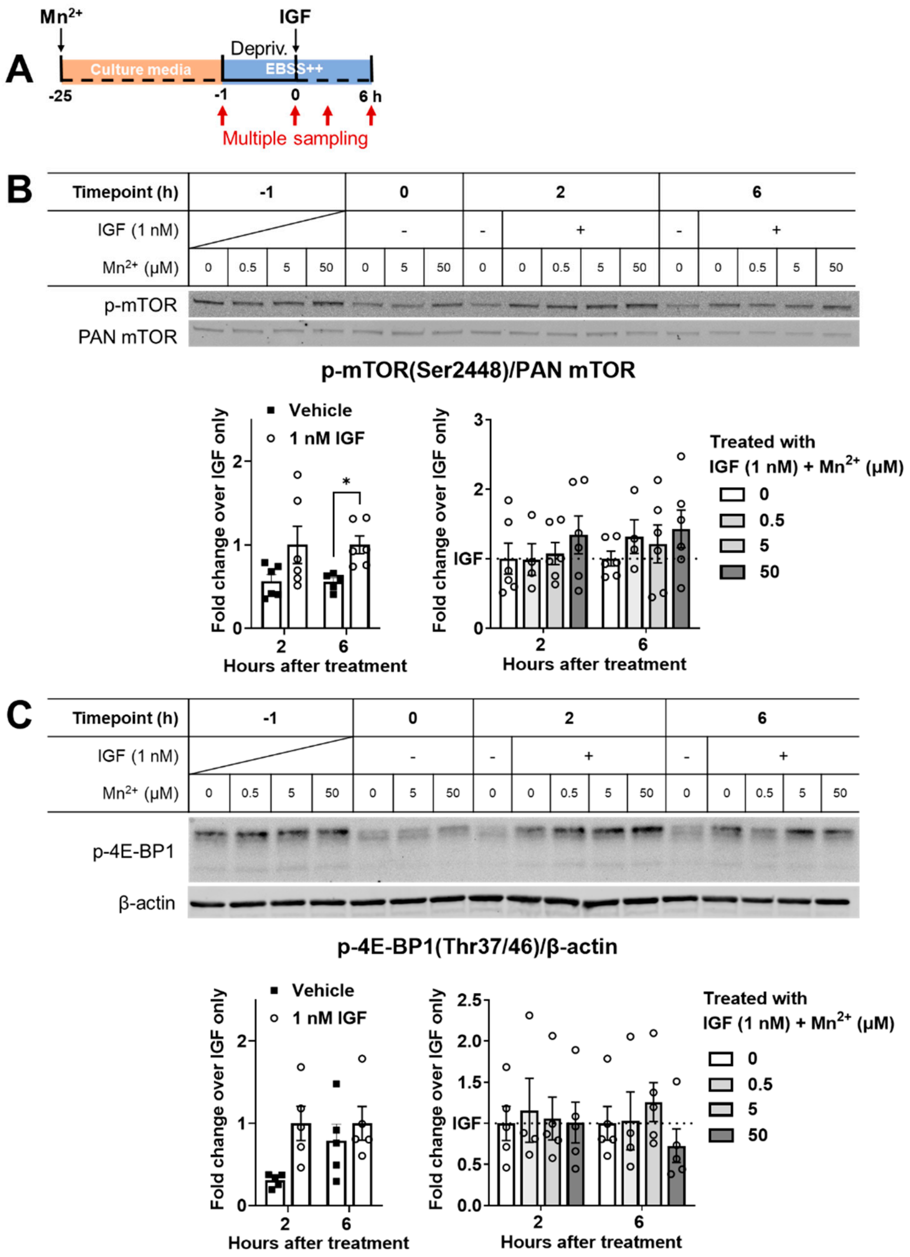

Figure S1. 24-h pre-exposure to low-level Mn exposure followed by IGF stimulation did not significantly affect phosphorylation of mTORC1 at Ser2448 or 4E-BP1 at Thr37/46. **A** Schematic plot of experiment design. mTORC1 and 4E-BP1 phosphorylation were measured from the same set of cell lysates collected in the experiments described in Figure 4. ST*Hdh* Q7/Q7 cells went through a 24-hour pre-exposure with relatively low Mn concentrations ranging from 0, 0.5, 5, and 50 µM. Following the pre-exposure, cells were subject to a 1-hour serum deprivation in EBSS++ before receiving an IGF hit at 1 nM. Mn exposures were continued during the serum deprivation and IGF hit. Cell lysates were collected before the serum deprivation and 0, 2, and 6 h after the start of the treatments. **B** Representative blots and quantification of p-mTOR Ser2448 and PAN mTOR, N=6. An IGF stimulation effect was confirmed at the 6-h time point

represented by significantly elevated p-/PNA ratio (left panel). **C** Representative blots and quantification of p-4E-BP1 Thr37/46 and β-actin, N=6. There was a noticeable fluctuation in 4E-BP1 expression level, and to eliminate the impact, 4E-BP1 phosphorylation was measured by phosphorylated protein intensity normalized to β-actin expression.
